# Randomised controlled trial of oxygen therapy and high-flow nasal therapy in African children with pneumonia

**DOI:** 10.1007/s00134-021-06385-3

**Published:** 2021-05-05

**Authors:** K. Maitland, S. Kiguli, P. Olupot-Olupot, M. Hamaluba, K. Thomas, F. Alaroker, R. O. Opoka, A. Tagoola, V. Bandika, A. Mpoya, H. Mnjella, E. Nabawanuka, W. Okiror, M. Nakuya, D. Aromut, C. Engoru, E. Oguda, T. N. Williams, J. F. Fraser, D. A. Harrison, K Rowan, the COAST trial group, A. Turnbull, A. Odit, E. Molyneux, I. Lubega, W. Macharia, J. Crawley, M. Peters, T. Peto, P. Musoke, F. Were, C. Semple, J. Todd

**Affiliations:** 1 Department of Infectious Disease and and Institute of Global Health and Innovation, Division of Medicine, Imperial College, London, UK; 2 School of Medicine, Makerere University and Mulago Hospital Kampala, Kampala, Uganda; 3 Faculty of Health Sciences, Mbale Campus and Mbale Regional Referral Hospital Mbale (POO, WO), Busitema University, Mbale, Uganda; 4 Kilifi County Hospital and Kenya Medical Research Institute (KEMRI) Wellcome Trust Research Programme, Kilifi, Kenya; 5 Intensive Care National Audit and Research Centre, London, UK; 6 Soroti Regional Referral Hospital, Soroti, Uganda; 7 Jinja Regional Referral Hospital Jinja, Jinja, Uganda; 8 Coast General District Hospital, Mombasa, Kenya; 9 Critical Care Research Group and Intensive Care Service, University of Queensland, The Prince Charles Hospital, Brisbane, Australia

**Keywords:** Oxygen, High-flow nasal therapy, African children, Pneumonia, Clinical trial

## Abstract

**Purpose:**

The life-saving role of oxygen therapy in African children with severe pneumonia is not yet established.

**Methods:**

The open-label fractional-factorial COAST trial randomised eligible Ugandan and Kenyan children aged > 28 days with severe pneumonia and severe hypoxaemia stratum (SpO_2_ < 80%) to high-flow nasal therapy (HFNT) or low-flow oxygen (LFO: standard care) and hypoxaemia stratum (SpO_2_ 80–91%) to HFNT or LFO (liberal strategies) or permissive hypoxaemia (ratio 1:1:2). Children with cyanotic heart disease, chronic lung disease or > 3 h receipt of oxygen were excluded. The primary endpoint was 48 h mortality; secondary endpoints included mortality or neurocognitive sequelae at 28 days.

**Results:**

The trial was stopped early after enrolling 1852/4200 children, including 388 in the severe hypoxaemia stratum (median 7 months; median SpO_2_ 75%) randomised to HFNT (*n* = 194) or LFO (*n* = 194) and 1454 in the hypoxaemia stratum (median 9 months; median SpO_2_ 88%) randomised to HFNT (*n* = 363) vs LFO (*n* = 364) vs permissive hypoxaemia (*n* = 727). Per-protocol 15% of patients in the permissive hypoxaemia group received oxygen (when SpO_2_ < 80%). In the severe hypoxaemia stratum, 48-h mortality was 9.3% for HFNT vs. 13.4% for LFO groups. In the hypoxaemia stratum, 48-h mortality was 1.1% for HFNT vs. 2.5% LFO and 1.4% for permissive hypoxaemia. In the hypoxaemia stratum, adjusted odds ratio for 48-h mortality in liberal vs permissive comparison was 1.16 (0.49–2.74; *p* = 0.73); HFNT vs LFO comparison was 0.60 (0.33–1.06; *p* = 0.08). Strata-specific 28 day mortality rates were, respectively: 18.6, 23.4 and 3.3, 4.1, 3.9%. Neurocognitive sequelae were rare.

**Conclusions:**

Respiratory support with HFNT showing potential benefit should prompt further trials.

**Supplementary Information:**

The online version contains supplementary material available at 10.1007/s00134-021-06385-3.

## Take home message

**Table:** 

In Africa, in children hospitalised with severe pneumonia with oxygen saturations between 80 and 91% who did not receive oxygen, mortality assessed at 48 h (1.4%) was comparable to the usual method of oxygen delivery (low-flow oxygen; LFO (2.5%)) and in those receiving high-flow nasal therapy (HFNT, 1.1%). The potential impact of HFNT on patient-centred outcomes and on resources, particularly oxygen supplies, should stimulate further exploration particularly in children with severe pneumonia managed in low resource settings.	

## Introduction

In Africa, severe pneumonia remains the leading cause of mortality in children under 5 years old [1], posing a major disease burden on health systems. The World Health Organization (WHO) recommends presumptive antibiotic treatment and oxygen for those with clinicallydefined severe pneumonia and/or hypoxaemia (peripheral oxygen saturation (SpO_2_) < 90%) [2]. However, the evidence under-pinning the use of oxygen therapy is weak [3] and it is generally poorly targeted using non-specific clinical signs [4] since many paediatric services lack pulse oximeters [5, 6]. In-hospital mortality among pneumonia cases is high (9–16%) with hypoxemic children at fivefold greater risk of death [6, 7]. WHO recommends research on the targeted use of oxygen therapy together with simple, non-invasive methods of respiratory support as cost-effective strategies for improving outcome [3], but these have not yet been tested in adequately powered randomised controlled trials [8, 9].

A key challenge for the trial design is the significant gap between supply and demand for oxygen in resource-limited African hospitals. Expense and logistic challenges mean that many lack sustainable provision of bottled oxygen [10], as highlighted in a survey of 231 health facilities (12 African countries) showing only 43% had an uninterrupted source of oxygen, 24% had a functioning oxygen concentrator (the WHO-preferred source of oxygen [2]) and an only 81 (35%) had uninterrupted electricity supply (necessary for oxygen concentrators) [11]. Thus, in many hospitals children with severe pneumonia receive little or no oxygen, whether this contributes to the poor outcomes is unknown.

The Children’s Oxygen Administration Strategies Trial (COAST) simultaneously addressed two hypotheses. First, whether liberal oxygenation strategies in children with SpO_2_ ≥ 80–91% will decrease mortality (at 48 h and up to 28 days) compared with a permissive hypoxia strategy. Second, whether respiratory support with high-flow nasal therapy (OptiFlow^™^) decreases mortality (at 48 h and up to 28 days) compared with low-flow oxygen delivery (standard care) [12].

## Methods

COAST was a two-stratum multicentre, open, fractional-factorial RCT (see statistical methods**, **Supplemental Appendix) conducted in four Ugandan and two Kenyan hospitals. Children aged 28 days to 12 years, hospitalised with a history of respiratory illness and any one of the 2013 WHO clinical definitions of severe pneumonia [13] plus hypoxaemia (SpO_2_ < 92%) were enrolled into either the severe hypoxaemia stratum (SpO_2_ < 80%) or the hypoxaemia stratum (SpO_2_ 80–91%). Children with previous diagnosed but uncorrected cyanotic heart disease, chronic lung disease (excluding asthma), children given oxygen given at another health facility (or > 3 h at the current hospital) or previous COAST enrolment were excluded. In the severe hypoxaemia stratum, eligible children were randomised (ratio 1:1) to high-flow nasal therapy (HFNT) via OptiFlow^™^ or low-flow oxygen delivery (LFO: standard practice). In the hypoxaemia stratum, eligible children were randomised (ratio 1:1:2) to HFNT via OptiFlow^™^, LFO delivery (standard practice) or permissive hypoxaemia since pre-existing data indicated no differences in mortality across the SpO_2_ range 80–89% [12, 14] (see Supplemental Appendix and Trial Protocol).

### Screening and randomisation

Children hospitalised with suspected severe pneumonia were clinically assessed for eligibility including oxygen saturation measurement (BITMOS sat 801 +), which are capable of measuring oxygen saturations accurately during motion and low peripheral perfusion. Children eligible for the hypoxaemia stratum required two SpO_2_ readings of 80–91% 5 min apart. Where prior written consent from parents/legal guardians could not be obtained, ethics committees approved verbal assent with delayed written informed consent as soon as practicable [15]. Otherwise informed written consent was obtained from parents or guardians before randomization. The trial statistician in London generated and kept the sequential randomization list, computer-generated using variably sized permuted blocks stratified by trial centre. Randomisation occurred using consecutively numbered packs containing randomised links to opaque sealed envelopes ensuring allocation concealment.

### Study procedures

Children were managed on general paediatric wards; mechanical ventilation facilities were largely unavailable. Training in triage and emergency paediatric life support was given throughout the trial to optimize case recognition, supportive management and protocol adherence. Basic infrastructural support for emergency care, patient monitors, haemoglobin, glucose and lactate point-of-care tests, blood cultures and chest X-rays were provided by the study. A structured clinical case report form was completed at admission and on reviews at 1 2, 4, 8, 16, 24, 36 and 48 h.

### Oxygen therapy and respiratory support

HFNT was delivered by AIRVO^™^2 device (https://www.fphcare.com/), which contains a humidifier with integrated flow generator that delivers, to spontaneous breathing patients, high flow warmed and humidified air/oxygen blend. HFNT was initiated on FiO_2_ of 21% (room air) with flow rates increase and oxygen titrated in using a structured protocol. Reliable sources of oxygen including electricity power-back up for the AIRVO^™^2 and oxygen concentrators were provided to ensure oxygen delivery was uninterrupted [11]. LFO was delivered by nasal canulae/prongs and escalated to higher flow rates delivered by standard masks. Saturations were checked at 15-, 30-, and 60-min post-enrolment and during the structured reviews. Per-protocol the permissive hypoxaemia arm received LFO if SpO_2_ fell below 80%. Children unable to tolerate HFNT were switched to LFO. From 2-h post-enrolment oxygen could be weaned/stopped if SpO_2_ remained ≥ 92% in room air and restarted if SpO_2_ dropped to < 92%. At 48 h children on HFNT were switched to LFO (extra details in Supplemental Methods).

All children received standard treatments including intravenous maintenance fluids (2.5–4 mls/kg/hour) [16], antibiotics, antimalarials, antipyretics, anticonvulsants, and transfusion for haemoglobin < 5 g/dl according to national guidelines. At the scheduled follow-up visit, children were clinically assessed (including neurodevelopmental assessment) at 28 day post-randomization. Clinicians were trained on the structured ‘Kilifi Developmental Milestones Assessment which covers three broad domains of child functioning: motor, language and personal–social development [17]. From previous experience [18], neurocognitive changes can be transient, children who exhibiting neurocognitive sequalae at 28 days were re-assessed at 90 days. Nurses/doctors were unblinded; laboratory tests were assayed blinded.

### Endpoints

The primary outcome was mortality at 48-h post-randomization (a timepoint capturing the majority of in-patient deaths [16]) and deaths to Day 28 [19]. Secondary outcomes included day-28 mortality, treatment failure at 48 h (persistent hypoxaemia: SpO_2_ < 92% plus respiratory distress), time to hypoxaemia resolution (SpO_2_ ≥ 92%), duration of respiratory (oxygen/HFNT) support, length of initial hospital stay, Day-28 neurocognitive/developmental sequelae, re-admissions and anthropometric status (See Statistical Analysis Plan). Adverse events were graded using the Common Toxicity Criteria for Adverse Events v4.0 [20]. An Endpoint Review Committee (ERC) reviewed all deaths blinded to treatment arm.

### Statistical analysis

The sample size was determined using simulations, assuming a 1:2 ratio between the respective strata and 48 h mortality in the LFO arms of 26 and 9% in severely hypoxaemia and hypoxaemia strata respectively [12]. Overall, 4200 children provided at least 90% power to detect a 33% relative risk reduction (RR) for liberal (HTNT or LFO) vs permissive hypoxaemia and a 25% RR for HFNT vs. LFO. Two (of three) planned interim analyses were reviewed by an independent Data Monitoring Committee using Haybittle–Peto criterion (*p* < 0.001) for early stopping for either efficacy or harm.

Patients were analysed according to their randomised groups, following a prespecified statistical analysis plan. The primary outcome was analysed as a binary outcome using multilevel logistic regression including both treatment allocation variables simultaneously (HFNT vs. LFO, and liberal (any respiratory support) vs. permissive hypoxaemia and adjusted for the stratifying factors of baseline SpO_2_ (grouped as < 80, 80–84, 85–89, 90–91%) and trial site (as a random factor). Adjusted odds ratios (aOR) with 95% confidence intervals (CI) were calculated to compare any respiratory support/oxygen vs permissive hypoxaemia (hypoxaemia stratum only), and HFNT vs. LFO (primary effect estimates). Each treatment allocation variable was tested for significance using a likelihood ratio test of the reduced compared to full regression model fit. (More details in Supplemental Methods).

## Results

The trial was stopped prematurely in by the Trial Steering Committee on the grounds of feasibility as a result of a campaign to terminate the trial in Uganda, which deemed permissive hypoxaemia (permissive hypoxaemia) unethical (detailed in the Supplemental Methods).

**Fig. 1:**
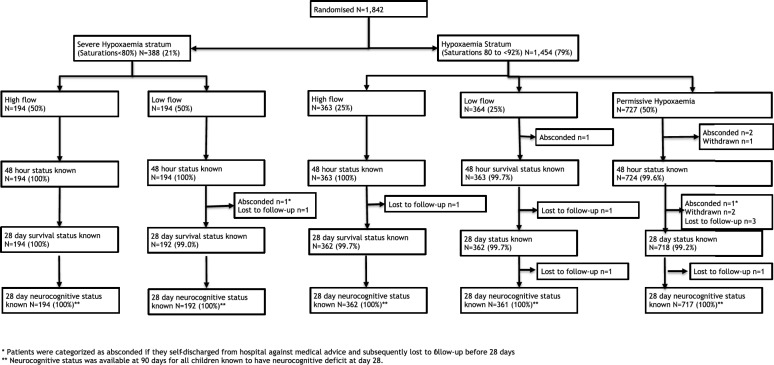
Asterisk patients were catagorised as absconded if they self-discharged from hospital against medical advice and subsequently lost to follow-up before 28 days. Double asterisk neurocognitive status was available at 90 days for all children known to have neurocognitive deficit at day 28

Between 14th Feb 2017 and when enrolment ceased (28th Feb 2020) 1842 eligible children were enrolled into the COAST trial and included in all analyses (Fig. 1), Of 388 in the severe hypoxaemia stratum, 194 children were randomised to HFNT and 194 to LFO. Of 1454 children in the hypoxaemia stratum, 363 to were randomised to HFNT, 364 to LFO and 727 to permissive hypoxaemia. Baseline characteristics are presented in Table 1 demonstrating multiple features of severe pneumonia were present in all children. In the respective strata median SpO_2_ was 75 (68, 78) and 88 (85, 90); 75 and 65% had radiographically confirmed pneumonia. Additional baseline features and working diagnoses reported at 48 h are summarised in Supplemental Tables S2a, b.

**Table 1:** Characteristics of children at baseline by study stratum

	Severe hypoxaemia stratum (Saturations SpO_2_ < 80%)		Hypoxaemia stratum (Saturations SpO_2_ 80 to < 92%)		
Parameter	HFNT (*n* = 194)	Low-flow (*n* = 194)	HFNT (*n* = 363)	Low-flow (*n* = 364)	Permissive hypoxaemia (*n* = 727)
Median age, months (IQR)	7 (2–21)	7 (2–16)	9 (4–24)	9 (4–22)	10 (4–25)
Male sex *n* (%)	93 (47.9)	97 (50)	213 (58.7)	214 (58.8)	422 (58)
Median SpO_2_ (IQR)	75 (68–78)	75 (66–77)	88 (86–89)	88 (86–09)	88 (86–90)
SpO_2_ < 70% (severe hypoxaemia stratum) or < 85% (hypoxaemia stratum) *n* (%)	55 (28.4)	60 (30.9)	60 (16.5)	65 (17.9)	98 (13.5)
Median weight, kg (IQR)	6.8 (4.8–10)	6.6 (4.8–9)	8.1 (6.4–11)	7.9 (6.2–10.4)	8.3 (6.5–10.8)
Median MUAC, cm (IQR)	13 (11.4–14.2)	13 (11.5–14.2)	14 (13–15)	13.7 (12.7–14.7)	14 (12.8–15)
Fever (> 37.5 °C) *n* (%)	105 (54.1)	94 (48.5)	191 (52.6)	188 (51.6)	341 (46.9)
Hypothermia (< 36 °C)	5 (2.6)	13 (6.7)	4 (1.1)	9 (2.5)	20 (2.8)
Respiratory rate (IQR)	65 (56–79)	66.5 (56–79)	61 (52–69)	60 (52–68)	60 (51–67)
Tachypnoea *n* (%)	178 (91.8)	176 (90.7)	330 (90.9)	331 (90.9)	654/726 (90.1)
Indrawing *n*/*N* (%)	186/193 (96.4)	187/193 (96.9)	334 (92)	343 (94.2)	658 (90.5)
Cyanosis *n*/*N* (%)	13/192 (6.8)	15 (7.7)	3 (0.8)	3 (0.8)	4 (0.6)
Crepitations *n*/*N* (%)	136/192 (70.8)	149 (76.8)	271 (74.7)	267 (73.4)	530/725 (73.1)
Wheeze *n*/*N* (%)	42/191 (22)	37 (19.1)	91/362 (25.1)	93 (25.5)	182/723 (25.2)
Pneumonia signs on chest X-ray *n*/*N* (%)	128/165 (77.6)	113/155 (72.9)	227/347 (65.4)	217/342 (63.5)	426/695 (66.3)
Severe tachycardia *n*/*N* (%)	72 (37.1)	77 (39.7)	90/362 (24.9)	100 (27.5)	179 24.6)
Compensated shock *n*/*N* (%)	118/193 (61.1)	121 (62.4)	139 (38.3)	145 (39.8)	287 (39.5)
Severe pallor *n*/*N* (%)	31/193 (16.1)	24 (12.4)	36 (9.9)	26 (7.1)	57 (7.8)
Vomiting/diarrhoea *n*/*N* (%)	62/193 (32.1)	67 (34.5)	120/362 (33.1)	136 (37.4)	239/726 (32.9)
Dehydrated *n*/*N* (%)	11/191 (5.8)	20 (10.3)	11 (3)	15/363 (4.1)	22/725 (3)
Conscious level: responds to					
Pain or voice *n*/*N* (%)	33 (17)	24 (12.4)	14 (3.9)	14/363(3.9)	26 (3.6)
Unresponsive *n*/*N* (%)	8 (4.1)	13 (6.7)	2 (0.6)	3/363 (0.8)	3 (0.4)
Severely malnourished *n*/*N* (%)	19/193 (9.8)	29 (14.9)	10 (2.8)	24/362 (6.6)	33/726 (4.5)
Sickle cell disease *n*/*N* (%)	10 (5.2)	7 (3.6)	26 (7.2)	26 (7.1)	40 (5.5)
Developmental delay *n*/*N* (%)	16/193 (8.3)	16 (8.2)	21 (5.8)	17/362 (4.7)	40 (5.5)
Median haemoglobin, g/dl (IQR)	9.6 (7.3–11.1)	10.2 (8.7–11.3)	10.2 (8.8 –11.4)	10.3 (8.9–11.4)	10.4(8.9–11.7)
Severe anaemia (Hb < 5/dl) *n*/*N* (%)	24/184 (13)	13/182 (7.1)	33/352 (9.4)	26/348 (7.5)	59/698 (8.5)
White cell count (10 × 3/µL) median (IQR)	13.9 (9.5–20.3)	13.2 (9.4–18.7)	12.5 (9.2–17.3)	11.9 (8.3–16.4)	11.9 (8.3–17.9)
Leucocytosis (WBC > 11) *n*/*N* (%)	120/184 (65.2)	117/182 (64.3)	204/351 (58.1)	193/347 (55.6)	388/698 (55.6)
HIV^a^ *n* (%)	6/188 (3.2)	11/188 (5.9)	4/354 (1.1)	15/356 (4.2)	13/707 (1.8)
Malaria RDT^a^ *n*/*N* (%)	25/187 (13.4)	18/181 (9.9)	49/350 (14)	38/352 (10.8)	98/700 (14)
Malaria slide positive^a^ *n* (%)	11/187 (5.9)	13/182 (7.1)	26/354 (7.3)	15/354 (4.2)	36/700 (5.1)
Bacteraemia *n*/*N* (%)	10/187 (5.3)	7/183 (3.8)	8/354 (2.3)	8/353 (2.3)	19/705 (2.7)
Hypoglycaemia (glucose < 3/mmol/L) *n* (%)	10/192 (5.2)	9/193 (4.7)	7 (1.9)	5 (1.4)	21/727 (2.9)
Lactate > 5 mmol/L *n*/*N* (%)	41/191 (21.5)	38/190 (20)	34/354 (9.6)	21/358 (5.9)	54/715 (7.6)
Antibiotics in illness *n*/*N* (%)	112/186 (60.2)	121/192 (63)	205/358 (57.3)	201/358 (56.1)	404/721 (56)
Antimalarial in Illness *n*/*N* (%)	41/188 (21.8)	42/190 (22.1)	97/361 (26.9)	85/363 (23.4)	169/726 (23.3)

^a^*Missing* not valid, not done/recorded

### Adherence to randomisation

Most oxygen therapy was started within 30 min of screening except for three children on HFNT who died before this timepoint. Adherence to the randomisation strategy was excellent (Table 2). Per protocol LFO was started in the permissive hypoxaemia arm in 107/726 (14.7%) for drops of SpO_2_ < 80% and in 2 whose SpO_2_ was > 80%. Three children unable to tolerate HFNT were switched to LFO.

**Table 2:** Respiratory support and oxygen use per randomised strategy

	Severe hypoxaemia stratum (Saturations < 80%)		Hypoxaemia stratum (Saturations 80 to < 92%)		
	HFNT	Low flow	HFNT	Low-flow	Permissive hypoxaemia
Number of participants	194	194	363	364	727
Initiated allocated treatment^a^, *n*/*N* (%)	192/194 (99)	194/194 (100)	362/363 (99.7)	362/364 (99.5)	726/727 (99.9)
Protocol deviation^b^	0	0	1	2	2
Received oxygen (FiO_2_ > 21%) ever, *n*/*N* (%)	182/192 (93.8)	194/194 (100)	198/362 (54.7)	364/364 (100)	109/726 (15)
Interruptions with treatment strategy^c^, *n*/*N* (%)	3/192 (1.6)	2/194 (1)	9/362 (2.5)	7/362 (1.9)	
Starting flow rate l/min, median (IQR)	14 (10, 20)	1 (1, 2)	16 (13, 22)	1 (1, 2)	
Max flow rate, median (IQR)	14 (10, 20)	2 (1.5, 3)	16 (13, 22)	1.5 (1, 2)	
Initiated treatment with FiO_2_ > 21%, *n*/*N* (%)	49/192 (25.5)	194/194 (100)	3/362 (0.8)	363/364 (99.7%)	
**In the first 48 h**					
Hours of respiratory support^d^, mean (sd)	30.4 (18.6)	28 (18.8)	17 (17.2)	15.9 (16.6)	3.5 (10.6)
Hours of respiratory support^d^, median (IQR)	36.6 (9, 48)	32.1 (7.4, 47.7)	8.4 (2.8, 26.8)	6.8 (2.5, 25.3)	0 (0, 0)
Hours receiving additional oxygen^d^, mean (sd)	28.1 (18)	28 (18.8)	9.8 (14.6)	15.9 (16.6)	3.5 (10.6)
Hours receiving additional oxygen^d^, median (IQR)	33.1 (8.3–46.7)	32.1 (7.4, 47.7)	1 (0, 18.7)	6.8 (2.5, 25.3)	0 (0, 0)
Litres of oxygen used^d^, mean (sd)	2731 (2733)	3591 (4128)	969 (1890)	1481 (2480)	359 (1273)
Litres of oxygen used^d^, median (IQR)	1983 (502, 3571)	2743 (895, 4884)	113 (0, 1454)	480 (236, 2132)	0 (0, 0)
Any dose escalation *n* (%)	174 (89.7)	165 (85.1)	222 (60.2)	178 (48.9)	109 (15)

This table reports hours of treatment (and oxygen usage) for all children randomised to the specific strategies

^a^Two patients in the severe hypoxaemia stratum and one patient in the hypoxaemia stratum died before they started HFNT, one patient in permissive hypoxaemia arm absconded numbers initiated are revised according

^b^Protocol deviation The hypoxaemia stratum: HFNT: one patient switched to low flow before 48 h; low flow: two patients started on HFNT; permissive hypoxaemia: two initiated low-flow oxygen at SpO_2_ ≥ 80%

^c^Interruptions in oxygen treatment strategy: high-flow (both strata): power cuts (*n* = 2), child unable to tolerate (*n* = 4), nasal/facial trauma (*n* = 1) and child on nebulisation with > 15 min off O_2_ therapy (*n* = 5). Low-flow (both strata): child unable to tolerate (*n* = 1), child on nebulisation with > 15 min off O_2_ therapy (*n* = 7), 1 child not specified

^d^Hours of support and litres of O_2_ are summarised over all patients, those never receiving support/oxygen are assigned values of 0

### Use of oxygen and respiratory support

**Fig. 2:**
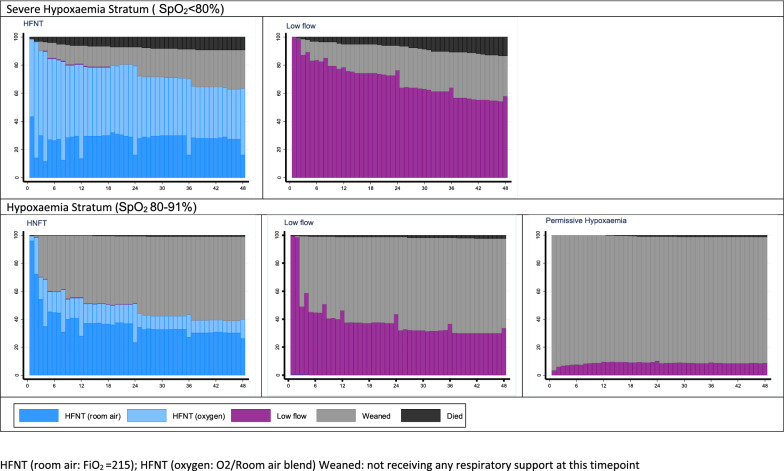
Proportion of children in each stratum receiving oxygen/respiratory support by group over 48- hours post-randomisation

In the severe hypoxaemia stratum, median duration (interquartile range, IQR) on respiratory support by HFNT was longer than for LFO 36.6 h (9, 48) versus 32.1 h (7.4, 47.7). In the hypoxaemia stratum duration of support by HFNT and LFO were similar (8.4 (2.8, 26.8) and 6.8 (2.5, 25.3) hours respectively (Fig. 2; Tables 2 and Supplemental T2). In the hypoxaemia stratum only 198/362 (54.7%) on HFNT had oxygen titrated in. In the severe hypoxaemia stratum although the number of hours of supplemental air/oxygen blend was similar over the 48 h period, the mean (standard deviation) volume of oxygen (litres) used was lower in HFNT: 2731L/child (2733) than LFO: 3591L/child (4128). In the hypoxaemia stratum both the time receiving supplemental air/oxygen [9.8 (14.6) versus 15.9 (16.6)] hours and oxygen volume received [969L/child (1890) versus 1481L/child (2480)] were considerably lower in HFNT than LFO. The permissive hypoxaemia strategy had lower hours/volume of oxygen therapy than the liberal strategies. Baseline SpO_2_ levels in children requiring oxygen in the permissive hypoxaemia arm were similar to those never receiving oxygen (Supplemental Figure S1).

### Mortality and neurocognitive sequelae

Vital status at 48 h (primary endpoint) was known in all children 388 (100%) in the severe hypoxaemia stratum and in 363 (100%), 363 (99.7%) and 724 (99.6%) in the hypoxaemia stratum children, respectively. By Day 28 vital status was known in 194 (100%) and 192 (99%) in the severe hypoxaemia stratum and in 362 (99.7%), 362 (99.7%) and 718 (99.2%) in the hypoxaemia stratum children respectively (Supplemental Figure S2). By 48 h in the severe hypoxaemia stratum, 18 (9.3%) in HFNT versus 26 (13.4%) in LFO groups had died. In the hypoxaemia stratum, 4 (1.1%), 9 (2.5%) and 10 (1.4%) children in HFNT vs LFO vs permissive hypoxaemia had died. The aOR for liberal versus permissive hypoxaemia was 1.16 (95% CI 0.49, 2.74) *p* = 0.728 and for HTNT versus LFO was 0.60 (0.33, 1.06) *p* = 0.076 (Table 3). By 28 days, 36 (18.6%) and 45 (23.4%) children in the severe hypoxaemia stratum and 12(3.3%), 15(4.1%) and 28(3.9%) in the hypoxaemia stratum had died [aOR for LFO versus permissive hypoxaemia 0.92 (0.53, 1.59); aOR for HFNT versus LFO was 0.75 (0.49, 1.16)]. Most neurocognitive sequelae primarily reported at Day 28 in survivors were transient, and all had resolved by Day 90. Details on blind ERC review of deaths and relationship are given in Table 3. Overall, there was one event identified by this process that was possibly related to treatment arm; most deaths appeared to be attributable to the severity of the underlying condition or comorbidity.

**Table 3:** Primary and secondary endpoints

	Severe hypoxaemia stratum (Saturations < 80%)		Hypoxaemia stratum (Saturations 80 to < 92%)			Adjusted odds ratio (95% CI)^a^	
	HFNT	Low-flow	HFNT	Low-flow	Permissive hypoxaemia	HFNT vs. low flow	Liberal^b^ vs. permissive hypoxaemia
**At 48 hours**							
Death (primary outcome) *n*/*N* (%)	18/194 (9.3)	26/194 (13.4)	4/363 (1.1)	9/363 (2.5)	10/724 (1.4)	0.60 (0.33, 1.06) (*p* = 0.076)**	1.16 (0.49, 2.74) (*p* = 0.728)
Treatment failure	15/175 (8.6)	18/167 (10.8)	5/359 (1.4)	8/353 (2.3)	33/711 (4.6)	0.75 (0.4, 1.41)	0.37 (0.19, 0.71)
Treatment failure or death	33/193 (17.1)	44/193 (22.8)	9/363 (2.5)	17/362 (4.7)	43/721 (6)	0.64 (0.41, 0.99)	0.55 (0.33, 0.91)
**At 28 days**							
Death *n*/*N* (%)	36/194 (18.6)	45/192 (23.4)	12/362 (3.3)	15/362 (4.1)	28/718 (3.9)	0.75 (0.49, 1.16)	0.92 (0.53, 1.59)
Hospital readmission	2/158 (1.3)	2/147 (1.4)	7/350 (2)	5/347 (1.4)	21/691 (3)	1.26 (0.46, 3.42)	0.56 (0.27, 1.15)
Neurocognitive sequelae	6/158 (3.8)	8/147 (5.4)	9/350(2.6)	11/346 (3.2)	16/689 (2.3)	0.74 (0.37, 1.48)	1.23 (0.63, 2.4)
Death or neurocognitive sequelae	42/194 (21.6)	53/192 (27.6)	21/362 (5.8)	26/361 (7.2)	44/717 (6.1)	0.74 (0.51, 1.08)	1.04 (0.67, 1.59)
						**Adjusted difference in means**^**a**^** (95% CI)**	
Weight for age z score, mean (s.d.) [*N*]	− 0.4 (1.8) [141]	− 0.6 (1.8) [136]	− 0 (1.7) [307]	− 0.2 (1.6) [311]	− 0.2 (1.6) [633]	0.05 (− 0.05, 0.16)	0.04 (− 0.05, 0.13)
Mid-upper arm circumference z score, mean (s.d.) [*N*]	− 0.8 (1.3) [93]	− 0.7 (1.4) [94]	− 0.1 (1.4) [251]	− 0.3 (1.3) [257]	− 0.2 (1.3) [528]	0.08 (− 0.11, 0.27)	0.02 (− 0.14, 0.17)
Length of stay mean (s.d.) [*N*]	6.3 (5.6) [194]	6.2 (6.1) [194]	5.3 (10.4) [363]	4.9 (3.7) [364]	4.5 (2.9) [726]	0.26 (− 0.43, 0.94)	0.62 (0.02, 1.22)
**Serious adverse event**							
At least one event no. of patients (%)	42/194 (21.6)	48/194 (24.7)	26/363 (7.2)	26/364 (7.1)	52/727 (7.2)		
Number of events	80	88	49	51	85		
**Endpoint Review Committee adjudication fatal events relationship to supplemental oxygen**^**b**^							
Not reviewed			2	1	3		
Unlikely/unrelated			14	13	26		
Insufficient information			0	1	0		
**Endpoint Review Committee adjudication fatal events relationship to delivery method**							
Not reviewed	3	6	2	0	1		
Unlikely/unrelated	36	39	14	15	27		
Possibly related	0	0	0	0	1		

~ Treatment failure defined as SpO_2_ < 92% in the presence of respiratory distress at 48 h post-randomisation

^a^Adjusted for initial SpO_2_ level (categorised as < 80, 80–84, 85–89, 90–91%) and centre (as a random effect), using a GLM

^b^In this comparison liberal strategies include HFNT and LFO (versus permissive hypoxaemia)

^**^Unadjusted OR high flow vs. low flow 0.61 (95% CI 0.36, 1.06), liberal oxygenation vs. permissive hypoxaemia 1.30 (95% CI 0.57, 2.99)

### Other clinical outcomes

Treatment failure to 48 h (defined as SpO_2_ < 92% plus respiratory distress) was somewhat lower in HFNT versus LFO aOR 0.75 (0.40, 1.41) and lower for liberal versus permissive hypoxaemia strategy aOR 0.37 (0.19, 0.71) (Table 3; Supplemental Figure S3). Further exploration of these treatment failures by severity of hypoxaemia (< 80, 80–89 and 90–91%) and comorbidities are reported in Supplemental Table S3. Overall, in the severe hypoxaemia stratum, 14/33 (42.4%) and 12/33 (36%) treatment failures were for saturations of < 80 and 80–89%, respectively. Day-28 hospital readmissions were low (≤ 3%) across all groups/strata (Supplemental Table S4). There was no evidence of a difference in mean hospital stay in the HFNT versus LFO [difference in means 0.26 (95% CI − 0.43, 0.94)] or liberal versus permissive hypoxaemia [0.62 (0.02, 1.22)] groups nor differences in anthropometric status at Day 28 (Table 3).

## Discussion

The premature termination of the COAST trial meant that it was unable answer the two specific hypotheses it was designed to address. First, whether liberal oxygenation therapy for children with hypoxaemia (SpO_2_ 80–91%) is superior to permissive hypoxaemia. In this stratum, however, the overall 48 h and Day 28 mortality was low across all arms including the permissive hypoxaemia arm. Second, as the trial was stopped prematurely there were insufficient data to demonstrate that HFNT was superior to LFO, but caution that the interpretation may be subject to a Type II error i.e. false negative (no benefit of HFNT versus LFO) since it was underpowered to uncover true effects, should they exist. However, the size of reduction (40%) in 48-h mortality warrants further investigation particularly for children with severe hypoxaemia (SpO_2_ < 80%). Notably, across both strata the method of HFNT administration was also relatively oxygen-sparing, using room air/oxygen blends without any evidence of harm. In the hypoxaemia stratum in many cases HFNT was provided on room air, leading to correction of hypoxaemia, without any evidence of harm.

Although the trial does not provide definitive data to inform treatment guidelines, the knowledge acquired from the trial adds a new level of uncertainty to the liberal use of oxygen as a supportive therapy [21]. Equipoise, centred on the degree of uncertainty about the relative benefits (or risks) of a clinical intervention, requires a clear distinction between pre-existing knowledge (evidence) and opinion or personal preference [22]. In the case of severe pneumonia, pre-existing evidence demonstrating clear benefits of oxygen therapy was poor [3]. On that basis the trial was both ethically and scientifically sound [23]. Substantial uncertainty behoves clinicians to conduct RCTs so that in future societies will know which treatments are better and ensuring patients will not be exposed to inferior or harmful treatments.

Whilst oxygen, as a supportive therapy, has been considered the standard treatment for pneumonia for a large part of the last century, the recognition of potential oxygen toxicity is relatively recent [24]. In several areas of emergency care and resuscitation, the use of oxygen (and other therapies included in guidelines [25]) are now being challenged by emerging evidence from clinical trials, including in paediatric populations [26, 27], and in systematic reviews [21, 28]. In neonates, medical oxygen used during resuscitation increases mortality, myocardial injury and renal injury [29].

COAST was designed with cognisance of the significant gaps between supply and demand for oxygen in hospitals in developing countries [11]. Sustainable provision of bottled oxygen is expensive and logistically challenging [30]; therefore, the WHO-preferred source of oxygen is oxygen concentrators [2]. Nevertheless, technical reports on the operational quality, availability and reliability of cylinders and oxygen concentrators indicate that, even when available, these are often faulty and unsustainable due to high cost [10], or depend upon erratic electricity supply [11]. With regard to demand on health services, the routine use of pulse oximetry to target oxygen therapy is poorly implemented despite having been recommended by WHO for triage of sick children for over two decades [5]. Hence, oxygen therapy is generally targeted by non-specific clinical signs that predict hypoxaemia poorly [4], thus exposing large numbers of children without hypoxaemia to oxygen therapy, and raising questions over safety [28, 31], cost and demand for a constrained health resource [32]. Our findings indicating that hypoxaemia in children with severe pneumonia can be corrected without additional oxygen (including those on HFNT using room air alone) is timely given the major demand on health services for oxygen therapy as a result of the COVID-19 epidemic.

Recognising the resource limitations and costs of oxygen therapy, the protocol incorporated relatively oxygen-sparing strategies in the investigational groups (HFNT and permissive hypoxaemia) and vital sign monitoring to ensure early weaning in both liberal (HFNT and LFO) strategies (Supplemental Figure S4). Thus, over 48 h even in the most liberal of the strategies (LFO) the median volume of oxygen in the respective strata (3279 and 1337 L/child) were substantially lower than in a LFO equivalent strategy (5990L over a median of 2.5 days) reported in the multicentre study in Nigeria investigating oxygen use in children with pneumonia [6].

One key limitation of the COAST trial was its premature termination. Nevertheless, the COAST trial represents the largest trial of oxygen therapy ever conducted in children and the only multicentre controlled trial. Another limitation was that we were unable to confirm the pulse oximetry readings with arterial blood gases. We chose BITMOS sat 801 + , since it incorporates Masimo Signal Extraction Technology^®^ and was the instrument of choice in the large multicentre pneumonia aetiology study [19]. The substantially lower mortality in both strata of the trial, contrast to the mortality rates of 9–10 and 26–30%, respectively, reported in previous studies in African children, on which our power calculation was based [4, 6, 7, 33]. Most were conducted prior to the introduction of vaccine*s* against the lead bacterial causes of paediatric pneumonia (*Haemophilus influenzae* type b and S*treptococcus pneumoniae*) into national immunization programs (including Uganda and Kenya) and/or the scaling-up of prevention and anti-retroviral medications for HIV. The resultant change in the aetiologic profile of childhood pneumonia was recently demonstrated by a multi-country case-control hypoxaemia studies of HIV-negative children with radiologically-confirmed pneumonia. Only 56/1749 (3·2%) cases had a positive blood culture and viruses accounted for 61% of cases. Respiratory syncytial virus, which is associated with low mortality [34], was the commonest pathogen [19]. The WHO severe pneumonia definition maximizes sensitivity over specificity, resulting in substantial overlap with other medical conditions [4, 35]. Notable in the COAST trial was the large proportion of previously undiagnosed cardiac conditions particularly in the sub-group with treatment failure. Nevertheless, radiologically confirmed pneumonia was present in a very large proportion of our study population, thus generalisable to children hospitalised with acute pneumonia in resource-limited hospitals.

HFNT has been shown in other populations to reduce the need for mechanical ventilation [36]. In that vast majority of hospitals in Africa access to mechanical ventilation or specialist intensive care is not standard. We, therefore, proposed that HFNT was a feasible alternative source of respiratory support owing to its relative simplicity in implementation, humidification, low risk of nosocomial infection [37]. In addition, the ability to blend both oxygen and room air to deliver positive end-expiratory pressure, thus, limiting exposure to high concentrations of oxygen (and potential toxicity) and with the prospect of reducing costs to health services. Bubble continuous positive airway pressure (bCPAP) is an alternative means of providing respiratory support. However, a recent trial in Malawian children showing worse outcomes in children receiving bCPAP than usual care advocates caution regarding implementation of bCPAP in a real-world setting without physician oversight [9].

In conclusion, our findings support the need for future trials with similar designs, particularly in settings where access to oxygen and/or mechanical respiratory support are restricted. The scale of the mortality reduction of HFNT over LFO, particularly in severely hypoxaemic children (40%) warrants further investigation. Oxygen-sparing strategies potentially offer cost-effective approaches to reducing overall oxygen requirements in overburdened health services and adds to the general findings in critical care than ‘less is more’ [38].

## Supplementary Information

Below is the link to the electronic supplementary material.

Supplementary file1 (DOCX 4576 KB)

Supplementary file1 (PDF 1539 KB)

Supplementary file1 (PDF 484 KB)

Supplementary file1 (PDF 159 KB)

## Data Availability

The datasets used and/or analysed during the current study are available from the corresponding author on reasonable request.
