# Supplementary material for: Randomised controlled trial of oxygen therapy and high-flow nasal therapy in African children with pneumonia
Source: Intensive Care Med. 2021 May 5;47(5):566–76. doi: 10.1007/s00134-021-06385-3 (PMC8098782; doi:10.1007/s00134-021-06385-3)
Supplement: Supplementary file 1 — Supplementary file1 (DOCX 4576 KB) [file 134_2021_6385_MOESM1_ESM.docx]

# CONTENTS

Table of Contents

[CONTENTS 1](#_Toc66015821)

[LIST OF INVESTIGATORS 2](#_Toc66015822)

[SUPPLEMENTARY METHODS 3](#_Toc66015823)

[(1) Trial sites 3](#_Toc66015824)

[(2) Inclusion/exclusion criteria from main trial protocol 3](#_Toc66015825)

[(3) Further details of allocation concealment 4](#_Toc66015826)

[(4) Further details pulse oximetry screening and oxygen delivery 5](#_Toc66015827)

[**Low flow oxygen** 5](#_Toc66015828)

[**High Flow Nasal Therapy (HFNT)** 5](#_Toc66015829)

[**Circuit Type and Interface** 6](#_Toc66015830)

[**Control (permissive hypoxaemia)** 8](#_Toc66015831)

[(5) Operational issues: Oxygen and electricity supplies 9](#_Toc66015832)

[(6) Severe Adverse Events reporting, endpoint ascertainment and adjudication 9](#_Toc66015833)

[**Expected adverse events** 9](#_Toc66015834)

[**Grading of Events**: 9](#_Toc66015835)

[**Causality** 10](#_Toc66015836)

[**Endpoint Review Committee** 10](#_Toc66015837)

[(7) Sample size calculation from the trial protocol and justification for a fractional factorial design 11](#_Toc66015838)

[(8) Statistical methods 11](#_Toc66015839)

[(9) Protocol Versions 12](#_Toc66015840)

[(10) Trial governance 14](#_Toc66015841)

[**a)** **Date Monitoring Committee (DMC) Interim analysis and reports** 14](#_Toc66015842)

[**b)** **TSC decision to stop the trial for feasibility** 15](#_Toc66015843)

[SUPPLEMENTARY RESULTS 17](#_Toc66015844)

[TABLES 17](#_Toc66015845)

[**Table S1a** **Additional Baselines and Working Diagnosis** 17](#_Toc66015846)

[**Table S1b Additional baseline Laboratory data** 18](#_Toc66015847)

[**Table S2 Respiratory Support and oxygen use for those receiving oxygen/respiratory support** 21](#_Toc66015848)

[**Table S3 Treatment Failures by Tertile of Oxygen Saturation – Final diagnosis** 22](#_Toc66015849)

[**Table S4 Reasons for readmissions** 24](#_Toc66015850)

[FIGURES 26](#_Toc66015851)

[**Figure S1 Baseline SpO_2_ levels in children requiring oxygen and never requiring oxygen: control arm** 26](#_Toc66015852)

[**Figure S2** **Kaplan Meier Survival to Day 28 by Stratum** 27](#_Toc66015853)

[**Figure S3 Correction of Oxygen saturations over 48 hours** 29](#_Toc66015854)

[**Figure S4** **Total volume of oxygen used (litres) over 48 hours in HFNT and LFO groups** 30](#_Toc66015855)

[REFERENCES 31](#_Toc66015856)

[APPENDIX 32](#_Toc66015857)

[STATISTICAL ANALYSIS PLAN- PDF 32](#_Toc66015858)

# LIST OF INVESTIGATORS

**Acknowledgements**

We thank all the participants and staff from all the centres participating in the COAST trial. This paper is published with permission from the Director of KEMRI.

**The COAST trial group consists of**:

**Participating Centres**:

**Uganda**

**Department of Paediatrics, Mulago Hospital, Makerere University, Kampala, Uganda**: S Kiguli, R O Opoka, E Nabawanuka, C Atwiine, C Chandiru , I Mufumba, W Nansalire, R Adoo, O Namubiru, N Naddamba

**Mbale Regional Referral Hospital Mbale**, Uganda: P Olupot-Olupot, W Okiror, MI Asinde, T Sennyondo, G. Abong, M Byampola, R Adong, L Ochen, R Muhindo, G Masifa,  G Odong

**Soroti Regional Referral Hospital Mbale,** Uganda: F Alaroker, M Nakuya, B Adongo, W Tino, J Muhindo, M Sejja, D Amorut M Ariima, C Engoru, I Egiriat, M Itipe, MG Atim

**Jinja Regional Referral Hospital Jinja, Uganda:** R O Opoka**,** A Tagoola, S Namuganza,  E Tenywa**,** J Kirikumwino, A Miriam, A Lubikire, R Ssenyonga, C Apolot, L Namulondo, E Emaru, D Kyavula, M Nyamwijja.

**Kenya**

**Kilifi County Hospital, Kilifi, Kenya** M Hamaluba, M Bakari, E Oguda

**Coast Provincial General Hospital, Mombasa**, **Kenya**: V Bandika, L Mwalekwa,

**KEMRI Wellcome Trust Research Programme, Kilifi, Kenya** (coordinating centre for the trial, genetics and respiratory diagnostics groups): K Maitland, A Mpoya, H Mnjalla, C Mogaka, P Maitha, S Uyoga, TN Williams, J Nokes, C Sande.

**Imperial College London (Trial Sponsor):** K Maitland, TN Williams; A Turnbull; A Bush

**Intensive Care National Audit & Research Centre (ICNARC), London, UK** : K Rowan, D Harrison, K Thomas, D Wiley, P Mouncey

**Critical Care Research Group, Adult Intensive Care Services The Prince Charles Hospital, Queensland**, Australia: J Fraser

**Data Management Systems**: C. Mogaka, A. Ali, (Kenya) G Abongo (Uganda)

**Independent COAST Trial Monitors**: S Nakalanzi, E Kivaya

**Endpoint Review Committee**: A. Turnbull, A. Odit, K. Maitland

**Trial Steering Committee**: E Molyneux (Chair), I Lubega, W. Macharia, J Crawley, M Peters

**Data Monitoring Committee**: T Peto (Chair), P Musoke, F Were, C Semple, J Todd (statistician).

# SUPPLEMENTARY METHODS

## (1) Trial sites

Six hospitals in two countries participated

- Kenya: KEMRI Wellcome Trust Programme, Kilifi County Hospital (KCH). The clinical programme at KCH is a well-established research centre on the coast of Kenya. It has been the site of several clinical studies on severe malaria including two large phase III trials (AQUAMAT^1^ and FEAST^2^). Coast Provincial General Hospital (CPGH) is based in the second largest city in Kenya (Mombasa) and has conducted in collaboration with KWTRP a number of research studies and Phase III clinical trials in children admitted to CPGH . Kilifi town/environs are 30-310 m above sea level whilst Mombasa is 50 m above sea level
- Uganda: Mulago National Referral Hospital. Mbale Regional Referral Hospital and Soroti Regional Referral Hospital. Mulago, Mbale and Soroti hospitals have large annual admissions to the paediatric ward. There has been considerable research capacity development in the last five years, with these three sites involved in the FEAST and TRACT trials^3,4^. Jinja Regional Referral Hospital has been involved in other Phase II and III trials and is based in a town at the start of the River Nile. Kampala is on average 1200 m above sea level, Jinja at 1204, Mbale at 1156 and Soroti 1080 m above sea level.

The six centres in Africa represent a spectrum of intensity of malaria transmission, from perennial and high (in Mbale Soroti and Jinja) to seasonal and meso-endmic (in Kampala, Mombasa and Kilifi). Mulago and CPGH are located within large urban areas, whereas the populations that utilise Mbale, Soroti, Jinja and Kilifi are more typically rural; however Mbale has a few slum developments within the town.

**Trial site Closure**

In June 2017 the trial stopped enrolment in Mulago Hospital, Kampala. Recruitment at this site was not restarted since it was considered as a hospital that could provide mechanical ventilation to children with severe pneumonia. By this stage 175 children had been enrolled (30 (17%) into COAST A stratum and 145 (83%) into COAST B stratum. In Kenya closure across the country hospital due to a health workers strike delayed the start of COAST trial in CPGH, Mombasa to February 2018. Over the following 6 months few eligible children presented to the paediatric sevices and thus, on 21^st^ September 2018, the trial investigators in consultation with the TSC made a decision to stop enrolment and wind down the study team as only 15 children had been enrolled (2 to COAST A and 13 to COAST B).

## (2) Inclusion/exclusion criteria from main trial protocol

The full wording of inclusion/exclusion criteria from the main trial protocol is as follows:

INCLUSION CRITERIA

- Aged between 28 days to 12 years
- History of respiratory illness (cough, upper respiratory tract symptom or any respiratory symptoms, e.g. rapid breathing or increase work of breathing)
- Hypoxaemia (pulse oximetry reading of SaO_2_ <92% recorded in room air over 5 minutes)
- Plus *any one of the following* signs of severe pneumonia (from 2013 WHO clinical definitions for pneumonia ^5^):

1. Sign of respiratory distress (any one of):
   - - severe lower chest wall in-drawing
     - use of auxiliary muscles
     - head nodding
     - inability to feed because of respiratory problems
2. Suspected pneumonia
   - fast breathing:
     - age 2–11 months: ≥ 50/minute
     - age 1–5 years: ≥ 40/minute
     - age 5-12 years ≥ 30/minute
   - chest auscultation signs:
     - decreased breath sounds
     - bronchial breath sounds
     - crackles
     - abnormal vocal resonance (decreased over a pleural effusion or empyema, increased over lobar consolidation)
     - pleural rub
3. Signs of pneumonia with a general danger sign:

- inability to breastfeed or drink
- lethargy or unconscious
- convulsions

EXCLUSION CRITERIA

- Known uncorrected cyanotic heart disease
- Assent/consent refusal by parent/carer
- Previously recruited to COAST
- Already received oxygen for this episode of illness*

*Clarified in the manual of operations as receiving oxygen at another facility (hospital or clinic) or for **more than 3 hours at the study site.**

## (3) Further details of allocation concealment

The Trial Statistician at the ICNARC Clinical Trials Unit (CTU) prepared the randomization lists before the trial commenced and these were kept at the ICNARC CTU, London. Opaque and sealed randomisation envelopes were prepared at KEMRI Wellcome Trust Research Programme Clinical Trials Facility and these were sent to each site. One set for Stratum A (SaO_2_ <80%) and one set for Stratum B (SaO_2_ ≥80% and <92%). The envelopes for each site were numbered consecutively and opened in numerical order. These will contain details of the treatment arms once opened.

This system has worked well in the emergency care trials, i.e. FEAST^6^ and TRACT^3,4^ trials. To facilitate protocol adherence, a maximum per site will be agreed upon (for example up to 4 children per day) will be enrolled per site. This approach was very successful with respect to protocol adherence and the quality of data generated in the FEAST trial.

## (4) Further details pulse oximetry screening and oxygen delivery

When screening for trial eligibility, BITMOS sat 801+ oximeters were used at all centres. These were selected as they utilise the latest Masimo SET® (Signal Extraction Technology) and incorporate a full-size high resolution plethysmogram. Bitmos pulse oximeters, measure oxygen saturations accurately during motion and low peripheral perfusion. The recommended monitoring site were for children > 10kg: ring or middle finger of non-dominant hand. Or if 3 kg to <10kg: the great toe.

Oxygen saturations were recorded over 5 minutes with the child breathing room air. At the end of the 5 minutes, the value was noted and the child will be assigned to the appropriate trial stratum if eligible. Children with SpO_2_ ≥ 92% remained on standard management (no supplemental oxygen) but could be re-screened within 24 hours of admission and enrolled into the trial if they remained eligible.

### **Low flow oxygen**

The method delivery depended on local preference but generally included a short, nasal prong, catheter or mask. Infants started on a flow rate of 1 l/min and children >1 year commenced on 2 l/min O2. The flow rates were titrated up over the first 30min-1 hour against oxygen saturation (to achieve SpO_2_ ≥92%) to a maximum of 2l/min in infants and 4 L/min in children if using nasal cannula. If higher rates were required children/infants were switched to mask and oxygen gradually increased to 15 L/min depending on type of mask and titrating to response of saturation measurements.

Children were eligible for weaning after a minimum of 2 hours on oxygen therapy. Weaning from oxygen occurred over 30 mins if SpO_2_ ≥92% (confirmed by BITMOS sat 801+ oximeters). Children continued to be monitored and LFO could be restarted again if SpO_2_ fell to <92% before 48hours. There was not crossover the HFNT.

**High Flow Nasal Therapy (HFNT)**

The method delivery for HFNT was by AIRVO2 (Fisher and Paykel Healthcare, Auckland, New Zealand).The AIRVO2 device is a humidifier with integrated flow generator that delivers to spontaneous breathing patients high flow warmed and humidified air/oxygen blend, thus permitting it to deliver respiratory support on room air alone (FiO2 21% Full details of AIRVO are provided <https://resources.fphcare.com/content/airvo-2-user-manual-ui-185045495.pdf>


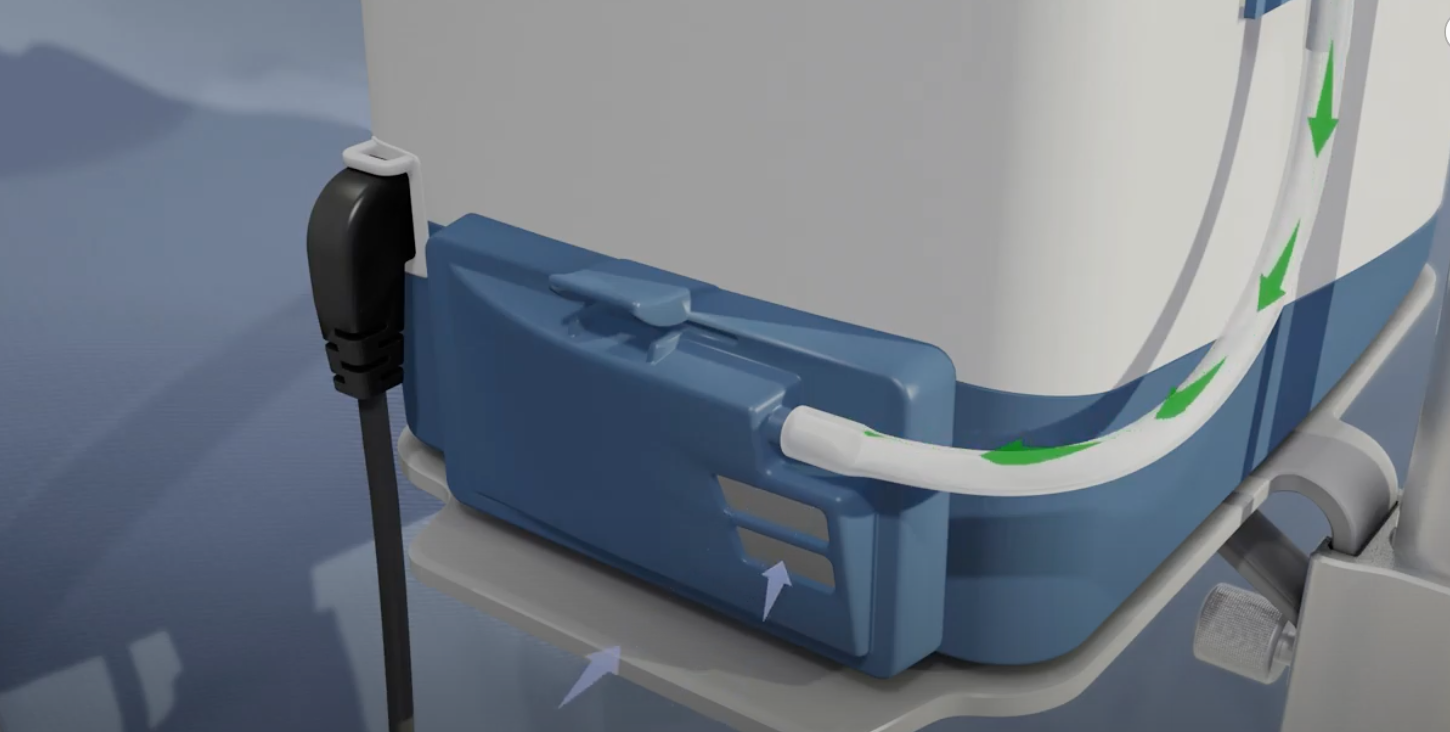


Room Air

Oxygen

**Circuit Type and Interface**

The appropriate patient interface, breathing circuit and OptiFlow settings were selected based on the manufactures’ guidance.

| **Weight** | **Circuit Type** | **Patient Interface** | **Optiflow setting** |
| --- | --- | --- | --- |
| 0 – 12 kgs | Paediatric | Infant (3-12kgs) or pediatric (8- 12kgs) if fit is better | Junior mode |
| > 12kgs | Adult | Small adult nasal cannula | Adult mode |

<https://www.fphcare.com/en-gb/products/optiflow-junior-breathing-circuit/>

<https://www.fphcare.com/en-gb/products/infant-nasal-cannula/>

**Flow rates**

AIRVO2 can generate flow rates of between 2L and 60L/min depending on the interface. The flow rates for children 3-12kg were 2l/kg per minute for those >12kg and flow rates were titrated to weight band (13-15kg: 30L/min; 16-30kg 35L/min). The first 15 mins of HFNT were in room air (21%); every 15mins for the first hour additional oxygen up to a maximum of 40% was titrated in to achieve SpO_2_≥92% (see tables below).

| **Time since start of treatment** | **O2 SATS** | **Current oxygen/flow setting** | **Action recommended** |
| --- | --- | --- | --- |
| **15 MINS** | <80% | HFA (FiO2 21%) | Add in Oxygen (Target FiO2 30%) |
|  | 80-91% | HFA (FiO2 21%) | Leave on HFA |
|  | ≥92% | HFA (FiO2 21%) | Leave on HFA |
| **30 MINS** | <80% | HFA (FiO2 21%)  HFA (FiO2 30%) | Add in Oxygen (Target FiO2 30%)  Add in Oxygen (Target FiO2 35%) |
|  | 80-91% | HFA (FiO2 21%)  HFA (FiO2 30%)  HFA (FiO2 35%) | Leave on current treatment. |
|  | ≥92% | HFA (FiO2 21%)  HFA (FiO2 30%)  HFA (FiO2 35%) | Leave on current treatment |
| **45 MINS** | <80% | HFA (FiO2 21%)  HFA (FiO2 30%)  HFA (FiO2 35%) | Add in Oxygen (Target FiO2 30%)  Add in Oxygen (Target FiO2 35%)  Leave on current treatment. |
|  | 80-91% | HFA (FiO2 21%)  HFA (FiO2 30%)  HFA (FiO2 35%) | Leave on current treatment. |
|  | ≥92% | HFA (FiO2 21%)  HFA (FiO2 30%)  HFA (FiO2 35%) | Leave on current treatment. |
| **1 HOUR** | <80% | HFA + FiO2 30% | Increase Oxygen Target FiO2 35% |
|  |  | HFA (FiO2 21%) | Add in oxygen Target FiO2 30% |
|  | 80-91% | HFA + FiO2 30% | Leave on current treatment |
|  |  | HFA (FiO2 21%) | Add in Oxygen Target FiO2 30% |
|  | ≥92% | HFA + FiO2 30% | Leave on current setting consider ‘Down-Titration’ protocol at 2hrs |
|  |  | HFA (FiO2 21%) | Leave on current setting consider ‘Weaning’ protocol at 2 hrs |

| **Time since start of treatment** | **O2 SATS** | **Current oxygen/flow setting** | **Action recommended** |
| --- | --- | --- | --- |
| **2 HOURS** | <80% | HFA + FiO2 35% | Increase Oxygen Target FiO2 40% (maximum therapy) |
|  |  | HFA + FiO2 30% | Increase Oxygen Target FiO2 35% |
|  |  | HFA (FiO2 21%) | Add in Oxygen Target FiO2 30% |
|  | 80-91% | HFA + FiO2 35% | Leave on current treatment |
|  |  | HFA + FiO2 30% | Increase Oxygen Target FiO2 35% |
|  |  | HFA (FiO2 21%) | Add in Oxygen Target FiO2 30% |
|  | ≥92% | HFA + FiO2 35% | Refer to ‘Down-Titration’ protocol |
|  |  | HFA + FiO2 30% | Refer to ‘Down-Titration’ protocol |
|  |  | HFA (FiO2 21%) | Refer to ‘Weaning’ protocol if also > 92% at one hour on HFA** |
| **4 HOURS**  **and beyond** | <80% | On High Flow Air (HFA) + FiO2 40% | Continue treatment |
|  |  | On HFA + FiO2 35% | Increase FiO2 to 40% |
|  |  | On HFA + FiO2 30% | Increase FiO2 to 35% |
|  |  | On HFA + FiO2 21% | Increase FiO2 to 30% |
|  |  | On no HFA treatment | Refer to ‘Initiation’ protocol |
|  | 80-91% | On High Flow Air (HFA) + FiO2 40% | Continue treatment |
|  |  | On HFA + FiO2 35% | Increase FiO2 to 40% |
|  |  | On HFA + FiO2 30% | Increase FiO2 to 35% |
|  |  | On HFA + FiO2 21% | Increase FiO2 to 30% |
|  |  | On no HFA treatment | Refer to ‘Initiation’ protocol |
|  | ≥92% | On High Flow Air (HFA) + FiO2 40% | Refer to ‘Down-Titration’ protocol |
|  |  | On HFA + FiO2 35% | Refer to ‘Down-Titration’ protocol |
|  |  | On HFA + FiO2 30% | Refer to ‘Down-Titration’ protocol |
|  |  | On HFA + FiO2 21% | Refer to ‘Weaning’ protocol |
|  |  | On no HFA treatment | Monitor at next time point |

**Two step Down- titration protocol**

| **Time from Start of Down Titration** | **O2 SATS** | **Action recommended** |
| --- | --- | --- |
| **0 MINS** | ≥92% | Reduce FiO2 by 10%  1/ If at 30% then reduce to room air (21%)  2/ If at 35% reduce to 25%  3/ If at 40% reduce to 30% |
| **15**  **MINS** | <80% | Return to the previous FiO2 and consider ‘Down-titration’ when stabilised |
|  | 80-91% | Leave on the current FiO2 setting |
|  | ≥92% | Turn off additional oxygen  (if already FiO2 21% then go to ‘Weaning High Flow’) |

| **Time** | **O2 SATS** | **CURRENT TREATMENT** | **ACTION** |
| --- | --- | --- | --- |
| **30 MINS** | <80% | HFA + additional oxygen | 1/ If Sats have worsened since 15 mins then go back to ‘General Titration’ protocol using increments recommended  2/ If the child is on FIO2 35% (trial of Maximum Therapy) |
|  |  | HFA (FiO2 21%) | Target FiO2 30% |
|  | 80-91% | HFA + FiO2 30% | Leave on the current FIO2 setting |
|  |  | HFA (FiO2 21%) | Add in Oxygen Target FiO2 30% |
|  | ≥92% | HFA + FiO2 30% | Consider ‘Down-Titration’ protocol again |
|  |  | HFA (FiO2 21%) | Consider ‘Weaning’ protocol |

Children eligible for weaning (minimum after 2 hours of therapy) were titrated down initially from oxygen to air alone then weaned of HFNT (flow rate initially halved then stopped). Children remained off oxygen/HFNT if SpO_2_ ≥92% (confirmed by BITMOS sat 801+ oximeters) but weaning would be discontinued or restarted again if SpO_2_ fell to <92% before 48hours. At 48 hours children still on HFNT requiring supplemental oxygen were switched onto LFO.

If children were not able to tolerate HFNT or developed a complication, the reason was recorded and per-protocol the child was switched to receive low flow oxygen therapy by mask/nasal cannula.

**Control (permissive hypoxaemia)**

Children were monitored in accordance to the protocol (extra observations were permitted) and if SpO_2_ (recorded over 5 minutes fell < 80%), per protocol oxygen therapy was initiated and the continued in the trial as per the ‘Low Flow Therapy’ protocol (as above).

**After 48 hours** oxygen therapy could be given as per local preference but was not recommended if child’s SpO_2_ remained ≥92%.

## (5) Operational issues: Oxygen and electricity supplies

Using the World Health Organization (WHO) Tool for Situational Analysis to Assess Emergency and Essential Surgical Care *Belle and colleagues* conducted a survey of oxygen supply and infrastructure in 231 health facilities across 12 African countries^7^. Of the 231 health centres surveyed only 99 (42.9%) had an uninterrupted source of oxygen whereas only 55 (23.8%) had a functioning oxygen concentrator. Uninterrupted electricity supplies (necessary for oxygen concentrators) was only available in 81 (35.1%) of health facilities. The same situation was present in a number of the hospitals involved in the trial which had issues for reliable clean power supplies and also dedicated sources of oxygen^8^.

For the COAST trial both oxygen cylinders and oxygen concentrators (AirSep Newlife Intensity10L and 5 L Oxygen concentrator, (with dual and single flow) were supplied to sites where oxygen was not always available. In order to ensure that children in the trial received their interventions, especially those dependent on electric supply centres were there were not hospital generators (with 24-hour power guaranteed) we supplied battery back-up and uninterrupted and stable power was supplied to the OptiFLow and oxygen concentrator through inverters. This ensured the ethical conduct of the trial.

## (6) Severe Adverse Events reporting, endpoint ascertainment and adjudication

Severe Adverse Events (SAEs) were defined following the International Committee for Harmonisation as events which led to death, were life-threatening, caused or prolonged hospitalization (excluding elective procedures), caused permanent disability, or were other medical conditions or with a real, not hypothetical risk of one of the previous categories.

All patients eligible for COAST are critically ill and due to the complexity of their condition are at increased risk of experiencing adverse events (AEs). Many of these events are expected because of the patient’s medical condition and standard treatment received in hospital, but may not be related to participation in the trial. Consequently, any unexpected AEs occurring because of the patient’s medical condition or standard hospital treatment were not reported. Pre-existing conditions did not qualify as AEs unless they worsened, and were documented in the patient’s medical notes.

### **Expected adverse events**

The following events were active solicited in participants up to 28 days following randomization:

- Nasal trauma
- Facial trauma
- Pneumothorax (life-threatening grade 4)
- Subcutaneous emphysema (life-threatening grade 4)
- Aspiration (life-threatening grade 4)

### **Grading of Events**:

- **Grade 1 Mild;** asymptomatic or mild symptoms; clinical or diagnostic observations only; intervention not indicated.
- **Grade 2 Moderate;** minimal, local or non-invasive intervention indicated; limiting age-appropriate instrumental activities of daily living.
- **Grade 3 Severe or medically significant** but not immediately life-threatening; hospitalization or prolongation of hospitalization indicated; disabling; limiting self-care activities of daily living.
- **Grade 4 Life-threatening consequences;** urgent intervention indicated.
- **Grade 5 Death** related to AE.

### **Causality**

The assignment of the causality should be made by the investigator responsible for the care of the participant using the definitions in the below:

| **Relationship** | **Description** |
| --- | --- |
| **Unrelated** | There is no evidence of any causal relationship |
| **Unlikely** | There is little evidence to suggest there is a causal relationship (e.g. the event did not occur within a reasonable time after administration of the trial medication). There is another reasonable explanation for the event (e.g. the participant’s clinical condition, other concomitant treatment). |
| **Possible** | There is some evidence to suggest a causal relationship (e.g. because the event occurs within a reasonable time after administration of the trial medication). However, the influence of other factors may have contributed to the event (e.g. the participant’s clinical condition, other concomitant treatments). |
| **Probable** | There is evidence to suggest a causal relationship and the influence of other factors is unlikely. |
| **Definitely** | There is clear evidence to suggest a causal relationship and other possible contributing factors can be ruled out. |
| **Not assessable** | There is insufficient or incomplete evidence to make a clinical judgement of the causal relationship. |

### **Endpoint Review Committee**

Deaths (including causes) were adjudicated by an Endpoint Review Committee (ERC) (two members that were not involved in trial enrolment/clinical) reviewed the SAE forms that had been blinded to the randomization arm and use of oxygen or respiratory support. The data that were available was all the bedside observations, baseline and ongoing clinical/laboratory data provided by the sites as well as the narratives of the attending clinician during the terminal event. In addition all new neurological sequelae that are documented at the 28 day and 6 month follow up visits. (In this context, “new” means either: a) not present at the time of randomisation, or b) deterioration of any pre-existing neurological problems) and any serious adverse events (SAEs) identified at independent unblinded review as being of concern.

The committee review blinded SAS and reported on underlying condition leading to death and the terminal clinical event and adjudicated it fatal event was associated with Mode of intervention (High vs Low Flow) and in COAST B whether treatment arm (oxygen therapy vs control) were thought to contribute to death. The likely cause of death was recorded and mode of death and causality.

## (7) Sample size calculation from the trial protocol and justification for a fractional factorial design

Sample size calculations were informed by identifying patients meeting the proposed COAST inclusion criteria within two datasets: the FEAST trial (n=873/3170; 28%)^6^; and the Kilifi District Hospital, in selected Kenya admission cohort (n=2609/36,621; 7%)^9^ – see below. Based on these data, it was estimated that two thirds of eligible children would present with SpO_2_ ≥80% (FEAST 63%; Kilifi 69%). Baseline 48-hour mortality for children receiving low flow oxygen was assumed to be 9% for children with SpO_2_ ≥80% (FEAST 10%; Kilifi 9%) and 26% with SpO_2_<80% (FEAST 30%; Kilifi 26%). We subdivided patients into two strata as part of the trial design since one of the trial interventions (permissive hypoxia) was only felt to be appropriate in children with baseline SpO2 of at least 80%, and yet the comparison of HFNT vs LFO remained relevant in those children with baseline SpO2 below 80%. Hence, patients with SpO2<80% were included and randomised to a restricted set of two out of the three treatment arms which were available to patients with SpO2>=80%. Due to the complex nature of the design, power calculations were undertaken by simulating datasets under the assumed alternative hypotheses and calculating the proportion of simulated datasets in which a significant effect (P<0.05) was detected for each of the two comparisons ^10^. Based on these simulations, a total sample size of 4,200 children would give 90% power to detect a clinically relevant difference of a 33% RR reduction associated with liberal oxygenation compared with permissive hypoxia, and a clinically relevant difference of a 25% RR reduction for high flow compared with low flow oxygen delivery. Mortality across 80-90% in Kilifi District Hospital Hospital with severe pneumonia signs..

| **Respiratory presentation* at admission** | **Mortality at 2 days** |
| --- | --- |
| Oxygen Saturations |  |
| 80-82% | 32/240 (13%) |
| 83-86% | 26/259 (10%) |
| 87-89% | 48/392 (12%) |
| Total | 106/891 (12%) |
| χ^2^ test | P=0.50 |

*Indrawing or deep breathing

The sample size calculation is based on the primary outcome of mortality at 48 hours with no losses to follow-up expected. Losses to follow-up are anticipated to increase to 2% at 28 days post-randomisation, based on data from the FEAST trial ^6^.

## (8) Statistical methods

The analyses are described in detail in a full Statistical Analysis Plan (Appendix).

The primary effect estimate for both comparisons included an adjustment for strata, and planned subgroup analyses were done to check for consistency of treatment effects across strata

Secondary analysis of the primary outcome included calculation of unadjusted odds ratios and a sensitivity analysis in which the primary analysis was repeated using different assumptions of mortality in patients with missing primary outcome.

Secondary outcomes were analysed using multilevel logistic models (treatment failure at 48 hours, neurocognitive sequalae in survivors, severe acute malnutrition, disability free survival, and hospital readmission at 28 days), multilevel linear models (anthropometric z-scores at 28 days, length of hospital stay, duration of respiratory support), with the same comparisons as for the primary endpoint. Survival at 28 days was estimated using Kaplan-Meier methods, and compared between treatment allocations using Cox regression, adjusting for starting Spo2 level and with shared frailty at site level.

## (9) Protocol Versions

**1^st^ Amendment**

**Version 1.0 dated 12/01/2016 to Version 2 dated 7^th^ July 2016**

1. Change of site from Kinshasa, DRC to Coast General District Hospital as it was not possible to conduct the COAST trial in the DRC. The local site investigator was added to the list of investigator Dr V Bandika.

**No recruitment had started in the trial**

**2^nd^ Amendment**

**Version 2.0 dated 7^th^ July 2016 to version 2.1 dated 11^th^ Jan 2017**

1. Change of the chair of the TSC to Prof. Elizabeth Molyneux
2. Amendments to the inclusion criteria clarifying that 'anyone of the following signs' indicated WHO specific signs for suspected severe pneumonia ·
3. Amendments to the exclusion criteria:

- Clarification that 'already received oxygen for this episode of illness' means at another hospital/health centre so we there is the addition of another facility to clarify the amendment.
- Addition in 'known chronic lung disease' (not including asthma), which was requested during national ethics approval in Uganda, since the trial aims to study severe acute pneumonia. The addition has been done but this will not affect recruitment because it is rare.

1. Re-naming Stratum 1 and Stratum 2 for the two groups in the trial to COAST A and COAST B
2. Addition of an additional extra clinical patient non-invasive bedside observations event (at 36 hours) to capture routinely clinical status at that point.
3. Removal of venous blood ‘gas’ tests at admission owing to the substantial inflated costs for the point of care diagnostics which could not be covered by the trial budget.
4. Malaria rapid diagnostic tests (RDT) to be done in addition to malaria blood slides
5. Clarification on the use of a modified Kilifi Developmental Milestone (KDM) checklist rather than the full KDM.
6. Removal of the Development B comfort score owing to its poor performance in the pilot and instead adopt the assessment of 'discomfort' for the child with the oxygen delivery/nasal cannulae and report the scores on an analogue scale.
7. Removal of the Pan-African Clinical Trial registry. The registration with ISRCTN was suffice.

**No recruitment had started in the trial**

**3^rd^ Amendment**

**2.1 dated 11^th^ Jan 2017 to 3.0 dated 14^th^ August 2017.**

1. Clarification, the initial protocol stated measurements were of SaO_2_ but this was a typo and this was corrected to SpO_2_.
2. Additional from European and Developing Countries Clinical Trials Partnership (EDCTP) (grant Number RIA2016S-1636; ISRCTN10829073) to incorporate additional objectives to the clinical trial in so that an integrated 'bundle of supportive care' would be tested.

This included

- 1. Randomization at 48-hours to receive Ready to Use Therapeutic Feed (in addition to their usual diet) for 56 days versus usual diet alone. Major outcome for this trial was change in mid-upper arm circumference (MUAC) at 90 days and/or as a composite with 90-day mortality.
  2. Additional long-term follow up to day 180 to estimate the longer-term effect of hospitalization with pneumonia involved two additional visits at day 90 and at 6 months (180 days) so that more detailed estimates on long term survival for all children in the trial can be provided.
  3. Additional sub-studies on anthropometric recovery and detailed assessment of pathogenic aetiology and biomarkers of pneumonia

1. Addition members of the study team included

**4^th^ Amendment**

**3.0 dated 14^th^ August 2017 to 3.1 dated 25^th^ April 2018.**

Change in PI from one site Dr Patricia Njuguna Position: Head of Clinical Trials Kilifi left to another post and was replaced by Dr Mainga Hamaluba.

**5^th^ Amendment**

**3.1 dated 25^th^ April 2018 to 3.2 dated 6^th^ Aug 2018**

- Adding ISRCTN number for COAST-Nutrition
- Adding in EDCTP as funder
- Change of JGHT Funder representative for MRC
- Section on Nutrition intervention (Section 7.4) details of RUTF and potential sources of their supply were added
- Patient information sheet and Consent for COAST Nutrition had not been added so were included in the appendices
- Inclusion of 2 potential additional sites in Uganda (Section 4): Jinja Regional Referral Hospital and Masaka Regional Referral Hospitals

## (10) Trial governance

In addition to the two planned interim analyses (per protocol) two additional reviews of accumulating safety (deaths and neurological sequelae) were conducted by the DMC. The letters summarising the four reviews are detailed in the section below

1. **Date Monitoring Committee (DMC) Interim analysis and reports**
2. **First interim analysis (27^th^ July 2017)**

“The data from 382 children recruited to 15 June 2017 were carefully reviewed by the DMC who, unanimously, had no concerns regarding safety issues. We were satisfied that without exception, all trial participants are being monitored closely, as per the Trial Protocol (version 2.1, 11 January 2017) and Manual of Operations, and that all SAEs are being reported in a timely manner. The strong recommendation of the DMC is that the trial should continue as planned”.

1. **Review for Safety (not interim analysis):**

The DMC reviewed data from 945 patients recruited up to 15 April 2018.

The DMC looked carefully at mortality and neurocognitive outcomes and SAEs and concluded that there were no safety concerns”.

The DMC recommend that the trial should continue as planned.

1. **Review for Safety (not interim analysis): 22^nd^ May 2019**

- The DMC met (by teleconference) and looked carefully at mortality, neurocognitive outcomes and SAEs and have no safety concerns.
- They would like congratulate the team on the high rate of follow up and protocol adherence.
- The DMC recommended the trial should continue without modification.
- They believe the trial hypothesis remains clinically relevant and they would like to encourage the team to make strenuous efforts to improve recruitment.

1. **Second interim analysis (27^th^ November 2019)**

The independent DMC met by teleconference on Wednesday 27 November 2019. The DMC reviewed a confidential report, including the second planned interim analysis of the trial based on data from 1645 children recruited to 25 October 2019 and all reports of Serious Adverse Events (SAEs) to date.

- There were no concerns over safety arising from the oxygenation or SAE data.
- Having reviewed the results of the interim analysis of mortality at 48 hours, the DMC recommend that the trial should continue as planned.
- In response to the specific concerns regarding power raised by the TSC, the DMC noted the

revised power calculations and confirmed these as correct. The DMC anticipate the planned

sample size of 4,200 will achieve acceptable power to provide clinically meaningful results for a number of secondary outcomes.

- In conclusion, the DMC were strongly supportive of the trial continuing to full recruitment as originally planned.

1. **TSC decision to stop the trial for feasibility**

The COAST trial aimed to enrol 4200 children with severe pneumonia in 5 centres over 30 months. The COAST trial began in February 2017 (after extensive preparation and training) enrolling only in Uganda (three centres) and later in Kilifi Kenya (May 2017). In June 2017 recruitment in Mulago Hospital, Kampala was halted owing to a concern raised that the trial was causing harm to children.

Subsequently there was a long campaign, largely led by a single individual in Uganda, succeeded in stopping the trial across all 3 study sites, on the grounds that it was endangering children’s lives by denying them oxygen. The individual had made unsubstantiated claims to a number of medical bodies in Uganda that the COAST trial was killing ‘many Uganda children’. Their view was the permissive hypoxaemia arm was unethical on the grounds that oxygen is recommended in guidelines, albeit on low quality of evidence. Independent investigations by a number of medical bodies found that the trial was being conducted to the highest standards and noted that the interim analyses reviewed by the independent Data Monitoring Committees indicated no evidence of harm. Ethics and regulatory committees supported this view. In addition, the National Paediatric Associations in Kenya and Uganda sent letters of endorsement.

During the 3^rd^ (June 2019) and 4th (November 2019) meetings of the Trial Steering Committee reviewed the report from the DMC and overall recruitment and timelines with respect to funding. Both meetings concluded that unless the trial teams could enrol ~ 90 patients/month (see Figure below) then the likelihood of completing the trial and obtaining additional funding (a costed extension was due for consideration in 2020) was uncertain. In November 2019 meeting the TSC indicated that if there were to be any more stoppages in the trial in Uganda they would consider recommend the study to stop enrolment to the trial.

**Figure Recruitment against anticipated target (June 2019)**


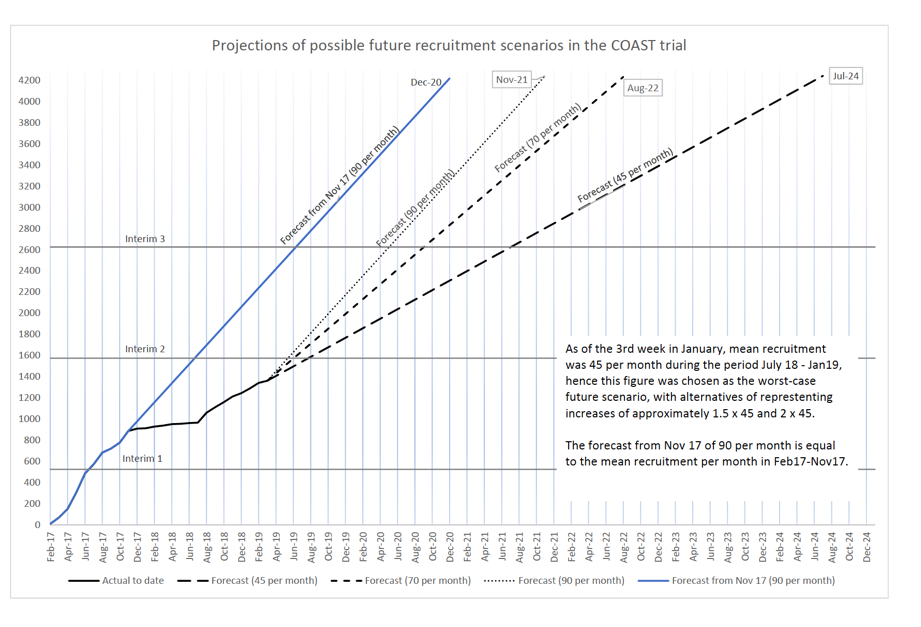


In January 2020 a civil society group, prompted by our accuser, petitioned to the high court in Uganda to have the trial stopped (for the 5^th^ time) in Uganda. That meant that in the 36 months from the start of the trial Uganda centres had only be able to enrol children for 21 months. This prompted an extemporaneous TSC consultation in February which resulted in the Chair of the TSC writing to the Chief Investigator and Sponsor (Imperial College) to recommend that the trial to be stopped on the grounds of feasibility. Justifying their decision that recruitment had been irregular and slow thus there was no merit in continuing enrolment given considerable future uncertainty. The TSC indicated that they had no reassurances that the trial would not continue to be halted in Uganda. The trial was halted in February 2020.

# SUPPLEMENTARY RESULTS

## TABLES

### **Table S1a** **Additional Baselines and Working Diagnosis**

| Parameter | High-flow (n=194) | Low-flow (n=194) | High-flow (n=363) | Low-flow (n=364) | Control  (n=727) |
| --- | --- | --- | --- | --- | --- |
| Abnormal Temp (>37.5 or <36C) n% | 110 (56.1) | 107 (55.2) | 195 (53.7) | 197 (54.1) | 361 (49.7) |
| Fever history in this illness n % | 176/193 (90.7) | 173 (89.2) | 326 (89.8) | 321 (88.2) | 648 (89.1) |
| Cough n % | 182/193 (93.8) | 184 (94.8) | 350 (96.4) | 354 (97.3) | 711 (97.8) |
| Deep Breathing n % | 68/193 (35.1) | 78 (40.2) | 114 (31.4) | 97 (26.6) | 167 (23.0) |
| Grunting n % | 62/193 (32.0) | 62/193 (32.0) | 53 (14.6) | 44 (12.1) | 91 (12.5) |
| Respiratory Severity Distress score |  |  |  |  |  |
| 0 n % | 53 (27.3) | 56/193 (28.9) | 210 (27.3) | 223 (61.5) | 447 (61.5) |
| 1-3 n % | 90 (46.4) | 88/193 (45.6) | 120 (33.1) | 123 (33.8) | 243 (33.4) |
| 4-6 n % | 47 (24.2) | 47 (24.3) | 24 (6.6) | 17 (4.6) | 36 (4.9) |
| 7-9 n % | 4 | 2 () | 0 | 1 | 1 |
| Bradycardia n % | 2 (1.0) | 1 (0.5) | 0/362 (0) | 2/363 (0.5) | 2 (0.3) |
| Decompensated Shock n % | 2/185 (1.0) | 1/186 (0.5) | 0/357 (0) | 1/357 (0.3) | 1/714 (0.1) |
| Altered consciousness n % | 35/191 (18.3) | 35/193 (18.0) | 27 (7.4) | 18/362 (4.9) | 43/726 (5.9) |
| Fits in the illness n % | 17/193 (9.3) | 18/194 (3.9) | 14/362 (6.0) | 22/362 (6.0) | 26/726 (3.6) |
| Neck Stiffness/Bulging Font n % | 0/193 (0) | 4/193 (3.0) | 0 (89.8) | 1/363 (0.5) | 1/725 (0.2) |
| Prior admission to another facility for > 24 hours | 21/193 (10.8) | 24 (12.4) | 23 (6.3) | 24 (6.6) | 54/726 (7.4) |
|  |  |  |  |  |  |
| **Past history N (%)** |  |  |  |  |  |
| Gestational Age < 37 weeks* | 186/193 (95.9) | 187/191 (96.4) | 334 (92.0) | 343 (94.2) | 658 (90.5) |
| HIV on ARV | 0 (0) | 4 (2.0) | 3 (0.8) | 7 (1.9) | 4/725 (0.5) |
| Tuberculosis | 1/191 (0.5) | 2/191 (1.0) | 1/362 (0.3) | 0 (0) | 4/724 (0.6) |
| Asthma | 2/193 (0.5) | 0/193 (10) | 4/362 (1.1) | 4 (1.1) | 16 (2.2) |
| >=2 hospital admission this year | 17/187 (8.8) | 21/193 (10.8) | 42/361 (11.6) | 47/258 (12.9) | 90/723 (12.4) |
| Known Epilepsy | 17/187 (8.8) | 21/191 (10.8) | 42/361 (1.6) | 47/358 (12.9) | 90/373 (12.4) |
| Number of acute diagnoses |  |  |  |  |  |
| 1 | 79 (40.7) | 94 (48.5) | 202 (55.6) | 191 (52.5) | 400 (55.0) |
| 2 | 14 (58.8) | 100 (51.5) | 161 (44.4) | 172 (47.3) | 327 (45.0) |
| Not recorded | 1 (0.5) | 0 (0) | 0 (0) | 1 (0.3) | 0 (0.) |
|  |  |  |  |  |  |
|  |  |  |  |  |  |
| **Working Diagnoses**  N(%) |  |  |  |  |  |
| LRTI - any (inc suspected TB) | 183 (94.3) | 186 (95.9) | 338 (93.1) | 345 (94.8) | 670 (92.2) |
| Severe malaria - all forms | 30 (15.5) | 24 (12.4.) | 58 (16.0) | 50 (13.7) | 142 (19.5) |
| Sepsis/septicaemia | 28 (14.4) | 23 (11.9) | 34 (9.4) | 34 (9.3) | 58 (8.0) |
| Severe anaemia | 24 (12.4) | 13 (6.7) | 37 (10.2) | 20 (5.5) | 65 (8.9) |
| Sickle cell disease | 10 (5.2) | 7 (3.6) | 26 (7.2) | 26 (7.1) | 40 (5.5) |
| Severe Malnutrition | 19 (9.8) | 23 (11.9) | 10 (2.8) | 16 (4.4) | 22 (3.0) |
| Asthma | 3 (1.5) | 2 (1.0) | 18 (5.0) | 15 (4.1) | 42 (5.8) |
| Gastroenteritis/diarrhoea | 13 (6.7) | 8 (4.1) | 14 (3.9) | 19 (5.2) | 26 (3.6) |
| URTI - any | 2 (1.0) | 6 (3.1) | 11 (3.0) | 12 (3.3) | 32 (4.4) |
| Developmental delay/cerebral palsy | 8 (4.1) | 12 (6.2) | 13 (3.6) | 10 (2.7) | 16 (2.2) |
| Heart condition: congenital/other | 10 (5.2) | 15 (7.7) | 9 (2.5) | 10 (2.7) | 10 (1.4) |
| Bronchiolitis, | 3 (2.1) | 3 (1.5) | 2 (0.6) | 8 (2.2) | 18 (2.5) |
| Measles | 4 (1.5) | 3 (0.6) | 2 (2.7) | 10 (2.7) | 13 (1.8) |
| HIV/AIDS | 5 (2.6) | 5 (2.6) | 4 (1.1) | 7 (1.9) | 5 (0.7) |
| Meningitis or encephalitis | 4 (2.1) | 3 (1.5) | 2 (0.6) | 2 (0.5) | 4 (0.6) |
| Other chest diagnosis | 2 (1.0) | 1 (0.5) | 2 (0.6) | 4 (1.1) | 4 (0.6) |

Bradycardia defined at (<70 beats/min if < 5 yrs or <80 beats/min if ≥5 years)

Altered consciousness based on the responsiveness (AVPU) scale: any child < A)

LRTI Lower Respiratory Tract Infection; URTI Upper Respiratory Tract Infection

* missing=not valid, not done not recorded

### **Table S1b Additional baseline Laboratory data**

|  |  | **COAST A: High-flow** |  | **COAST A: Low-flow** |  | **COAST B: High-flow** |  | **COAST B: Low-flow** |  | **COAST B: Permissive hypoxia** |  |
| --- | --- | --- | --- | --- | --- | --- | --- | --- | --- | --- | --- |
| Full blood count |  |  | % |  | % |  | % |  | % |  | % |
| Haemoglobin (g/dL) | Mean | 9.25 |  | 9.88 |  | 9.90 |  | 10.33 |  | 10.19 |  |
|  | s.d. | 2.74 |  | 2.52 |  | 2.665 |  | 7.05 |  | 3.94 |  |
|  | Median | 9.60 |  | 10.2 |  | 10.2 |  | 10.3 |  | 10.4 |  |
|  | LQ | 7.30 |  | 8.7 |  | 8.8 |  | 8.9 |  | 8.9 |  |
|  | UQ | 11.10 |  | 11.3 |  | 11.4 |  | 11.4 |  | 11.7 |  |
|  | N* | 184 |  | 182 |  | 352 |  | 348 |  | 698 |  |
| Severe anaemia (Hb<6g/dl), N(%) | No | 160 | 82.5 | 169 | 87.1 | 319 | 87.9 | 322 | 88.5 | 639 | 87.9 |
|  | Yes | 24 | 12.4 | 13 | 6.7 | 33 | 9.1 | 26 | 7.1 | 59 | 8.1 |
|  | NA | 10 | 5.2 | 12 | 6.2 | 11 | 3.0 | 16 | 4.4 | 29 | 4.0 |
| White cell count (10x3/uL) | Mean | 17.46 |  | 15.78 |  | 15.39 |  | 14.44 |  | 15.28 |  |
|  | s.d. | 14.06 |  | 10.10 |  | 12.40 |  | 11.22 |  | 11.65 |  |
|  | Median | 13.90 |  | 13.20 |  | 12.50 |  | 11.90 |  | 11.95 |  |
|  | LQ | 9.50 |  | 9.4 |  | 9.2 |  | 8.3 |  | 8.3 |  |
|  | UQ | 20.35 |  | 18.7 |  | 17.3 |  | 16.4 |  | 17.9 |  |
|  | N* | 184 |  | 182 |  | 351 |  | 347 |  | 698 |  |
| Leucocytosis (WBC>11), N(%) | No | 64 | 33.0 | 65 | 33.5 | 147 | 40.5 | 154 | 42.3 | 310 | 42.6 |
|  | Yes | 120 | 61.9 | 117 | 60.3 | 204 | 56.2 | 193 | 53.0 | 388 | 53.4 |
|  | NA | 10 | 5.2 | 12 | 6.2 | 12 | 3.3 | 17 | 4.7 | 29 | 4.0 |
| Lactate >= 5mmol/L, N(%) | No | 150 | 77.3 | 152 | 78.4 | 320 | 88.2 | 337 | 92.6 | 661 | 90.9 |
|  | Yes | 41 | 21.1 | 38 | 19.6 | 34 | 9.4 | 21 | 5.8 | 54 | 7.4 |
|  | NA | 3 | 1.5 | 4 | 2.1 | 9 | 2.5 | 6 | 1.6 | 12 | 1.7 |
| Glucose <3.0 mmol/L, N(%) | No | 182 | 93.8 | 184 | 94.8 | 356 | 98.1 | 359 | 98.6 | 705 | 97.0 |
|  | Yes | 10 | 5.2 | 9 | 4.6 | 7 | 1.9 | 5 | 1.4 | 21 | 2.9 |
|  | NA | 2 | 1.0 | 1 | 0.5 | 0 | 0.0 | 0 | 0.0 | 1 | 0.1 |
| Sodium (mmol/L) | Mean | 133.94 |  | 134.82 |  | 134.35 |  | 133.69 |  | 132.04 |  |
|  | s.d. | 8.35 |  | 5.45 |  | 5.65 |  | 5.82 |  | 12.59 |  |
|  | Median | 135.00 |  | 135 |  | 134.5 |  | 134 |  | 134 |  |
|  | LQ | 131.00 |  | 132 |  | 130 |  | 130 |  | 130 |  |
|  | UQ | 137 |  | 137 |  | 138 |  | 138 |  | 137 |  |
|  | N* | 35 |  | 34 |  | 40 |  | 39 |  | 73 |  |
| Potassium (mmol/L) | Mean | 4.34 |  | 4.59 |  | 4.20 |  | 4.26 |  | 4.37 |  |
|  | s.d. | 0.91 |  | 1.18 |  | 0.63 |  | 1.18 |  | 1.32 |  |
|  | Median | 4.10 |  | 4.3 |  | 4.25 |  | 4.2 |  | 4.2 |  |
|  | LQ | 3.70 |  | 3.8 |  | 3.7 |  | 3.7 |  | 3.8 |  |
|  | UQ | 4.80 |  | 4.9 |  | 4.6 |  | 4.5 |  | 4.7 |  |
|  | N* | 35 |  | 34 |  | 40 |  | 39 |  | 74 |  |
| Creatinine (umol/L) | Mean | 62.76 |  | 67.77 |  | 48.41 |  | 36.58 |  | 41.64 |  |
|  | s.d. | 33.93 |  | 53.61 |  | 33.60 |  | 19.10 |  | 22.19 |  |
|  | Median | 53.00 |  | 51.5 |  | 43 |  | 38 |  | 41 |  |
|  | LQ | 38.00 |  | 39 |  | 31.5 |  | 20.2 |  | 30 |  |
|  | UQ | 78.00 |  | 82 |  | 54.5 |  | 50 |  | 49 |  |
|  | N* | 35 |  | 34 |  | 40 |  | 39 |  | 73 |  |
| Urea (umol/L) | Mean | 5.43 |  | 6.76 |  | 4.52 |  | 3.27 |  | 3.59 |  |
|  | s.d. | 5.38 |  | 9.81 |  | 6.16 |  | 2.44 |  | 3.93 |  |
|  | Median | 3.85 |  | 3.6 |  | 2.5 |  | 2.7 |  | 2.45 |  |
|  | LQ | 2.10 |  | 2.3 |  | 1.8 |  | 1.8 |  | 1.8 |  |
|  | UQ | 6.00 |  | 5.8 |  | 4.6 |  | 3.9 |  | 3.8 |  |
|  | N* | 34 |  | 29 |  | 38 |  | 37 |  | 66 |  |
| Malaria RDT, N(%) | Positive | 25 | 12.9 | 18 | 9.3 | 49 | 13.5 | 38 | 10.4 | 98 | 13.5 |
|  | Negative | 162 | 83.5 | 163 | 84.0 | 301 | 82.9 | 314 | 86.3 | 602 | 82.8 |
|  | Invalid | 1 | 0.5 | 0 | 0.0 | 0 | 0.0 | 0 | 0.0 | 2 | 0.3 |
|  | Not done | 1 | 0.5 | 3 | 1.5 | 5 | 1.4 | 2 | 0.5 | 3 | 0.4 |
|  | NA | 5 | 2.6 | 10 | 5.2 | 8 | 2.2 | 10 | 2.7 | 22 | 3.0 |
| Malaria blood film, N(%) | Positive | 11 | 5.7 | 13 | 6.7 | 26 | 7.2 | 15 | 4.1 | 36 | 5.0 |
|  | Negative | 176 | 90.7 | 169 | 87.1 | 328 | 90.4 | 339 | 93.1 | 664 | 91.3 |
|  | Invalid | 0 | 0.0 | 0 | 0.0 | 0 | 0.0 | 0 | 0.0 | 2 | 0.3 |
|  | Not done | 1 | 0.5 | 1 | 0.5 | 1 | 0.3 | 0 | 0.0 | 3 | 0.4 |
|  | NA | 6 | 3.1 | 11 | 5.7 | 8 | 2.2 | 10 | 2.7 | 22 | 3.0 |
| Pathogen isolated, N(%) | No | 177 | 91.2 | 176 | 90.7 | 346 | 95.3 | 345 | 94.8 | 686 | 94.4 |
|  | Yes | 10 | 5.2 | 7 | 3.6 | 8 | 2.2 | 8 | 2.2 | 19 | 2.6 |
|  | NA | 7 | 3.6 | 11 | 5.7 | 9 | 2.5 | 11 | 3.0 | 22 | 3.0 |
| HIV point of care test, N(%) | Positive | 6 | 3.1 | 11 | 5.7 | 4 | 1.1 | 15 | 4.1 | 13 | 1.8 |
|  | Negative | 182 | 93.8 | 177 | 91.2 | 350 | 96.4 | 341 | 93.7 | 694 | 95.5 |
|  | Invalid | 2 | 1.0 | 2 | 1.0 | 3 | 0.8 | 1 | 0.3 | 1 | 0.1 |
|  | NA | 4 | 2.1 | 4 | 2.1 | 6 | 1.7 | 7 | 1.9 | 19 | 2.6 |

FBC Full blood count

LQ lower quartile

UQ Upper quartile

N* number non-missing NA not available/unknown

### **Table S2 Respiratory Support and oxygen use for those receiving oxygen/respiratory support**

|  | STRATUM A (Saturations < 80%)) | | STRATUM B (Saturations 80 to < 92%) | | |
| --- | --- | --- | --- | --- | --- |
|  | HFNT | Low-flow | HFNT | Low-flow | Control |
| Number of participants | 194 | 194 | 363 | 364 | 727 |
| In the first 48 hours | | | | | |
| Hours of respiratory support^d^, mean (sd) | 30.4 (18.6) | 28.0 (18.8) | 17.0 (17.2) | 15.9 (16.6) | 23.4 (16.8) |
| Hours of respiratory support^d^, median (IQR) | 36.6 (9.0, 48) | 32.1 (7.4, 47.7) | 8.4 (2.8, 26.8) | 6.8 (2.5, 25.3) | 24.0 (7.6,41.6) |
| Hours receiving additional oxygen^d^, mean (sd) | 28.1 (18.0) | 28.0 (18.8) | 17.9 (15.7) | 15.9 (16.6) | 23.4 (16.8) |
| Hours receiving additional oxygen^d^, median (IQR) | 33.1 (8.3 – 46.7) | 32.1 (7.4 – 47.7) | 15.7 (3.1 – 31.3) | 6.8 (2.5 – 25.3) | 24.0 (7.6 – 41.6) |
| Litres of oxygen used^d^, mean (sd) | 2731 (2733) | 3591 (4129) | 1784 (2265) | 1481 (2480) | 2393 (2446) |
| Litres of oxygen used^d^, median (IQR) | 2151 (775-3645) | 2743 (895-4884) | 1197 (325-2414) | 480 (236-2132) | 1530 (543-3330) |
| Any dose escalation n % | 174 (89.7) | 165 (85.1) | 222 (61.2) | 178 (48.9) | 109 (15.0) |
|  |  |  |  |  |  |

Note: this table reports hours of treatment: litres oxygen summarised for only in for those receiving active treatment

^a^  Two patients in Stratum A and 1 patient in Stratum B died before they started HFO,1 patient in Control arm absconded numbers initiated are revised according

^b^ Protocol Deviation Stratum B: HFNC: 1 patient switched to low-flow before 48 hours; Low-flow: 2 patients started on HFNC; Control: 2 initiated low flow oxygen at SpO2>=80%

^c^ Interruptions in oxygen treatment strategy: High Flow (Stratum A and B) power cuts (n=2), child unable to tolerate (n=4), nasal/facial trauma (n=1) and child on nebulization with >15 mins off O_2_ therapy (n=5). Low Flow (Stratum A and B): child unable to tolerate (n=1), child on nebulization with >15 mins off O_2_ therapy (n=7), 1 child not specified

^d^ hours of support are summarised over all patients receiving respiratory support, litres of oxygen are summarised over all patients receiving additional oxygen

### **Table S3 Treatment Failures by Tertile of Oxygen Saturation – Final diagnosis**

| Stratum/Arm | SpO2 at 48 hrs | Number | Patients with working diagnosis of: | N |
| --- | --- | --- | --- | --- |
| A - high flow | <=80% | 7 | Heart condition, congenital or other | 3 |
|  |  |  | HIV/AIDS | 1 |
|  |  |  | LRTI - any (includes suspected TB) | 7 |
|  |  |  | Other | 1 |
|  |  |  | Severe malaria - all forms | 1 |
|  | 81-89% | 4 | LRTI - any (includes suspected TB) | 4 |
|  |  |  | Malnourished | 2 |
|  | 90-92% | 4 | Severe anaemia | 1 |
|  |  |  | Developmental delay/cerebral palsy | 2 |
|  |  |  | Gastroenteritis/diarrhoea | 1 |
|  |  |  | LRTI - any (includes suspected TB) | 4 |
| A - low flow | <=80% | 7 | Heart condition, congenital or other | 2 |
|  |  |  | Developmental delay/cerebral palsy | 1 |
|  |  |  | HIV/AIDS | 1 |
|  |  |  | LRTI - any (includes suspected TB) | 7 |
|  |  |  | Malnourished | 2 |
|  | 81-89% | 7 | Heart condition, congenital or other | 2 |
|  |  |  | Developmental delay/cerebral palsy | 1 |
|  |  |  | HIV/AIDS | 2 |
|  |  |  | LRTI - any (includes suspected TB) | 7 |
|  |  |  | Malnourished | 2 |
|  |  |  | Sepsis/septicaemia | 1 |
|  | 90-92% | 4 | LRTI - any (includes suspected TB) | 4 |
|  |  |  | Other | 1 |
| B - high flow | 81-89% | 4 | Heart condition, congenital or other | 1 |
|  |  |  | LRTI - any (includes suspected TB) | 3 |
|  |  |  | URTI - any | 1 |
|  | 90-92% | 1 | Developmental delay/cerebral palsy | 1 |
|  |  |  | LRTI - any (includes suspected TB) | 1 |
|  |  |  | Malnourished | 1 |
| B - low flow | <=80% | 2 | Heart condition, congenital or other | 2 |
|  |  |  | Developmental delay/cerebral palsy | 1 |
|  |  |  | LRTI - any (includes suspected TB) | 2 |
|  |  |  | Severe malaria - all forms | 1 |
|  | 81-89% | 5 | Developmental delay/cerebral palsy | 1 |
|  |  |  | LRTI - any (includes suspected TB) | 5 |
|  |  |  | Severe malaria - all forms | 1 |
|  | 90-92% | 1 | LRTI - any (includes suspected TB) | 1 |
|  |  |  | Measles | 1 |
|  |  |  | Malnourished | 1 |
| B - permissive hypoxia | <=80% | 2 | Heart condition, congenital or other | 1 |
|  |  |  | LRTI - any (includes suspected TB) | 2 |
|  |  |  | Severe malaria - all forms | 1 |
|  | 81-89% | 18 | Severe anaemia | 1 |
|  |  |  | Gastroenteritis/diarrhoea | 2 |
|  |  |  | LRTI - any (includes suspected TB) | 18 |
|  |  |  | Sepsis/septicaemia | 3 |
|  |  |  | Severe malaria - all forms | 1 |
|  | 90-92% | 13 | Heart condition, congenital or other | 2 |
|  |  |  | LRTI - any (includes suspected TB) | 13 |
|  |  |  | Sickle cell disease | 1 |
|  |  |  | Severe malaria - all forms | 3 |

### **Table S4 Reasons for readmissions**

| **Stratum/Arm** | **Age (mths)** | **Reason for readmission** |
| --- | --- | --- |
| A - high flow | 1 | Readmitted and treated for Acute Gastro-enteritis for 5 days. |
| A - high flow | 8 | Readmitted for 6 days for Bronchiolitis |
| A - low flow | 11 | Child with Downs Syndrome, CHD (Tricuspid Regurgitation) admitted with Severe Anaemia and Pneumonia for 14 days. |
| A - low flow | 15 | Readmitted and treated for Severe Pneumonia, complicated Measles and managed for Moderate Malnutrition for 5 days |
| B - high flow | 3 | Congenital heart disease (Truncus arteriosus) was awaiting surgical correction. Readmitted and treated for severe pneumonia and heart failure |
| B - high flow | 117 | Readmitted and treated for severe *P. falciparum* malaria and septicaemia. |
| B - high flow | 14 | Known to have a CHD (Truncus Arteriosus corrected and on follow-up) readmitted and treated for severe pneumonia. |
| B - high flow | 29 | Originally admitted with severe malnutrition: readmitted and treated for severe  *P. falciparum* malaria and anaemia |
| B - high flow | 1 | Child with CHD (Atrial Septal defect), readmitted with severe pneumonia and sepsis. |
| B - high flow | 2 | Readmitted with severe  *P. falciparum* malaria and pneumonia. |
| B - high flow | 34 | Known to have sickle cell disease readmitted and managed for Sepsis (*E.faecalis* isolated),severe pneumonia, osteomyelitis and pathological bone fracture. |
| B - low flow | 9 | Severe  *P. falciparum* Malaria, Severe Pneumonia, Severe Malnutrition. |
| B - low flow | 8 | Readmitted and treated for 4 days for Bronchiolitis |
| B - low flow | 6 | Known CHD (ASD, VSD, PDA and an overriding aorta) readmitted and treated for severe pneumonia. |
| B - low flow | 10 | Readmitted with severe pneumonia. |
| B - low flow | 24 | Readmitted with severe anaemia and thrombocytopenia. |
| B - control | 4 | Readmitted with septicaemia and measles. |
| B - control | 6 | Readmitted and treated for severe pneumonia and acute gastroenteritis. |
| B - control | 17 | Readmitted and treated for bronchospasm, possible asthma and Pneumonia. |
| B - control | 29 | Readmitted and treated for Upper Respiratory Tract infection |
| B - control | 26 | Readmitted with severe pneumonia, severe malnutrition and measles. |
| B - control | 1 | Infant with Congenital Heart Disease (VSD, PDA) readmitted and treated for Severe Pneumonia |
| B - control | 23 | Readmitted and treated for 5 days for severe pneumonia. |
| B - control | 19 | Severe anaemia, lower respiratory tract infection, developmental delay and down syndrome |
| B - control | 37 | Readmitted with  *P. falciparum* malaria and septicaemia. |
| B - control | 4 | Lower respiratory tract infection and failure to thrive. |
| B - control | 9 | Re-admitted with Acute Gastroenteritis. |
| B - control | 5 | Readmitted for 3 days with lower respiratory tract infection, bronchiolitis and possible asthma |
| B - control | 10 | Readmitted and treated for 2 days for Acute Gastroenteritis with some dehydration. |
| B - control | 25 | Readmitted and treated for  *P. falciparum* malaria. |
| B - control | 50 | Readmitted and treated for severe  *P. falciparum* malaria. |
| B - control | 1 | Readmitted due to severe pneumonia and bronchospasms/asthma. |
| B - control | 14 | Readmitted for 3 days with severe pneumonia and bronchial asthma |
| B - control | 12 | Readmited with  *P. falciparum* malaria. |
| B - control | 4 | Readmitted due to Upper respiratory tract infection and an abscess around the neck. |
| B - control | 40 | Known Sickle Cell Disease readmitted and treated for painful crisis, pneumonia and sepsis. |
| B - control | 11 | Readmitted and treated Severe Pneumonia and Acute Gastroenteritis |

## FIGURES

### **Figure S1 Baseline SpO_2_ levels in children requiring oxygen and never requiring oxygen: control arm**

### **Figure S2** **Kaplan Meier Survival to Day 28 by Stratum**

|  |  |
| --- | --- |
|  | |

| Comparison | Unadjusted HR (95% CI) | Adjusted HR (95% CI) |
| --- | --- | --- |
| Any respiratory support vs permissive hypoxaemia (Stratum B only) | 0.93 (0.55, 1.57) | 0.91 (0.54, 1.54) |
| High flow (HFNT) vs. low flow | 0.79 (0.54, 1.15) | 0.79 (0.54, 1.16) |

### **Figure S3 Correction of Oxygen saturations over 48 hours**

**Stratum A**

**Stratum B**

### **Figure S4** **Total volume of oxygen used (litres) over 48 hours in HFNT and LFO groups**


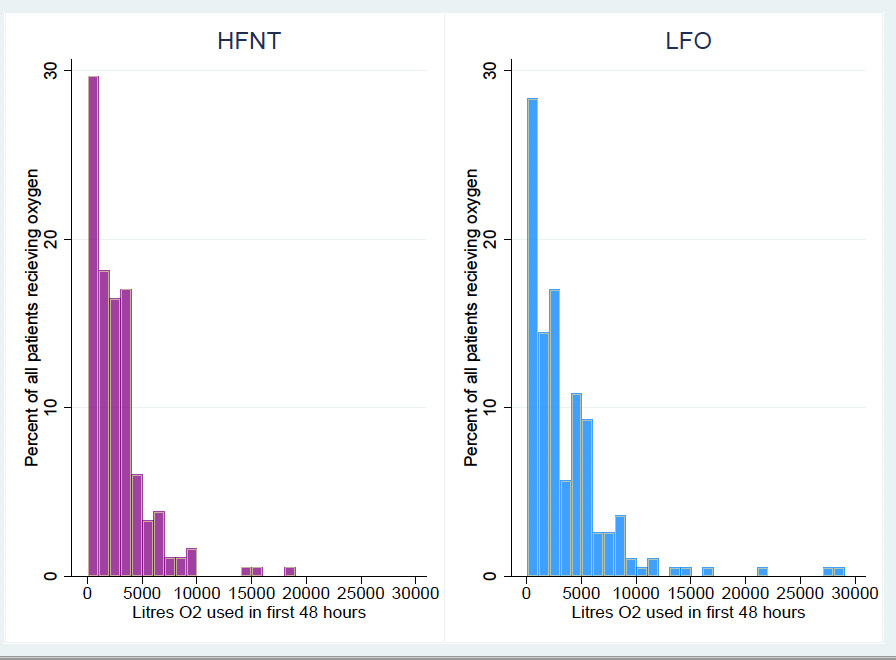


# REFERENCES

1. Dondorp AM, Fanello CI, Hendriksen IC, et al. Artesunate versus quinine in the treatment of severe falciparum malaria in African children (AQUAMAT): an open-label, randomised trial. Lancet 2010;376:1647-57.

2. Maitland K, Kiguli S, Opoka RO, et al. Mortality after fluid bolus in African children with severe infection. The New England journal of medicine 2011;364:2483-95.

3. Maitland K, Kiguli S, Olupot-Olupot P, et al. Immediate Transfusion in African Children with Uncomplicated Severe Anaemia. The New England journal of medicine 2019;381:407-19.

4. Maitland K, Olupot-Olupot P, Kiguli S, et al. Transfusion Volume for Children with Severe Anaemia in Africa. The New England journal of medicine 2019;381:420-31.

5. Hospital Care for Children. Guidelines for the management of common childhood illnesses. Second Edition. Geneva: World Health Organization; 2013.

6. Maitland K, Kiguli S, Opoka RO, et al. Mortality after fluid bolus in African children with severe infection. The New England journal of medicine 2011;364:2483-95.

7. Belle J, Cohen H, Shindo N, et al. Influenza preparedness in low-resource settings: a look at oxygen delivery in 12 African countries. Journal of infection in developing countries 2010;4:419-24.

8. Dauncey JW, Olupot-Olupot P, Maitland K. Healthcare-provider perceptions of barriers to oxygen therapy for paediatric patients in three government-funded eastern Ugandan hospitals; a qualitative study. BMC health services research 2019;19:335.

9. Maitland K, Kiguli S, Opoka RO, et al. Children's Oxygen Administration Strategies Trial (COAST): A randomised controlled trial of high flow versus oxygen versus control in African children with severe pneumonia. Wellcome Open Res 2017;2:100.

10. Landau S, Stahl D. Sample size and power calculations for medical studies by simulation when closed form expressions are not available. Stat Methods Med Res 2013;in press.

# APPENDIX

# STATISTICAL ANALYSIS PLAN- PDF
