# Supplementary material for: Randomised controlled trial of oxygen therapy and high-flow nasal therapy in African children with pneumonia
Source: Intensive Care Med. 2021 May 5;47(5):566–76. doi: 10.1007/s00134-021-06385-3 (PMC8098782; doi:10.1007/s00134-021-06385-3)
Supplement: Supplementary file 2 — Supplementary file1 (PDF 1539 KB) [file 134_2021_6385_MOESM2_ESM.pdf]

Children's Oxygen Administration Strategies Trial: COAST

**Protocol version:**	2.1
**Protocol version date:**	11th January 2017

**ICREC number:**	**15IC3100**
**Trial Sponsor:**	Imperial College London
**Sponsor reference:**	**P46493**
**ISRCTN number:**	**ISRCTN15622505**
**Trial Funder:**	Joint Global Health Trials scheme:<br>Medical Research Council<br>Department for International Development<br>Wellcome Trust
**Funder reference:**	MR/L004364/1

**Role**	**Name**	**Signature**	**Date**
Chief Investigator:	Prof Kathryn Maitland	Kathryn Maitland	11th Jan 2017
TSC Chair:	Prof Elizabeth Molyneux		

Trial Management

Trial Coordination Centre:	Phyles Maitha
	Kilifi Clinical Trials Facility (KCTF)<br>Kenya Medical Research Institute (KEMRI) Wellcome Trust Research Programme<br>P.O Box 230-80108<br>Kilifi<br>Kenya
Telephone:	+254 715461761
Email:	PMaitha@kemri-wellcome.org

Clinical Trials Unit:	Intensive Care National Audit & Research Centre (ICNARC)<br>Napier House<br>24 High Holborn<br>London WC1V 6AZ<br>United Kingdom
Telephone:	+44 (0)20 7269 9277
Email:	ctu@icnarc.org

Sponsor:	Imperial College London<br>AHSC Joint Research Compliance Office<br>5th Floor Lab Block<br>Charing Cross Hospital<br>Fulham Palace Road<br>London W6 8RF<br>United Kingdom
Telephone:	+44 (0) 20 3311 0208
Email:	a.alcock@imperial.ac.uk (JRCO coordinator)

Funder Representative:	Kori Cook
	Science Portfolio Adviser<br>Wellcome Trust<br>215 Euston Road<br>London NW1 2BE<br>United Kingdom
Telephone:	+44 (0) 20 7611 8888
Email:	k.cook@wellcome.ac.uk

Partner:	Dr Stanislav Tatkov
	Clinical Research Manager<br>Fisher & Paykel HealthCare (donating Airvo & consumables)
Telephone:	+64 9 574 0123 ext 7938
Email:	Stanislav.Tatkov@fphcare.co.nz

Clinical Management

For any urgent clinical support please contact Mr Ayub Mpoya in the first instance (contact details below) who will consult other members of the Trial Management Group (TMG) where necessary.

Clinical support:	Mr Ayub Mpoya
	KEMRI Wellcome Trust Research Programme<br>Clinical Trials Facitlity, Kilifi, Kenya
Telephone:	+254 735229037
Email:	AMpoya@kemri-wellcome.org

Trial Investigators

Chief Investigator

Prof Kathryn Maitland	Professor of Tropical Paediatric Infectious Diseases
	Wellcome Trust Centre for Global Health Research,<br>Imperial College London,<br>Faculty of Medicine, Room 232, Wright Fleming Institute,<br>St Marys Campus, Norfolk Place,<br>London W2 1PG UK

Co-investigators

Prof Sarah Kiguli	Professor/Head of Paediatrics Department (Country Principal Investigator (PI)
	Makerere University, Uganda

Dr Robert Opoka	Consultant Paediatrician/Epidemiologist (Site PI)
	Makerere University, Uganda

Dr Peter Olupot-Olupot	Medical Doctor/Clinical Scientist (Site PI)
	Mbale Regional Referral Hospital, Uganda

Dr Charles Engoru	Consultant Paediatrician (Site PI)
	Soroti Regional Referral Hospital, Uganda

Dr David Harrison	Senior Statistician
	ICNARC, UK

Prof Kathy Rowan	Director of Scientific & Strategic Development/CTU Director
	ICNARC, UK

Prof Richard Grieve	Professor of Health Economics Methodology
	London School of Hygiene & Tropical Medicine (LSHTM), UK

Dr Zia Sadique	Lecturer in Health Economics
	LSHTM, UK

Prof Thomas Williams	Professor of Haemoglobinopathy Research
	Wellcome Trust Centre for Global Health Research,<br>Imperial College London, UK

Prof Andrew Bush	Professor of Paediatric Respirology
	National Heart and Lung Institute, Royal Brompton & Harefield NHS Foundation Trust, Imperial College London, UK

Collaborators

Professor John Fraser	Professor of Critical Care
	Critical Care Research Group, Adult Intensive Care Services The Prince Charles Hospital, Queensland, Australia
Professor James Nokes	Professor of Mathematical Biology
	KEMRI Wellcome Trust Research Programme, Kilifi, Kenya

Site Investigators

Prof Sarah Kiguli	Associate Professor/Head of Paediatrics Department (Chief PI, Uganda)
	Makerere University, Uganda

Dr Robert Opoka	Consultant Paediatrician/Epidemiologist (Site PI)
	Makerere University, Uganda

Dr Peter Olupot-Olupot	Medical Doctor/Clinical Scientist (Site PI)
	Mbale Regional Referral Hospital, Uganda

Dr Charles Engoru	Consultant Paediatrician (Site PI)
	Soroti Regional Referral Hospital, Uganda

Dr Patricia Njuguna	Head of Clinical Research (Site-PI)
	KEMRI Wellcome Trust Programme, Kilifi, Kenya Kilifi County Hospital, Kilifi, Kenya

Dr Victor Bandika	Consultant Paediatrian/Head of Department (Site-PI)
	Coast Provincial General Hospital, Kenya

This protocol describes the COAST trial and provides information about procedures for entering participants. The protocol should not be used as a guide for the treatment of other participants; every care was taken in its drafting, but corrections or amendments may be necessary. Any amendments will be circulated to investigators in the study, but centres entering participants for the first time are advised to contact the trials centre to confirm they have the most recent version. Problems relating to this trial should be referred, in the first instance, to the study coordination centre.

This trial will adhere to the principles outlined in the International Conference on Harmonization Good Clinical Practice (ICH-GCP) guidelines. It will be conducted in compliance with the protocol, the Data Protection Act and other regulatory requirements as appropriate.

Table of Contents

Trial Management 2
Trial Investigators 3
Table of Contents 5
Abbreviations 9
Glossary of terms 10
Keywords 10
Trial summary 11
Trial flow 12
1 Introduction 13
Clinical burden of pneumonia 13
Hypoxia in ‘pneumonia’ syndromes 13
Current treatment recommendations for pneumonia 14
Availability of therapeutic oxygen: technical and clinical challenges 15
Evidence from clinical trials and systematic reviews 16
Who needs oxygen? 16
Evidence for a threshold for delivery of oxygen 17
Evidence from other sources 18
Risk of mortality modelled against oxygen saturation 18
Oxygen administration: evidence for delivery methods 20
Trials and systematic reviews of oxygen delivery systems 21
1.1 Rationale for current study 21
Justification for a controlled trial 21
Research gap: timeliness for a clinical trial 22
Benefits 22
Risks 23
2 Trial aim and objectives 25
2.1 Primary objectives 25
2.2 Secondary objectives 25
3 Trial outcome measures 26
3.1 Primary 26
3.2 Secondary 26
3.3 Trial design 26
4 Selection of sites 27
4.1 Site/Investigator inclusion criteria 27

4.2 Documentation 27
4.3 Activation 28
5 Selection of participants 28
5.1 Screening 28
5.2 Inclusion criteria 29
5.3 Exclusion criteria 29
6 Enrolment 30
6.1 Consent 30
6.2 Emergency verbal assent followed by deferred consent 30
6.3 Randomisation 30
6.4 Co-enrolment 31
6.5 Withdrawal 31
7 Trial treatment 32
7.1 Randomisation allocation and treatment arms 32
7.2 Duration of treatment 35
7.2.1 Standard clinical management 35
7.3 Trial equipment 35
8 Data collection 36
8.1 Data collection – participants 38
8.1.1 Baseline – characteristics 38
8.1.2 Baseline – laboratory tests 38
8.1.3 Hospital stay 38
8.1.4 Hospital stay – additional laboratory tests 39
8.1.5 Hospital discharge 39
8.1.6 Follow-up 39
8.1.7 Health economics 39
8.1.8 Outcomes 40
8.2 Data collection – site staff 40
8.2.1 Nurses 40
8.2.2 Doctors 40
8.2.3 Trial site coordinator 41
9 Data management 41
9.1 Sample storage 41
10 Safety monitoring 41
10.1 Definitions 41
10.2 Severity 42

10.3 Relatedness 42
10.4 Expectedness 43
10.5 Exempt adverse events 43
10.6 Recording and reporting 43
10.7 Central processing 43
10.8 Additional monitoring and notification process 44
11 Trial monitoring and oversight 45
11.1 Site 45
11.2 Site Trial Management Team 45
11.3 Trial Management Group 46
11.4 Trial Steering Committee 46
11.5 Data Monitoring Committee 46
11.6 Endpoint Review Committee 46
12 Trial Closure 47
12.1 End of trial 47
12.2 Archiving 47
12.3 Early discontinuation 47
13 Statistics 48
13.1 Sample size calculation 48
13.2 Analysis plan 48
13.3 Interim analysis 49
13.4 Ancillary Studies 49
a. Economic and cost-effectiveness evaluation 49
b. Molecular diagnostics 49
c. Investigation of respiratory viruses 50
d. Electrical Impedance Tomography (EIT) 50
e. Short PeRiod INcidence sTudy of Severe Acute Respiratory Infection (SPRINT-SARI)51
f. Sickle cell disease status 51
14 Ethical compliance 52
14.1 Trial registration 52
14.2 Central ethical compliance 52
14.3 Local ethical compliance 52
14.4 Confidentiality and data protection 52
14.5 Patient, Carer and Public Involvement and Engagement 52
14.6 Declaration of interests 53
15 Sponsorship and Funding 53

15.1 Sponsorship 53
15.2 Funding 53
16 Dissemination policy 53
References 56
Appendix 61
Appendix A Protocol version history 61
Appendix B Expected adverse events 62
Appendix C Parent/Guardian Information Sheet 63
Appendix D Consent Form 68
Appendix E Verbal Assent Form 69
Appendix F Withdrawal Form 70

Abbreviations

16S rDNA	16S ribosomal deoxyribonucleic acid
AE	adverse event
CAB	Community Advisory Board
CI	confidence interval
ChI	Chief Investigator
CO_2	carbon dioxide
COAST	Children's Oxygen Administration Strategies Trial
CPAP	continuous positive airway pressure
CPGH	Coast Provincial General Hospital
CRF	Case Report Form
CTU	Clinical Trials Unit
DFID	Department for International Development
DMC	Data Monitoring Committee
DNA	deoxyribonucleic acid
ERC	Endpoint Review Committee
FiO_2	oxygen concentration
HbSS	Sickle Cell Disease
ICH-GCP	International Conference on Harmonization Good Clinical Practice
ICNARC	Intensive Care National Audit & Research Centre
IQR	interquartile range
KCTF	Kilifi Clinical Trial Facility
KCH	Kilifi County Hospital
KEMRI	Kenya Medical Research Institute
LRTI	lower respiratory tract infection
MRC	Medical Research Council
NP	nasal prongs
NPC	nasopharyngeal catheters
PCR	polymerase chain reaction
PI	Principal Investigator
POST	Paediatric Oxygenation Strategies Trial
qPCR	specific quantitative polymerase chain reaction
RCT	randomised controlled trial
RDT	Rapid diagnostic test
REC	Research Ethics Committee
RNA	ribonucleic acid
RR	relative risk
RSV	Respiratory Syncytial Virus
RV	respiratory virus
SAE	serious adverse event
SpO_2	oxygen saturation
SCD	sickle cell disease
SIV	site initiation visit
SP	severe pneumonia
TMG	Trial Management Group
TSC	Trial Steering Committee
WGS	whole genome sequencing
WHO	World Health Organization
VSP	very severe pneumonia

Glossary of terms

High flow oxygen	Flow rates 1 L/kg/min – 2 L/kg/min (up to 60 L/min max)
Low flow oxygen	Standard flow 1-2 L/min [1]
Respiratory distress	Deep breathing or increased work of breathing (indrawing)
Permissive hypoxia	Maintaining oxygen saturation (SpO_2) levels between **80-91**%

Keywords

Children
Infants
Africa
Emergency Care
Clinical trial
Uganda
Kenya
Democratic Republic of Congo
Hypoxia
Pneumonia
Respiratory distress
Respiratory failure
Randomised controlled trial
High flow oxygen
Low flow oxygen

Trial summary

Title (ACRONYM)	Children's Oxygen Administration Strategies Trial (COAST)
Version and Date	2.1, 25th November 2016
ISRCTN	**15622505**
Trial design	Open, multicentre, fractional factorial randomised controlled trial
Participants	4,200 children aged between 28 days and 12 years with respiratory distress complicated by hypoxia (defined as SpO_2 <92%)
Trial hypotheses	• To establish whether liberal oxygenation for SpO_2 ≥80% will decrease mortality (at 48 hours and up to 28 days) compared with a strategy that includes permissive hypoxia (usual care); and<br>• To establish whether use of high flow oxygen delivery will decrease mortality (at 48 hours and up to 28 days) compared with low flow oxygen delivery (usual care)
Primary outcome	Mortality at 48 hours post-randomisation
Secondary outcomes	• Treatment failure at 48 hours<br>• Survival to 28 days<br>• Neurocognitive sequelae at 28 days<br>• Disability-free survival to 28 days<br>• Time to hypoxia (≥92%) resolution during initial hospital stay<br>• Length of initial hospital stay<br>• Re-admission to hospital by 28 days<br>• Anthropometric status by 28 days<br>• Resolution of neurocognitive sequelae at 90 days (for those with neurocognitive sequelae at 28 days)
Randomisation and Interventions	The trial has two strata: **COAST A**: severe hypoxia, SpO_2 <80%); and **COAST B**: hypoxia SpO_2 ≥80% and <92%).<br><br>Children in COAST A (2-arm, 1:1 ratio) will all receive oxygen and randomisation will allocate participants to one of two methods of oxygen delivery:<br>i. high flow oxygen delivery<br>ii. low flow (usual practice) oxygen delivery<br><br>Children in COAST B (3-arm, 2:1:1 ratio) will be allocated to:<br>i. permissive hypoxia (no immediate oxygen): control<br>ii. high flow oxygen delivery<br>iii. low flow oxygen delivery
Duration of recruitment	36 months
Definition of end of trial	Last participant, last follow-up
Ancillary studies	• Economics and cost-effectiveness evaluation<br>• Molecular diagnostics<br>• Investigation of respiratory viruses<br>• Electrical Impedance Tomography<br>• SPRINT-SARI study<br>• Sickle cell disease status
Sponsor	Imperial College London
Funder	Joint Global Health Trials scheme (Medical Research Council, Department for International Development and Wellcome Trust)
Chief Investigator	Prof Kathryn Maitland

Trial flow

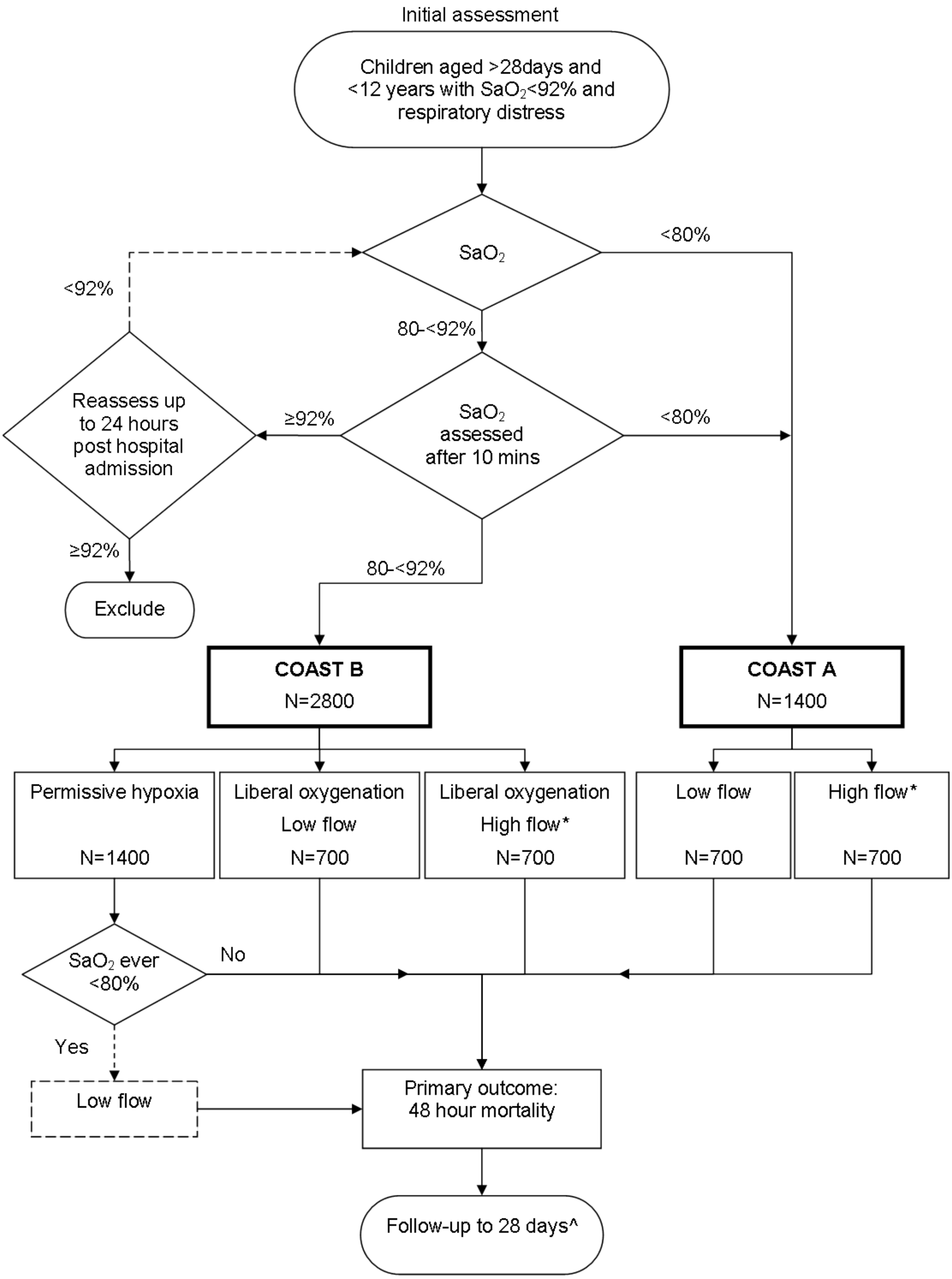

*Maximum of 48 hours

^Follow-up to 90 days for those with evidence of neurological sequelae at 28 days

1 Introduction

Worldwide, the clinical syndrome of pneumonia remains the leading cause of death in children in the post-neonatal period and presents a significant burden on in-patient services [2, 3]. Over 70% of these deaths occur in South East Asia and sub-Saharan Africa [4]. The World Health Organization (WHO) recommends presumptive antibiotic treatment based on clinical syndromic definitions of pneumonia plus oxygen for those with clinically very severe pneumonia (VSP) or hypoxia – defined as oxygen saturation (SpO_2) <90% [5]. Even with recommended treatment, there is substantial evidence that current syndromic management guidelines are not working in practice, resulting in high mortality (9-16%) [6, 7]. Hypoxia is present in 9.4% and 13.3% of children hospitalised with severe pneumonia (SP) and VSP respectively and is both an important indicator of disease severity and a key predictor of mortality [8]. The targeted use of oxygen and simple, non-invasive methods of respiratory support may be a highly cost-effective means of improving outcome, but both the optimal oxygen saturation threshold which results in benefit and the best strategy for delivery are yet to be tested in adequately powered randomised controlled trials (RCT). Oxygen as a potentially life-saving treatment, though advocated by the WHO technologies group, has not been afforded a high enough priority at either country or global levels [9]. Systematic and policy reviews indicate the need for a formal evaluation of the hypoxia threshold at which oxygen should be targeted and of how oxygen is best administered [8, 10, 11]. The COAST trial aims to address these key research gaps to provide a better evidence base for future guidelines, with a view to improving poor outcomes.

Clinical burden of pneumonia

Pneumonia is the leading cause of childhood death in sub-Saharan Africa [2]. Substantial investment in international multi-country research and vaccine implementation will undoubtedly reduce the burden of disease and lead to refinements of current guidelines. The current WHO clinical criteria for identification of pneumonia aim at prioritising sensitivity over specificity. Thus, emerging results from prospective studies of hospitalised children with WHO criteria for pneumonia indicate that it encompasses a much broader range of aetiologies, particularly in sub-Saharan Africa, than those likely to be averted by current and planned public health measures such as immunisation programmes or early identification and pre-hospital antimicrobial treatment [6, 12]. Adverse outcomes are not uncommon, despite early diagnosis and treatment [6, 12, 13]. As the clinical syndrome of pneumonia is so very common amongst hospitalised children, the high early mortality remains a significant barrier to improving survival statistics [12-14] and important for attainment of Millennium Developmental Goals' targets.

For example, the admission cohort of Kenyan children, reported below, demonstrated that 60% of children that fulfilled VSP criteria had a non-pneumonic cause of respiratory distress [6]. At the same centre, this was further explored in a case-control study examining pathogenic aetiology of children aged <5 years hospitalised with SP, prior to the introduction of pneumococcal vaccine. Bacterial aetiology was established in 23%, viral in 13%, mixed in 2% and 63% were of unknown aetiology [12]. No association between viral infection of the nasopharynx and pneumonia was found, except for respiratory syncytial virus. All deaths had no identifiable pathogen. In Fiji, amongst children with a discharge diagnosis indicating a LRTI, only 34% met WHO standardised criteria for chest x-ray confirmed pneumonia [15].

Hypoxia in 'pneumonia' syndromes

A systematic review examining the prevalence of hypoxia, including 24 published and unpublished data sets from Africa, Asia/Oceania and Latin America, reported a median prevalence of hypoxia for SP was 9.4% (interquartile range (IQR) 7.5–18.5%) and 13.3% (IQR 9.3–37.5%) for those with VSP [8]. Mortality rates were not reported. Conservative estimates, based on these data, indicated that the annual worldwide burden of hypoxia in hospitalised pneumonia alone to between 1.5–2.7 million cases.

The importance of hypoxia, as a key predictor of mortality, is underlined in prospective studies examining mortality in unselected hospital cohorts of pneumonia. In an unselected admission population of 13,183 Kenyan children, hypoxia was present in 693 (5.3%) of the children admitted (median age 18.9 months) (Figure 1) [6]. The most frequent final diagnoses among hypoxaemic children were malaria (244, 35%), lower respiratory tract infection (LRTI) (221, 32%), malnutrition (68, 10%) and gastroenteritis (49, 7%). Severe anaemia was found in 30 children (<1%). Overall, 753 (6%) children died, including 150 (22%) of those with hypoxia.

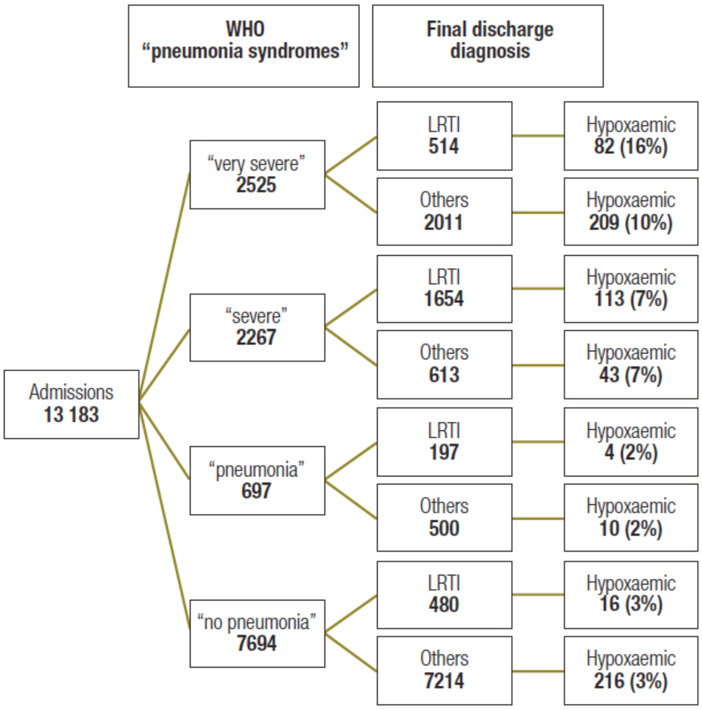

Figure 1 Lower respiratory tract infections and other non-respiratory diagnoses in 13,183 Kenyan children admitted to hospital

In Indonesia, children aged <2 years admitted with SP (overall 12% hospital mortality) demonstrated that hypoxic thresholds associated with increased mortality differed by age [16]. For children aged <4 months this was SpO_2 <88% whereas for children aged >4 months it was <80%.

Current treatment recommendations for pneumonia

WHO Guidelines for management of children in hospital in resource-limited settings recommend presumptive antibiotic treatment based on clinical syndromes involving history of cough or difficulty breathing with lower chest wall in drawing, or signs indicating VSP; it also recommends oxygen for those with VSP or hypoxia (Table 1) [5]. The normal range of acceptable SpO_2 values differs according to age and altitude. At sea level, normal values are generally

considered to be a SpO_2 >94%. Thresholds for administering oxygen differ among international guidelines – some target <92%, whilst others target <94%. The lack of evidence that maintaining 'normal' oxygen values in critically ill patients is beneficial possibly explains the wide variation in practice surrounding the management of hypoxia. For children in resource-poor countries, the WHO recommended threshold for giving oxygen therapy is SpO_2 <90%, or SpO_2 <87% if living at altitude >2500m [10]. In the 2013 WHO guidelines, the revision of the definitions of the categories of pneumonia mean that VSP and SP were collapsed into one category ('Severe pneumonia') and the definition included in a child with a history of cough and difficulty one or more of signs of putative pneumonia, including with respiratory distress, evidence of hypoxia (central cyanosis or oxygen saturations of <90%) or danger signs (lethargy or greater, convulsions). Oxygen is recommended for those with oxygen saturations of <90%, yet most hospitals in developing countries lack the facility to measure this.

Table 1 WHO guidelines recommended treatment for pneumonia

Type	Where to treat	Oxygen
**WHO 2005 recommendations [4]**		
**VSP**	Inpatient	Give to all children with VSP<br>If pulse oximetry available, give to children with oxygen saturation <90%<br>Use nasal prongs (NP), nasal catheter, or nasopharyngeal catheter (NPC)
**SP**	Inpatient	If readily available, give to any child with severe lower chest wall in drawing or a respiratory rate of >70/minute
**Pneumonia**	Outpatient	Not required
**WHO 2013 recommendations [17]**		
**Severe pneumonia**	Inpatient	Give if oxygen saturation <90%<br>Use NPs as the preferred method of oxygen delivery to young infants
**Pneumonia**	Outpatient	Not required

Availability of therapeutic oxygen: technical and clinical challenges

There is substantial evidence that even after substantial investment for influenza epidemic preparedness, there are significant gaps between supply and demand for oxygen in most hospitals in developing countries [18]. Technical reports on the operational quality, availability and reliability of cylinders or oxygen concentrators indicate that, even when available, these are often faulty (substantial leakage from cylinders and lack of maintenance for both) and/or unsustainable due to high cost, erratic supply chain or dependence upon unreliable electricity supply [18-21]. However in practice, this results in severe shortages of oxygen and intermittent supply of oxygen (where power permits).

Few hospitals have access to pulse oximeters [22] and the sustainable provision of bottled oxygen or oxygen concentrators are both expensive and logistically challenging [23]. As a consequence, many children who fulfil the current criteria for oxygen do not receive it (standard 'usual care'). Instead, the current reliance on non-specific clinical signs to guide oxygen therapy

results in the poorly targeted use of a costly intervention [6] – as a result few children who may benefit from oxygen therapy actually receive it.

The 2012 WHO Recommendations for management of common childhood conditions identified a number of key research questions as 'Research Gaps', which included: large-scale effectiveness trials of improved oxygen systems on outcomes from pneumonia; and clinical studies comparing outcomes when oxygen is given at different thresholds [10].

Evidence from clinical trials and systematic reviews

Both inadequate tissue oxygenation and excessive oxygen administration are likely to be detrimental to outcome. There are two components to taken into consideration when reviewing evidence for oxygen treatment guidelines: what is the evidence supporting beneficial use of oxygen and at what threshold (oxygen saturation); and what is the best method for its delivery. For the latter consideration, for resource-poor setting without access to intensive care (and mechanical ventilation), this will mainly consider non-invasive methods.

Who needs oxygen?
A Cochrane review conducted in 2005 [11] and updated in 2014 [24] examined evidence in the literature for: (i) indications for oxygen therapy including both observational studies and RCTs comparing oxygen versus no with oxygen therapy; and (ii) RCTs comparing methods of oxygen delivery for hypoxic LRTI in children aged three months to 15 years [24]. Of the 551 articles assessed, there was no study that compared delivering oxygen with not delivering oxygen. Of the eligible studies, four RCTs compared delivery methods (see *Trials and Systematic Reviews of oxygen delivery systems*) and 14 observational studies assessed the accuracy of clinical signs for indicating hypoxia. The review found no single clinical sign or symptom accurately identified hypoxia, although pneumonia complicated by hypoxia increased the risk of death [25]. Wider implementation of pulse oximeters was recommended to identify those at greatest risk and for studies examining the most effective and safe method for oxygen delivery.

Thus, irrespective of the availability of mechanical ventilation, there have been no controlled trials of the use of oxygen supplementation that have incorporated a randomisation strategy of no oxygen. In resource-poor setting, there are limited relevant data from epidemiological studies for children receiving oxygen, or not, However, a review of available literature indicated little difference in mortality in children with pneumonia and SpO_2 >80% receiving or not receiving supplementary oxygen [26]. The only prospective observational study conducted to date examined mortality before and after improving systems for oxygen delivery in five hospitals in Papua New Guinea (that had no access to mechanical ventilation). The study found a lowering mortality in children with pneumonia from 5.0% (95% confidence interval (CI) 4.5–5.5) to 3.2% (95% CI 2.7–3.8) for the 27 months after the improved system was introduced. Estimated costs of this system were US$51 per patient treated, US$1673 per life saved, and US$50 per disability-adjusted life-year averted [14]. Nevertheless, there are a number of alternative explanations for improved outcomes, including possible benefits from training, improvements in the delivery of emergency care and/or other components of treatment, patient monitoring or temporal changes, which limit generalisability since the study was not designed to address direct benefits to the individual.

Conditions in which oxygen therapy no longer recommended

Oxygen has been used in the treatment of pneumonia for a large part of the last century. However, the recognition of pulmonary oxygen toxicity as a problem has been relatively recent. Toxicity is related to the concentration of oxygen and length of exposure. Oxygen causes tracheobronchial irritation, reduced mucocillary function (even in healthy volunteers exposed to 90-95% oxygen for three hours [27]) and, eventually, adsorption atelectasis, decreased vital capacity and changes similar to adult-type respiratory distress syndrome[28]. The biochemical basis is believed to be damage to tissues through the production of reactive oxygen species. For these reasons, other than emergency usage of 100% oxygen, it is recommended that inspired oxygen concentration (FiO_2) should be carefully titrated against SpO_2.

In several areas of emergency care and resuscitation, the use of oxygen is now being challenged by emerging evidence from clinical trials and systematic reviews. In neonates, use of 100% oxygen during resuscitation increases mortality, myocardial injury and renal injury [9]; even following an asphyxiating perinatal event it is thought to increase the risk of cerebral damage [29]. Current resuscitation guidelines in neonates now advise that the initial gas administered for ventilation should be room air[30].

In patients with an acute myocardial infarct, systematic reviews have found that, compared with room air, there is no evidence that oxygen therapy is of benefit [31]; this is similarly the case in patients with an acute stroke [32]. A recent trial in adults with asthma has shown that high concentration oxygen therapy results in a clinically significant increase in carbon dioxide (CO_2), recommending careful titration of oxygen and only to those who are hypoxaemic [33]. Much of the concern for optimising oxygenation has centred on neuroprotection or prevention of cognitive deficits in the long term but the evidence supporting this is weak. In a follow-up study of mechanically ventilated adult patients with acute lung injury, a lower partial pressure of arterial oxygen (but not oxygen saturation) was associated with cognitive impairment [34]. No equivalent studies are available for paediatric cohorts. A systematic review in children with chronic or recurrent hypoxia indicated adverse effects on development, behaviour, and academic achievement; however generalising these findings are complicated since most studies had small sample sizes and did not stratify by SpO_2 [35].

Evidence for a threshold for delivery of oxygen

We were unable to find any data which prospectively determined a threshold for delivery of oxygen; in any setting, only observational studies reported mortality for those with and without hypoxia. One review from 1993 examined mortality in historic retrospective studies (1920's) of children with acute respiratory tract infection with or without oxygen therapy [26]. Whilst mortality rates were higher in those receiving and not receiving oxygen, these data are not informative since the *a priori* risk in the two groups are likely to be very different. In 2015, a parallel-group, randomised, controlled, equivalence trial of oxygen threshold strategies in infants aged 6 weeks to 12 months with clinician diagnosed bronchiolitis was reported. The two strategies were compared targeting an oxygen saturation threshold of 90% (n=308) and 94% (n=307). The primary outcome was time to resolution of cough (since this was considered to reflect the degree of airway inflammation and thus likely to influence degree of hypoxia). In both arms median time to cough resolution was 15 days (95% CI for difference –1 to 2), thus oxygen thresholds were equivalent. Adverse events were similar in both arms [36]. The study concluded that management of infants with bronchiolitis to an oxygen saturation target of 90% or higher is as safe and clinically effective; this suggested further studies in older children examining oxygen thresholds are needed, particularly in developing nations where resources are scarce. Currently,

there are no other planned trials examining optimum thresholds for oxygen delivery.

The WHO review of evidence, recommendation and key questions for future research is more comprehensive for the management of hypoxemia and oxygen delivery than any other of the WHO recommendations covering eight pages of the 'Technical update of pocket book: evidence review of WHO recommendations' [10]. Pertinent to the questions being addressed by the COAST trial are those examining the use of oxygen therapy and delivery systems. These are given under the actual number section headings contained within the review (pages 69-76):

Section 10.3 Oxygen therapy in treatment of hypoxia (Page 72)

a) Children with hypoxia should receive appropriate oxygen therapy.
(Strong recommendation, low quality evidence)

b) Effective oxygen delivery systems should be a universal standard of care, and should be made more widely available.
(Strong recommendation, expert opinion)

Section 10.4 Thresholds for administering oxygen therapy (Page 73)

a) Administering oxygen therapy should be guided by pulse oximetry where available and thresholds for giving oxygen may vary depending on the altitude.
(Strong recommendation, very low quality evidence)

b) Children living at ≤2500 m above sea level should receive oxygen therapy if their oxygen saturation is <90%.
(Strong recommendation, very low quality evidence)

c) In children living at high altitude (>2500m above sea level), the normal oxygen saturation is lower than those living at sea level. At these altitudes, a lower level of saturation, such as SpO_2 ≤87%, could be used as a threshold for giving oxygen. *(Strong Recommendation, very low quality evidence)*

In summary, most of the evidence for current recommendations is of very low quality; the use of oxygen is rated as expert opinion rather than based on evidence.

Evidence from other sources

Risk of mortality modelled against oxygen saturation
We examined the relationship between oxygen saturation at admission (or baseline) and mortality by 48 hours (or discharge date), using a fractional polynomial logistic model restricted to the baseline measure, in the Kilifi (KEMRI Wellcome Trust Programme) dataset of paediatric general admissions (n=36,036; unselected admissions) (Figure 2). The same relationship was also modelled and examined in more critically ill children using the control arm group of the FEAST Trial (n=1007) (Figure 3). The trial eligibility criteria included children with severe illness (and shock), thus is equivalent to the inclusive definition of VSP (respiratory distress (83%) and/or impaired consciousness (72%)) [37]. Both datasets indicated a steady increase of risk of mortality as SpO_2 decreases from 100% to 80%, with an inflection of the risk of mortality at around 80%. The wider 95% CIs at low values in the FEAST dataset are due to low numbers but is less pronounced for SpO_2 ≥80% (unpublished data).

We also created a model to include an interaction between oxygen saturation and severe anaemia (defined as haemoglobin <5 g/dl), again using the FEAST control arm group (Figure 4). This surprisingly did not appear to influence mortality risk, even though putatively reduces oxygen delivery to tissues. Similar analyses on the FEAST cohort in children with and without malaria/sepsis found no effect on risk of mortality (not shown). These data provide support for pragmatic inclusion criteria since anaemia, malaria and sepsis are common among paediatric admissions in Africa.

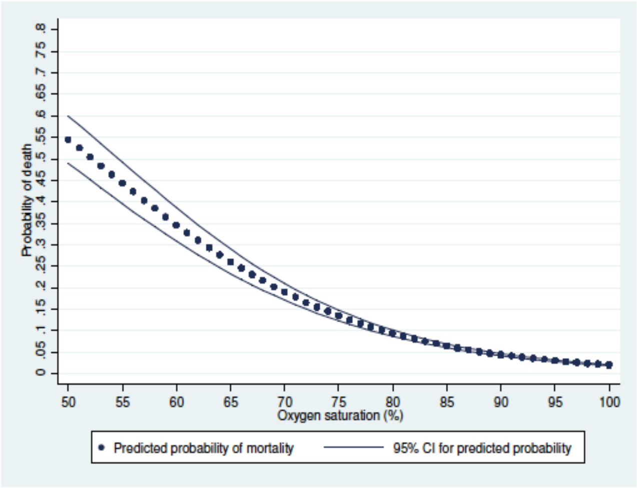

Figure 2 Relationship between baseline oxygen saturation and 48-hour mortality in 36,036 Kilifi paediatric general admissions

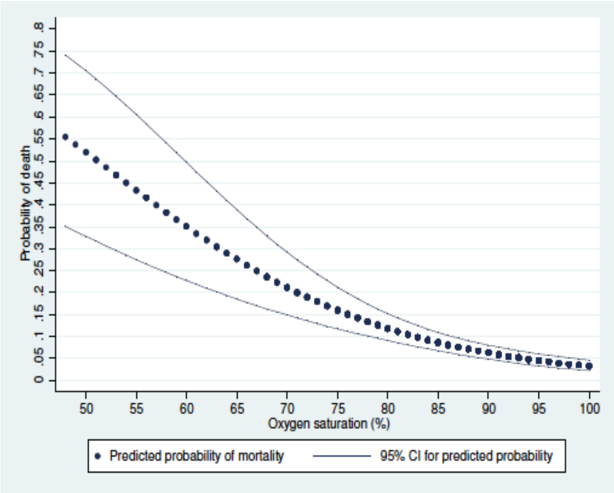

Figure 3 Relationship between baseline oxygen saturation and 48-hour mortality in the FEAST control arm (n=1007)

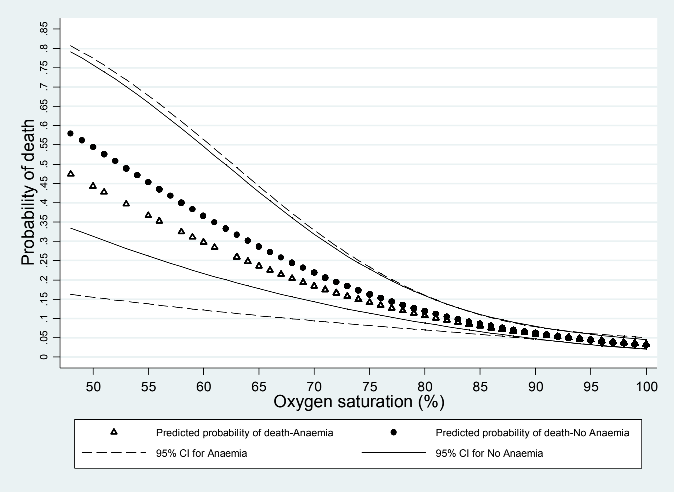

Figure 4 Relationship between baseline oxygen saturation and 48-hour mortality in those with and without severe anaemia in the FEAST control arm (n=1007)

Oxygen administration: evidence for delivery methods

There are many ways of giving oxygen [38, 39], however the WHO recommend NPs or NPCs for ease of delivery, patient acceptability and safety [10]. Facemask delivery of oxygen requires much higher oxygen flow rates (to wash out the CO_2), and feeding can interfere with its supply, thus is more costly. NPC requires the lowest oxygen flow rates, thus providing more reliable FiO_2, but need careful insertion as, if misplaced, could lead to gastric dilation or accidental dislodgement into the trachea. With NPs, a flow rate of 1-2 L/min in a child aged >4 months should provide a FiO_2 of 30-35%; however, this varies with inspiratory flow, weight and degree to which the child breathes through their mouth [40]. These are important logistical issues for optimising oxygen delivery.

High flow oxygen therapy

High flow nasal cannula (HFNC) oxygen delivery is a relatively new therapy that delivers a mixture of air and oxygen via tubing that sits just inside the nostrils. The flow rate delivered varies depending on the type of cannula used but can range from 4 to 70 L/min. HFNC results in incidental delivery of positive end expiratory pressure (PEEP) [41-43] and the amount of PEEP generated depends on the flow delivered/body mass and the potential for leak around the nasal cannula [44, 45]. Whilst not fully understood it have been suggested that the high flow flushes the dead space of the nasopharyngeal cavity, resulting in alveolar ventilation as a greater fraction of minute ventilation and also assists with washout of carbon dioxide[41]. High flow rates may also provide some amount of positive pressure and thereby overcome upper airway obstruction, again improving ventilation [46]. HFNC therapy has also been reported to be better tolerated by the patient than other forms of non-invasive ventilation, partly due to the use of HFNC oxygen therapy with heated and humidified gases [47].

The Brisbane Critical Care group has worked with high-flow oxygen devices and shown they reduce the requirement for positive pressure ventilation in the paediatric population with respiratory failure secondary to infection [48, 49]. The AIRVO device is adaptable for children over a wide weight and age range. A number of physiological effects have been described with high flow nasal oxygen delivery such as pharyngeal dead space washout, a positive expiratory pressure effect, alveolar recruitment, greater humidification, and a better control of inspired

oxygen fraction [50]. The 'AIRVO' device (Fisher & Paykel http://www.fphcare.com/product-overview) is adaptable for children over a wide weight and age range and is capable of providing sustainable, non-invasive respiratory support generating high flows through a self-contained turbine-driven device (ACTRN12613000028707), without the need for specialized masks or endotracheal intubation – all of which are significant financial and technical barriers for use in resource-poor settings.

Trials and systematic reviews of oxygen delivery systems

A Cochrane review (update) conducted in 2014 identified four studies (479 participants) assessing the efficacy of non-invasive delivery methods for the treatment of pneumonia in children [24]. Three RCTs (399 participants) compared the effectiveness of nasal prongs or nasal cannula with nasopharyngeal catheter and one non-RCT (80 participants) compared head box, face mask, nasopharyngeal catheter (control) and nasal cannula. The use of a face mask showed a statistically significant lower risk of failure to achieve arterial oxygen > 60 mmHg than the nasopharyngeal catheter (one non-RCT; 80 participants; odds ratio (OR) 0.20, 95% CI 0.05 to 0.88). The use of a head box showed a non-statistically significant trend towards a reduced risk of treatment failure compared to the nasopharyngeal catheter (one non-RCT; OR 0.40, 95% CI 0.13 to 1.12). Nasal prongs are associated with fewer nasal obstruction problems. The quality of the evidence was judged to be very low.

Use of high flow to date has only been studied in paediatric populations outside of the developing world, where the use mechanical ventilation is incorporated in the treatment algorithm. A recent open 3-arm randomised trial comparing bubble-CPAP, high flow (with humidified air/oxygen mix by NPs) and standard (low flow) oxygen therapy in a planned sample size of 975 children (aged 0-4 years) in Dhaka with SP and hypoxaemia (SpO_2 <90%) was stopped by the external data and safety monitoring committee after the second interim analysis [51]. The primary outcome was treatment failure (composite of clinical, hypoxaemia SpO_2 <85% and blood gas parameters); secondary outcomes included mortality. Overall, 225 children were enrolled. The bubble-CPAP group had a lower risk of treatment failures compared with the low flow oxygen therapy group (relative risk [RR] 0.27, 99.7% CI 0.07-0.99, p=0.0026), but there was no difference with the high flow oxygen therapy group (RR 0.50, 99.7% 0.11-2.29 p=0.175). Children who received oxygen by bubble-CPAP had significantly lower rates of death than those receiving the low flow oxygen therapy (RR 0.25, 95% CI 0.07-0.89 p=0.022), but similarly there was no difference with those receiving high flow oxygen therapy (RR 0.30, 95% CI 0.09-1.05, p=0.082) [51].

While the authors concluded that oxygen therapy delivered by bubble-CPAP improved outcomes in children with VSP and hypoxaemia compared with standard low-flow oxygen therapy, an accompanying editorial indicated that firm conclusions cannot be drawn owing to the early stopping, as a result of incorrect statistical procedures, and indicated the need for further RCTs [52].

1.1 Rationale for current study

Justification for a controlled trial

The high incidence and mortality associated with hypoxia in children with pneumonia and severe illness at hospitals constitutes a major public health challenge. Options for supportive care in hospitals without access to mechanical ventilation are limited. At present, there is uncertainty as to what the best cut-off threshold for cost-effective oxygen delivery is and

whether the addition of high flow is safe and results in improved outcome. Since hypoxia is so common and treatment costs and practicalities for optimal delivery of oxygen are complex, the COAST trial will contribute substantial new knowledge in this area. The COAST trial was proposed and discussed at the launch meeting of ISARIC in Annecy, July 2012. The meeting brought together many leading researchers and clinical trialists in severe respiratory illnesses with representation from international agencies, such as the WHO. The proposal had wide support as it was anticipated that the trial would result in refinements to the current WHO recommendations and provide a robust evidence to support the targeting of oxygen to those with the greatest need and averting unnecessary consumption in financially constrained hospitals. The pragmatic nature of COAST and locations chosen will mean that the results of the trial will be immediately generalisable. The successful conduct of the FEAST Trial acts as a pilot for the conduct of COAST. The proposed high flow oxygen delivery device is in current use in South Africa, and feasibility testing and staff training in the COAST sites will occur before trial commencement.

The COAST trial was preceded by the Paediatric Oxygenation Strategies Treatment Study (POST), a feasibility study, which assessed feasibility of both the intervention and the RCT processes (including testing whether high flow oxygen via the AIRVO device can be used effectively in a hospital in Africa). The POST study helped inform the trial protocol for COAST.

Research gap: timeliness for a clinical trial
The international charity Lifebox (http://www.lifebox.org) launched a campaign to close the global ‘pulse oximetry gap’ (Make It Zero). As more hospitals acquire pulse oximeters, the hidden ‘hypoxia burden’ will be recognised and demands for an equitable supply of oxygen will increase. A recent commission from a collaboration between The Lancet and Imperial College, examining how medical technology could be used to improve health in low- and middle-income countries, identified technologies such as oxygen delivery as future priorities [53]. Nevertheless, important lessons were learnt in the FEAST Trial where fluid resuscitation, part of emergency care for decades in well-resourced countries, resulted in worse outcome in the fluid bolus arms [37]. Simple translation of established medical practice, largely based on a low quality of evidence, to resource-poor countries should be preceded by definitive trials to generate the evidence for best practice and cost-effectiveness. The COAST trial will provide the relevant evidence supporting such initiative.

Benefits
In conducting COAST, we will identify the hidden burden of hypoxia and therefore more children are likely to benefit than if the trial was not conducted in the first place. In FEAST, we witnessed substantial improvements in outcomes in the hospitals where the trial was taking place. Outcomes of participants in trials are often substantially improved. Extra clinical personnel, regular patient clinical assessment and the basic equipment for continuous patient monitoring will be available during the trial so that if the complications above were to arise they could be detected and treated. Pre-trial training will include sign recognition for these complications and training on treatment. Both these will be covered in detail in the Manual of Operations which will be available on the ward.

1.1.1 For the child

The direct benefits to the child and/or family (outlined in the Patient Information Sheet, Appendix C) include:

- The consent process, as in the FEAST Trial [54], will allow parents/guardians time to weigh up benefits and risks before giving deferred consent.
- Closer observation during the first 48 hours of admission, which, as a result, allows doctors and nurses to make important changes to the child's treatment during in-hospital admission.
- All routine non-trial medications required by the hospital to treat the child will be made available (when unavailable, parents/guardians have to resort to sourcing these privately).
- The parents/guardians of participating children will be asked to return for follow-up at 28 and 90 days after admission (only for those with suspected neurological sequelae at Day 28). Reimbursement for transport cost after discharge and for follow-up visits, plus any treatment costs required during the visits, will be made.

1.1.2 For the hospital

The direct benefits to the hospital include:

- Substantial investment in training and provision of equipment and consumables to provide oxygen will improve the treatment of all critically sick children.
- Pulse oximeters will routinely be used in triage, thus improving the specificity of the clinical criteria for oxygen-use – this can then be directed to those with hypoxia and reduce potential wastage of this resource to children who do not require oxygen therapy.
- Additional staff, employed directly for the trial, who are likely to manage the sickest patient (those at high risk of hypoxia and thus eligibility for the trial) will help relieve the treatment burden on acute paediatric services.

1.1.3 For health personnel

The direct benefits to health personnel are mainly professional development of the members of the trial teams and clinical teams for the purposes of running the trial, including basic life support courses, clinical trials training and research training.

Risks

Oxygen saturation threshold

Children will be carefully monitored, including active reporting of solicited adverse events (AEs). Training of trial staff, as in FEAST, will ensure that trial participants receive recommended standard of care and careful monitoring. Expected inherent hazards of the intervention strategies, solicited and serious adverse events (SAEs) as defined by ICH-GCP guidelines, are secondary outcomes of the trial; these will be reported in real time and assessed by an independent Endpoint Review Committee (ERC). We have included within the protocol oxygen for all children whose saturations are ever <80% to minimise harm. The ICH-GCP compliant SAE data collection and reporting procedures will be adopted. Clinical staff at sites will be trained by the Chief Investigator (ChI) and training team at the initiation visit to recognise expected side effects.

High flow versus low flow

High flow is an accepted strategy in the management of children with respiratory failure in a multitude of countries, with few reports that it is unacceptable, so the risks of harm from this strategy are known and are extremely low.

Deficiencies in oxygen supply

We will use bottled oxygen, whenever this is available. When supplies are low, we will use oxygen concentrators (at least three per site) and a reliable ‘power back’ system of electricity. The backup system will use a bank of batteries and charge during periods of electricity supply. If there are power outages, it will automatically switch on to provide backup power for up the 12 hours. If there are long periods of power outages (though unusual), then the trial teams will stop screening patients and not enrol into the trial until power is restored. A maximum of four patients per day will be recruited to alleviate potential burden on the available oxygen supplies so that this can be used in children outside of the trial (including cases where consent is declined).

2 Trial aim and objectives

The COAST trial will investigate what the best oxygen delivery strategies are to reduce in-hospital mortality and morbidity in African children with respiratory distress complicated by hypoxia (defined as SpO_2 <92%). This will be done by evaluating two linked components of oxygen delivery:

1. whether liberal oxygenation is superior to permissive hypoxia (current routine standard of care in hospitals not screening all admissions for hypoxia); and
2. whether high flow oxygen delivery is better than low flow oxygen delivery (current routine standard of care in hospitals with no access to mechanical ventilation).

Both the primary and secondary objectives will address what threshold to deliver oxygen (above a threshold of SpO_2 ≥80%).

2.1 Primary objectives

To establish whether:

- liberal oxygenation will decrease mortality compared with permissive hypoxia (usual care); and
- high flow oxygen delivery will decrease mortality compared with low flow oxygen delivery (usual care).

2.2 Secondary objectives

The secondary objectives of COAST are:

- to assess the effectiveness in the speed and success of recovery from the initial respiratory failure;
- to assess safety and quality of life;
- to determine long-term effects of the delivery strategies; and
- to identify whether the additional costs of each of the interventions are proportionate to the health benefits and to inform future widespread implementation in terms of value for money.

3 Trial outcome measures

3.1 Primary

The primary outcome measure is mortality at 48 hours post-randomisation.

3.2 Secondary

The secondary outcome measures are:

- Treatment failure at 48 hours (i.e. still receiving oxygen/respiratory support)
- Survival to 28 days
- Neurocognitive sequelae (de novo or worsening of existing neurological impairment) at 28 days
- Disability-free survival to 28 days
- Time to hypoxia (≥92%) resolution during initial hospital stay
- Length of initial hospital stay
- Re-admission to hospital by 28 days
- Anthropometric status by 28 days
- Resolution of neurocognitive sequelae at 90 days (for those with neurocognitive sequelae at 28 days *(see above)*)

3.3 Trial design

COAST is an open, multicentre, fractional factorial RCT of 4,200 participants, aged from 28 days to 12 years, admitted to hospital with respiratory distress complicated by hypoxia. Participants will be enrolled over a 30-month period and followed up to 28 days post-randomisation (and at 90 days for those with neurocognitive sequelae at 28 days).

4 Selection of sites

Five hospitals in two countries will participate:

- Kenya: KEMRI Wellcome Trust Programme, Kilifi County Hospital (KCH). The clinical programme at KCH is a well-established research centre on the coast of Kenya. It has been the site of several clinical studies on severe malaria including two large phase III trials (AQUAMAT and FEAST). Coast Provincial General Hospital (CPGH) is based in the second largest city in Kenya (Mombasa) and has conducted in collaboration with KWTRP a number of research studies and Phase III clinical trials in children admitted to CPGH.
- Uganda: Mulago National Referral Hospital. Mbale Regional Referral Hospital and Soroti Regional Referral Hospital. Mulago, Mbale and Soroti hospitals have annual admissions to the paediatric ward of 18,000, 20,000 and 8000 respectively. There has been considerable research capacity development in the last five years, with these three sites involved in the FEAST trial and currently conducting a large multicentre trial of transfusion trial (TRACT).

The five centres in Africa represent a spectrum of intensity of malaria transmission, from perennial and high (in Mbale and Soroti) to seasonal and meso-endmic (in Kampala, Mombasa and Kilifi). Mulago and CPGH are located within large urban areas, whereas the populations that utilise Mbale, Soroti and Kilifi are more typically rural; however Mbale has a few slum developments within the town.

Central grant funding will be available for participating sites. To participate in the trial, selected hospitals and principal investigators (PI) must fulfil the criteria defined below.

4.1 Site/Investigator inclusion criteria

The site/PI should:

1. Be qualified by education, training, and experience to assume responsibility for the proper conduct of the trial at their site and should provide evidence of qualifications through an up-to-date curriculum vitae (CV) and/or other relevant documentation requested by the Sponsor, the Research Ethics Committee (REC) or Institutional Review Boards (IRB) and/or the regulatory authority(ies).
2. Be aware of, and should comply with, the principles of ICH-GCP and the applicable regulatory requirements. A record of GCP training should be accessible for all trial staff at the site.
3. Have available or appoint an adequate number of qualified staff for the duration of the trial to conduct the trial properly and safely. Trial staff should be adequately informed about the protocol, trial equipment and their trial-related duties. The site should maintain a Delegation Log of appropriately qualified persons to whom the PI has delegated trial-related duties.
4. Be able to demonstrate a potential for recruiting the required number of participants and have sufficient time to conduct the trial as per protocol and complete the trial within the agreed trial period.
5. Permit monitoring and auditing by the Sponsor, and inspection by the appropriate regulatory authority(ies).

4.2 Documentation

The following documentation must be in place prior to a trial site being opened to recruitment:

- Investigator Statement – this verifies that the site is willing and able to comply with the requirements of the trial and will be signed by the site PI;
- Signature Form – this will be signed by site staff delegated trial responsibilities by the PI; and
- Delegation Log and staff contact details.

All completed documents should be sent to the KCTF. Up-to-date copies of the Delegation Log and contact details (with any changes to trial personnel and/or their responsibilities) should be sent to the KCTF and a copy should be stored in the Investigator Site File (ISF) at the site.

4.3 Activation

On receipt of all the documents, written confirmation will be sent to the PI, at which point the site may start to screen for eligible patients.

Once the site is activated, they should ensure:

- adherence to the most recent version of the protocol;
- appropriate recruitment and care for participants in the trial;
- timely data entry; and
- prompt notification of all AEs and protocol deviations to the trial team at the KCTF.

5 Selection of participants

5.1 Screening

The trial will run 24 hours/seven days per week. Patients will be assessed for eligibility at the point of hospital admission by the treating nurse/clinician. Established triage systems will identify potential patients who will be screened against the inclusion/exclusion criteria; a member of the trial team will carry out a rapid structured assessment of heart rate, oxygen saturation (pulse oximetry), respiratory rate, axillary temperature, blood pressure, markers of shock (capillary refill time, pulse volume and assessment of lower limb temperature) and severity (conscious level and respiratory distress). Entry criteria will be based on clinical assessment alone; it is anticipated that this process will take five minutes.

Infants and children aged between 28 days to 12 years who are potentially eligible for inclusion will be fast tracked to the trial team for full eligibility screening and assent/consent for enrolment. Children who do not meet the eligibility criteria at initial assessment will be re-screened for eligibility up to 24 hours post-hospital admission.

Sites will maintain a record of all patients who are screened for eligibility for COAST:

- Eligibility Screening Log – will record all potentially eligible patients (who fulfil the inclusion criteria, but meet one or more of the exclusion criteria); and
- Screening Form – will record all patients who meet the full eligibility criteria (with reasons for non-randomisation).

5.2 Inclusion criteria

- Aged between 28 days to 12 years
- History of respiratory illness (cough, upper respiratory tract symptom or any respiratory symptoms, e.g. rapid breathing or increase work of breathing)
- Hypoxia (pulse oximetry reading of SpO_2 <92% recorded in room air over 5 minutes)
  a. Plus suspected severe pneumonia informed by WHO guidelines, as below - Sign of respiratory distress (any one of):
     - severe lower chest wall in-drawing
     - use of auxiliary muscles
     - head nodding
     - inability to feed because of respiratory problems
  b. Suspected pneumonia
     - fast breathing:
       - age 2–11 months: ≥ 50/minute
       - age 1–5 years: ≥ 40/minute
       - age 5-12 years ≥ 30/minute
     - chest auscultation signs:
       - decreased breath sounds
       - bronchial breath sounds
       - crackles
       - abnormal vocal resonance (decreased over a pleural effusion or empyema, increased over lobar consolidation)
       - pleural rub
  c. Signs of pneumonia with a general danger sign:
     - inability to breastfeed or drink
     - lethargy or unconscious
     - convulsions

5.3 Exclusion criteria

- Known uncorrected cyanotic heart disease
- Assent/consent refusal by parent/carer
- Previously recruited to COAST
- Already received oxygen for this episode of illness (at another health facility)
- Known chronic lung disease (not including asthma)

6 Enrolment

6.1 Consent

Once eligibility has been confirmed, authorised trial staff will approach parents/guardians to invite their child to take part in the trial. An information sheet will be provided to the parent/guardian in their usual language containing details of the COAST trial (Appendix C). The sheet will be read aloud to those who are unable to read. The doctor/nurse will check that the information has been fully understood and parents/guardians will be encouraged to ask questions they may have about their child's participation in COAST.

Where possible, prospective written informed consent will be sought from parents/guardians who will then be asked to sign the Consent Form (Appendix D). If parents/guardians are unable to sign, a thumbprint will be taken in lieu of a signature.

If it is considered that the full consent process would significantly delay treatment allocation, and consequently be detrimental to the child's health, then emergency verbal assent, used in the FEAST and TRACT trials [54], will be sought from parents/guardians by the admitting medical team (see Section 7.2).

6.2 Emergency verbal assent followed by deferred consent

It is likely that, due to the emergency nature of the patients' condition, the full consent process may delay treatment. If this is the case, then parents/guardians will be provided with a brief verbal description of the trial and will be given the opportunity to "opt out" of clinical research. Full consent will be sought once the child's clinical condition has been stabilised. The clinician will later sign the Verbal Assent Form (Appendix E) which will be filed with the Consent Form. If consent is withdrawn later, trial data collected up to the time of withdrawal will only be used and no further trial specific procedures will be conducted (see Section 7.5).

A copy of the Consent Form will be given to the parent/guardian, the original placed in the patient's medical notes, and a copy kept in the Investigator Site File.

6.3 Randomisation

The trial has two strata: **COAST A**: severe hypoxia, SpO_2 <80%); and **COAST B**: hypoxia (SpO_2 ≥80% and <92%).

Children in COAST A (2-arm, 1:1 ratio) will all receive oxygen and randomisation will allocate participants to one of two methods of oxygen delivery:

i. high flow oxygen delivery
ii. low flow (usual practice) oxygen delivery

Children in COAST B (3-arm, 2:1:1 ratio) will be allocated to

i. permissive hypoxia (no immediate oxygen); control
ii. high flow oxygen delivery
iii. low flow (usual practice) oxygen delivery

Participants will be allocated to treatment arms by a computer-generated list using random permuted blocks. The blocks will be stratified by trial site and baseline SpO_2 <80% or ≥80%.

The Trial Statistician at the ICNARC Clinical Trials Unit (CTU) will prepare the randomisation lists before the trial commences and kept at the ICNARC CTU, London. Opaque and sealed randomisation envelopes will be prepared and provided to each site, with one set for COAST A (SpO_2 <80%) and one set for COAST B (SpO_2 ≥80% and <90%). The envelopes for each site will be numbered consecutively and opened in numerical order. These will contain details of the treatment arms once opened.

This system has worked well in the emergency care trials, i.e. FEAST and TRACT (transfusion trial). To facilitate protocol adherence, a maximum per site will be agreed upon (for example up to 4 children per day) will be enrolled per site. This approach was very successful with respect to protocol adherence and the quality of data generated in the FEAST trial.

6.4 Co-enrolment

Participants will not be permitted to co-enrol in any other interventional studies while on COAST. Participation in studies that do not involve an intervention (e.g. observational studies) are acceptable, but should be discussed with the COAST TMG. The TMG will consider co-enrolment of COAST participants onto other observational studies where the management does not conflict with the COAST objectives on a case-by-case basis.

6.5 Withdrawal

In consenting to the trial, parents/guardians and their children are consenting to trial treatment, assessments, data collection and follow-up. However, children or their parents/guardians can withdraw from COAST at any time during the trial.

If a child, or their parents/guardians, chooses to withdraw any part of their trial treatment, they should always be followed-up and encouraged not to leave the full trial. The treating clinician remains free to give alternative treatment to that specified in the protocol at any stage if he/she feels it is in the participant's best interest, but the reasons for doing so should be recorded. In these cases, the participants will remain within the trial, and all data collected up to the point of withdrawal will be retained for the purposes of follow-up and primary outcome data collected and included in the trial analysis.

If a child, or their parents/guardians, no longer wishes to take part or contribute further data to the trial (e.g. follow-up), their decision must be respected. The Withdrawal Form (Appendix F) should be completed and sent to KCTF. Withdrawal of the participant should be recorded in their medical notes and no further data will be collected.

All identifiers will be removed at the end of the trial to ensure anonymity for all participants recruited into COAST.

7 Trial treatment

7.1 Randomisation allocation and treatment arms

The trial has two strata: **COAST A**: severe hypoxia, SpO_2 <80%); and **COAST B**: hypoxia (SpO_2 ≥80% and <92%). Patients will be assessed on severity of their hypoxia (SpO_2 <92% initially recorded over 5 minutes in children breathing room air) and assigned to their severity stratum (Figure 5).

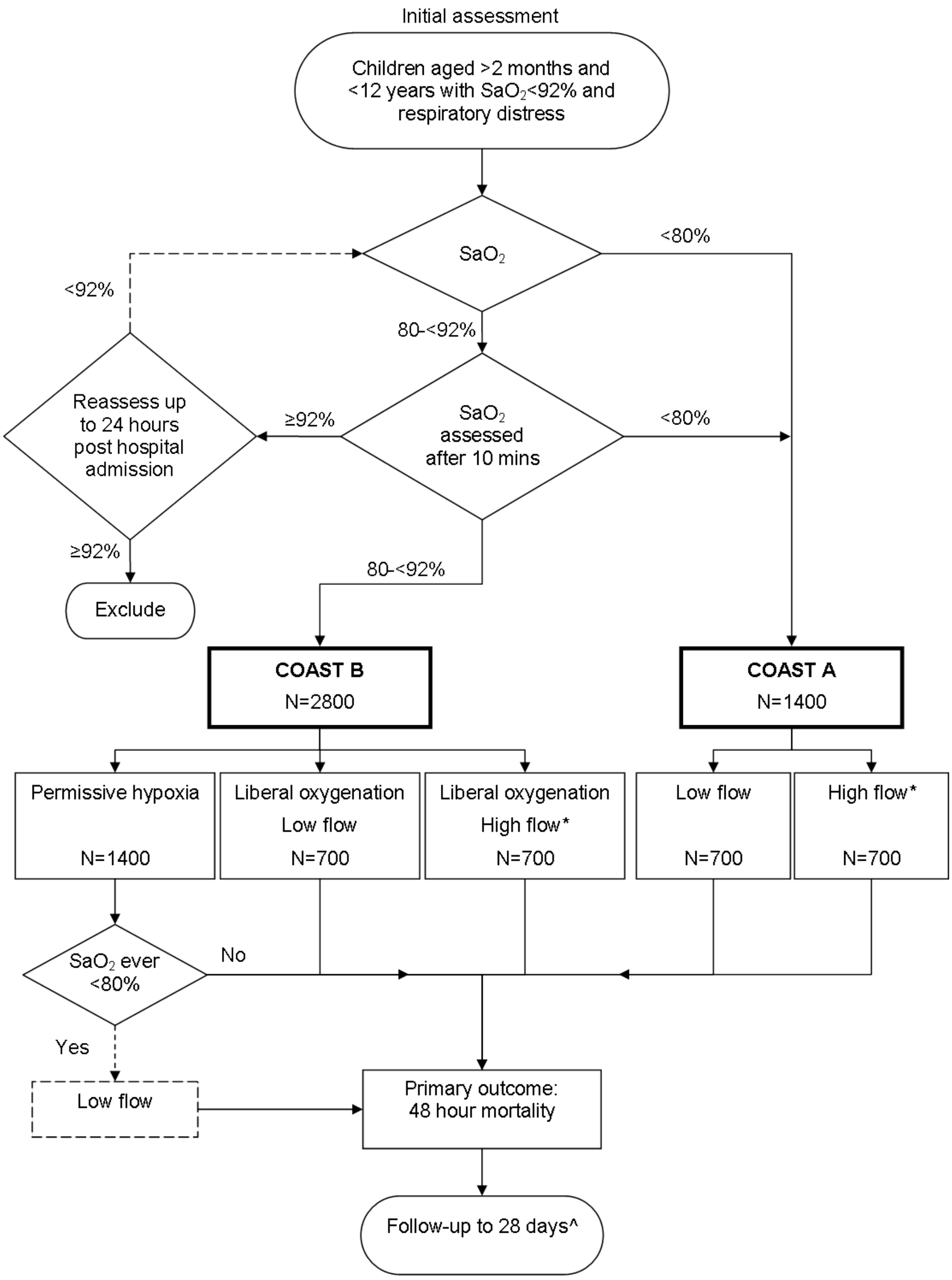

*Maximum of 48 hours

^Follow-up to 90 days for those with evidence of neurological sequelae at 28 days

Figure 5 Trial Flow

If baseline SpO_2 is below 80% (severe hypoxia), patients will be enrolled into COAST A (Figure 6).

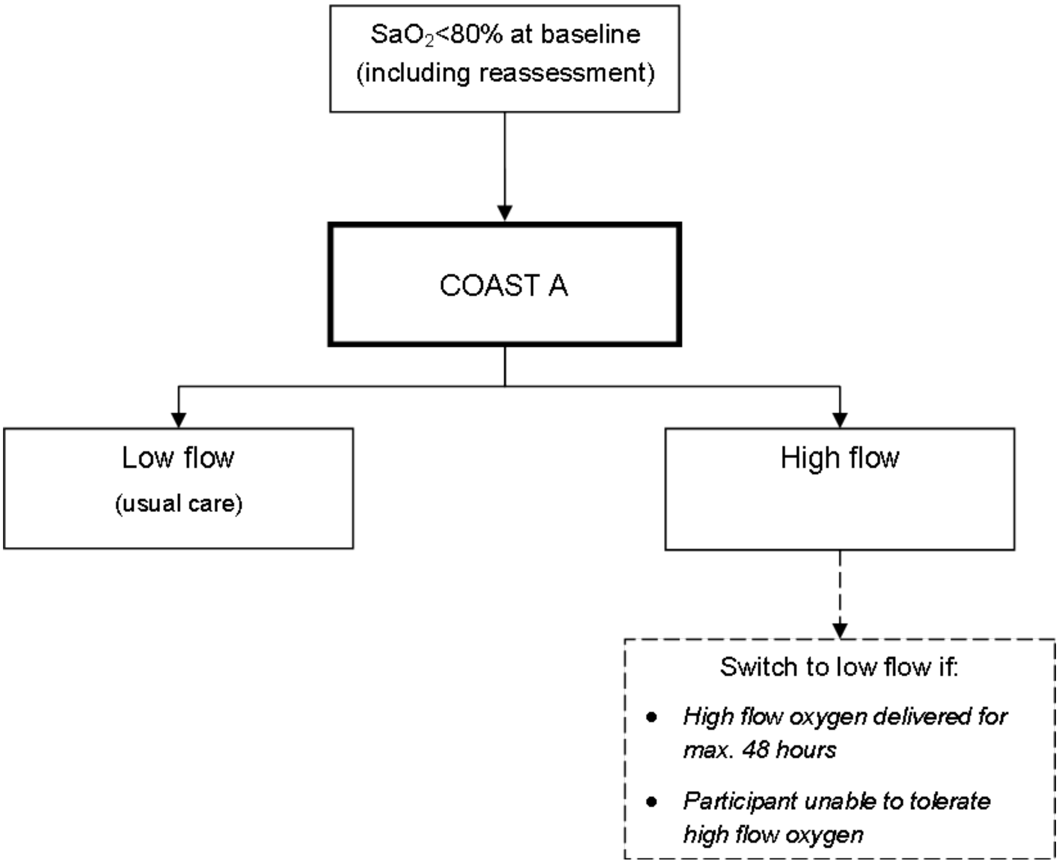

Figure 6 COAST A: Treatment arms

If baseline SpO_2 is between 80-91% which remains for 10 minutes (ie an additional 5 minutes to initial screening) in children breathing room air (in order to mitigate against the possibility of a transient hypoxia or development of severe hypoxia), patients will be enrolled into COAST B (Figure 7).

For children after 10 minutes reassessment, SpO_2:

- drops below 80%, patients will be eligible for COAST A and randomised **accordingly**;
- if ≥92% at admission patients will remain eligible for up to 24 hours post hospital admission (so long as the patient has not received oxygen). After 24 hours patients are no longer eligible, irrespective of SpO_2.

The reassessments conducted within the 24-hour time window will prevent recruiting patients with only transient episodes of hypoxia (e.g. accompanying hypoglycaemia or convulsions which resolved on appropriate treatment) and allow for recruitment of children who subsequently develop hypoxia early into their hospital admission.

Usual emergency care will be provided as part of standard clinical management.

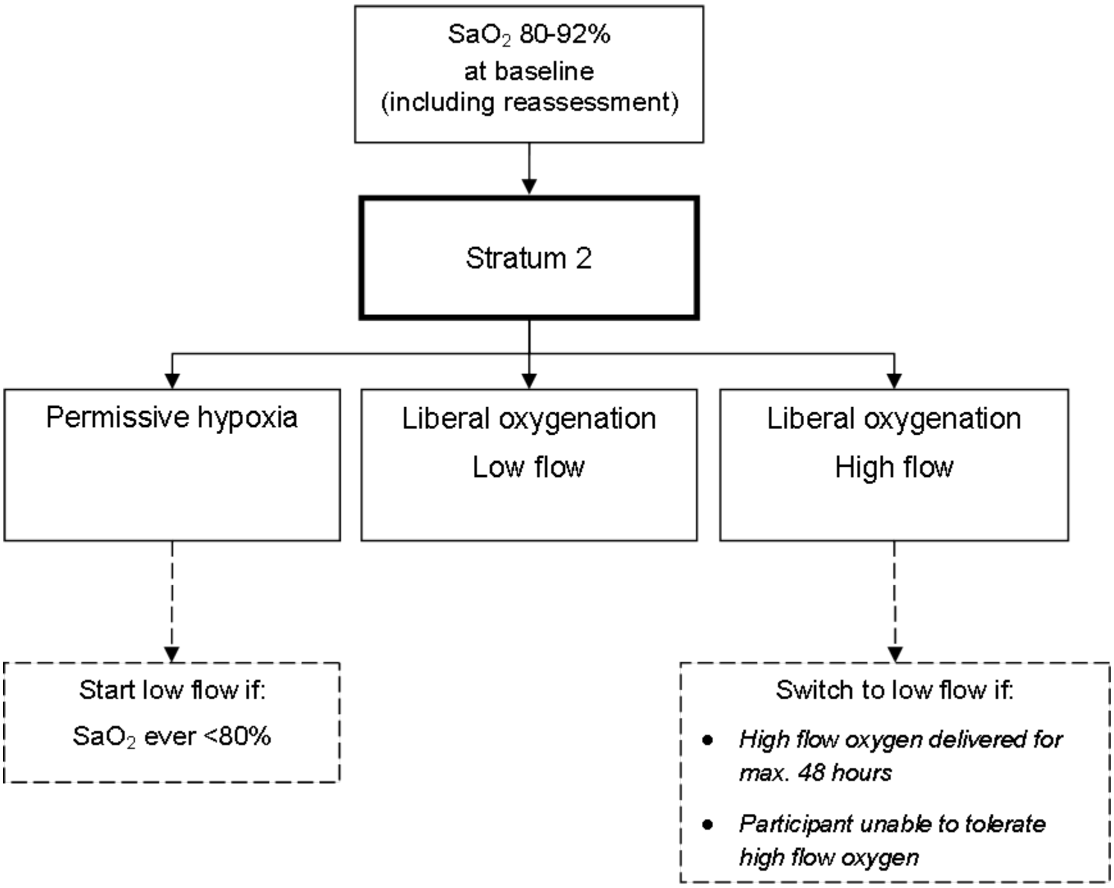

Figure 7 COAST B: Treatment arms

Trial Treatments:

i. Low flow oxygen (see Glossary for flow rate definition) until oxygen saturation ≥92% in room air
ii. High flow oxygen (see Glossary for flow rate definition) until oxygen saturation ≥92% in room air, or *the participant will switch to low flow oxygen (usual care) if:*
    - *High flow oxygen delivered for a maximum of 48 hours; if oxygen is still required at this point then the child will be switched to oxygen delivery by low flow (i.e. standard of care)*
    - *Unable to tolerate high flow oxygen provided indices of ventilation are optimal*

Extra clinical personnel, regular patient clinical assessment and the basic equipment for continuous patient monitoring will be available during the trial so that if complications were to arise they could be detected early and treated. Pre-trial training will include sign recognition for these complications and training delivery of treatment.

All trial participants will receive standard of care including antibiotics (intravenous or oral) anti-malarial drugs following national guidelines, based on WHO syndromic patient management [55].

7.2 Duration of treatment

The trial treatment period will last for a maximum of 48 hours post randomisation. After this time point, the participant will switch to usual care (standard clinical management).

For children receiving oxygen delivered by high flow oxygen, at 48 hours, if oxygen is still required (i.e. failure to wean into room air) at this point, then the child will be switched to oxygen delivery by low flow (i.e. standard of care).

During the 0-48 hour period, if a child is unable to tolerate high flow oxygen and if oxygen is still required (i.e. failure to wean into room air), then the child will be switched to oxygen delivery by low flow (i.e. standard of care).

If oxygen is discontinued before 48 hours e.g. hypoxia is resolved (SpO_2 ≥92% measured continuously over 30 minutes [17]), then they will switch to usual care (standard clinical management) following a successful trial of oxygen weaning.

Once weaning has occurred further clinical and bedside monitoring will occur twice daily until hospital discharge.

7.2.1 Standard clinical management

All other care will be determined by the clinical team primarily responsible for the participant's care.

7.3 Trial equipment

Reliable sources of oxygen and bedside equipment for patient monitoring will be provided for the duration of the trial.

High flow oxygen will be delivered by the AIRVO device; AIRVO is manufactured by industry leaders Fisher & Paykel Healthcare (http://www.fphcare.com/products/airvo/). The device, complete with consumables, will be provided to each participating site. The AIRVO device can be used from bottled oxygen and has an inflow for concentrator-delivered supplemental oxygen. The device is already adaptable for children over a wide weight and age range and is capable of providing sustainable, non-invasive respiratory support generating high flows through a self-contained turbine-driven device. Medical and nursing staff will be able to operate the machine with minimal training. Fisher & Paykel will also provide technical support during the trial. The AIRVO device will only be used for patients randomised to R2 to receive high flow oxygen delivery.

In addition, oxygen saturation measurement will be undertaken by pulse oximeters. Up to 10 ten pulse oximeters will be provided per site. The pulse oximeter is a handheld device for use in the operating room, recovery unit and ward areas. It is both battery powered (3 AA alkaline batteries) or mains powered (nickel-metal hydride rechargeable battery <u>or</u> a lithium ion rechargeable battery). The standard battery use is approximately 14 hours.

8 Data collection

Table 2 Participant data collection schedule

	TRIAL PERIOD											
	Enrolment and Allocation	Post-allocation										
TIMEPOINT	*Day of admission*	Hour 0	*Hour 1*	*Hour 2*	*Hour 4*	*Hour 8*	*Hour 12*	*Hour 24*	*Hour 36*	*Hour 48*	*Hospital discharge*	*Day 28*
ENROLMENT:												
Eligibility screening	X											
Informed consent	X*											
Allocation		X										
ASSESSMENTS:												
Baseline data	X	X										
Diagnostic investigations	X											X
Laboratory/point of care diagnostics	X											

	TRIAL PERIOD											
	Enrolment and Allocation	Post-allocation										
TIMEPOINT	*Day of admission*	Hour 0	*Hour 1*	*Hour 2*	*Hour 4*	*Hour 8*	*Hour 12*	*Hour 24*	*Hour 36*	*Hour 48*	*Hospital discharge*	*Day 28*
Hospital stay data		X	X	X	X	X	X	X		X	X	
Primary outcome										X		
Secondary outcomes										X	X	X
Cost-effectiveness outcomes											X	X

*as soon as feasible

Timepoint	Admission	Hour 0	Hour 1	Hour 2	Hour 4	Hour 8	Hour 12	Hour 24	Hour 36	Hour 48	Hospital discharge	Day
Primary outcome										X		
Secondary outcomes										X	X	X
Cost-effectiveness outcomes											X	X

8.1 Data collection – participants

8.1.1 Baseline – characteristics

Following consent and randomisation(s), data will be collected on:

- date and time of hospital admission
- eligibility criteria
- date and time of consent
- patient identifiers
- patient sociodemographics
- oxygen saturation level
- pre-existing neurocognitive deficits
- hospital admission diagnosis and category
- suspected cause of respiratory distress
- severity of dyspnoea (assessed using an age-adjusted integrated respiratory distress and wheeze score [56])
- co-morbidities
- severity of illness score
- child's ability to tolerate oxygen using an analogue scale [57]

8.1.2 Baseline – laboratory tests

Admission blood samples will be taken for the following investigations:

- full blood count, urea and electrolytes
- lactate
- glucose
- malaria blood slide and/or malaria RDT
- white cell and red cell pellet (for subsequent DNA extraction)
- blood culture (where culture facilities are available)
- nasal and oropharyngeal swabs and saliva specimen (for pathogen diagnosis)
- plasma storage (acute serology; markers of immune markers)
- EDTA x 2: for haemoglobinopathies and molecular pathogen diagnostics

Chest x-rays will also be taken when the patient is stable. In accordance with national guidelines, HIV testing will be performed after admission procedures are complete and assent given by parents or guardians. Pre- and post-test counselling will be done in accordance to routine practice.

8.1.3 Hospital stay

All participants will be reassessed clinically at 1, 2, 4, 8, 12, 24, 36 and 48 hours post-randomisation, and twice daily thereafter until discharged from hospital. At each review, the following will be recorded:

- conscious level
- vital signs (heart rate, oxygen saturation and percentage, respiratory rate, axillary temperature, blood pressure)
- severity of dyspnoea (assessed using an age-adjusted integrated respiratory distress and wheeze score [56])

- oxygen received and delivery method
- prescribed drugs received
- volume and type of intravenous fluid (including blood transfusion) per 24 hours
- AEs

8.1.4 Hospital stay – additional laboratory tests

Additional blood samples will be taken at the discretion of the clinical team at other times during the hospital stay if deemed necessary for a clinical deterioration for the following investigations:

- haemoglobin
- blood glucose
- lactate

8.1.5 Hospital discharge

- final diagnosis
- discharge status
- discharge date and time

All clinical staff members must follow all national and international guidance on hospital discharge tests and procedures [17].

Children, or their parents/guardians, will receive a follow-up clinic visit invitation on a card; the date will be 28 days after the day of randomisation. Transport costs after discharge and for the follow-up visit will be reimbursed to parents/guardians by the trial site coordinator. Children who are still an in-patient at 28 days will be followed up in hospital.

8.1.6 Follow-up

A symptom checklist and targeted physical examination will be performed at the clinic visit scheduled for 28 days after the day of randomisation.

- Medical history since last visit, including hospital re-admissions, transfusions and SAEs
- Convalescent sampling (plasma)
- Neurocognitive function assessment (using the Developmental Milestones Checklist and an adaptation of the Kilifi Developmental Inventory [58, 59])

Any participant lost to follow-up before 28 days will be traced for vital status (using locator data and mobile telephone contacts taken prior to discharge).

Participants with evidence of neurocognitive sequelae at 28 days will also be followed up again at 90 days.

8.1.7 Health economics

The trial will measure healthcare-related costs for the trial participants, starting at randomisation and continuing for the duration of follow-up.

- Costs incurred by the participants and their families (e.g. transport, indirect and companion person's costs)

- Information on hospitalisation (e.g. number, reason, and duration of stay)
- Data on other healthcare resource utilisation (e.g. outpatient visits, medications, and procedures)

8.1.8 Outcomes

Primary outcome

Survival status will be recorded at 48 hours post-randomisation. Any participants lost to follow-up before 48 hours, without withdrawing consent, will be traced for vital status.

Secondary outcomes

- Treatment failure will be defined as continued SpO_2<92% in the presence of respiratory distress (indrawing or intercostal retractions) at 48 hours post-randomisation.
- Survival status will be recorded at 28 days post-randomisation. Any participants lost to follow-up before 28 days, without withdrawing consent, will be traced for vital status.
- Neurocognitive sequelae will be defined as the development of a new neurocognitive deficit, assessed using the modified Kilifi Developmental Index, between the day of admission and 28 days following randomisation.
- Composite disability-free survival will be defined as a change to disability-free status, between the day of admission and 28 days following randomisation.
- Time to hypoxia resolution will be defined as the duration in hours from randomisation to hypoxia resolution, defined as SpO_2 ≥92% measured continuously over a period of 30 minutes during initial hospital stay.
- Duration of respiratory support will be defined as the number of days alive and free from receipt of administered oxygen during the first 28 days following randomisation.
- Length of initial hospital stay will be defined as the duration in days from randomisation to initial hospital discharge.
- Re-admission to hospital will be defined as hospitalisation (at the trial centre or other health facility) for acute illness (i.e. not due to an existing illness or an elective admission) between discharge and 28 days following randomisation.
- Anthropometric status will be defined by Z-scores of height, weight and mid-upper arm circumference and any evidence of visible severe wasting or signs of kwashiorkor.
- Resolution of neurocognitive sequelae will be defined as the full resolution by Day 90 of any neurocognitive deficit reported at Day 28.
- Resource use and costs will be assessed using the methods previously described (see Section 8.1.7) at 28 days following randomisation.

8.2 Data collection – site staff

8.2.1 Nurses

Nurses will be responsible for collecting data collected at each clinical review during the hospital stay.

8.2.2 Doctors

During the initial hospital stay, a doctor will review the participant routinely (at the time points listed above) using a symptom checklist and targeted physical examination (to evaluate any reported symptoms). Additional reviews will be done where clinically indicated and recorded in the case notes.

8.2.3 Trial site coordinator

The trial site coordinator will be responsible for completing the Case Report Forms (CRF), following up any data queries and tracing the results of pending laboratory tests.

9 Data management

Each site will be responsible for its own data entry using the COAST CRF. After 48 hours, the hospital patient's notes will be used in place of the CRF; a COAST trial number sticker will be added on the front page of the patient's hospital notes and a caption "COAST trial participant: Notes to be reviewed at discharge" written below the sticker. Additional pages in the COAST CRF will be available to report key clinical, laboratory or AEs and bi-daily observations during the period prior to discharge.

Following on-site monitoring, data will be entered onto a web-enabled trial database directly at the site. The site will retain the original paper CRFs. All clinical and laboratory data will be recorded in the CRF and stored with a unique trial number identifier. All data will be regularly backed up and backup copies will be stored securely both on and off site.

Data will be checked and undergo validation checks for completeness, accuracy and consistency of data. Data queries that arise from these checks will be sent from KCTF to the trial site coordinators. The trial site coordinator is required to ensure that queries are resolved as soon as possible, including updating the relevant paper CRFs and the trial database as required. The KCTF will send reminders for any overdue data or outstanding queries. Following validation, data will be remotely extracted by ICNARC CTU once a month to generate progress reports for each site.

Ongoing data entry and validation and adherence to the trial protocol at sites will be closely monitored by KCTF; any concerns will be raised with the site PI.

9.1 Sample storage

All samples for storage (including those taken during hospital stay and those taken for research purposes, if consented to) will require no more than 10 ml of venous blood (varies by age). Any blood taken for research purposes under emergency deferred consent from children whose parents subsequently refuse consent will be confidentially destroyed.

10 Safety monitoring

10.1 Definitions

The following definitions have been adapted from Directive 2001/20/EC of the European Parliament (Clinical Trials Directive) [60] and ICH-GCP guidelines (E6(R1), 1996).

Adverse Event (AE) – Any untoward medical occurrence or effect in a patient participating in a trial, which does not necessarily have a causal relationship with the trial treatment. An AE can therefore be any unfavourable symptom or disease temporally associated with the use of the trial treatment, whether or not it is related to the allocated trial treatment.

Serious Adverse Event (SAE) – An AE is defined as serious if it:

- results in death;
- is life-threatening;
- requires inpatient hospitalisation or prolongation of existing hospitalisation;
- results in persistent or significant disability/incapacity; or
- is a congenital anomaly/birth defect.

Medical judgement should be exercised in deciding whether an SAE is serious in other situations. Important SAEs that are not immediately life-threatening, do not result in death or hospitalisation but may jeopardise the subject or require intervention to prevent one or any of the other outcomes listed in the definition above should also be considered as serious.

Life threatening in the definition of an SAE refers to an event in which the subject was at risk of death at the time of the event. It does not refer to an event that hypothetically might have caused death if it were more severe.

Unexpected and Related Serious Adverse Event - A suspected AE related to the treatment that is both unexpected (i.e. not consistent with the expected outcomes of the treatment being offered) and serious.

N.B. Suspected Unexpected Serious Adverse Reaction (SUSAR) will not be assessed in this trial as it falls outside the scope of the European Union Clinical Trial Directive.

10.2 Severity

The assessment of severity will be graded based on the PI's or a delegated local investigator's clinical judgement using the following definitions:

- None: indicates no event or complication.
- Mild: complication results in only temporary harm and does not require clinical treatment.
- Moderate: complication requires clinical treatment but does not result in significant prolongation of hospital stay. Does not usually result in permanent harm and where this does occur the harm does not cause functional limitation to the participant.
- Severe: complication requires clinical treatment and results in significant prolongation of hospital stay and/or permanent functional limitation.
- Life threatening: complication may lead to death.
- Fatal: indicates that the participant died as a direct result of the complication/AE.

10.3 Relatedness

The PI or designee should use clinical judgement to determine the relationship between the trial treatment and the occurrence of each AE using the following definitions:

- None: there is no evidence of any relationship to the trial treatment.

- Unlikely: there is little evidence to suggest a relationship to the trial treatment, and there is another reasonable explanation of the event.
- Possibly: there is some evidence to suggest a relationship to the trial treatment, although the influence of other factors may have contributed to the event.
- Probably: there is probable evidence to suggest a relationship to the trial treatment, and the influence of other factors is unlikely.
- Definitely: there is clear evidence to suggest a relationship to the trial treatment, and other possible contributing factors can be ruled out.

10.4 Expectedness

The PI or designee must assess the expectedness for each SAE regardless of its relationship to the trial procedures.

- Expected: the event is listed as an expected AE in Appendix B.
- Unexpected: the event is not listed as an expected AE in Appendix B.

10.5 Exempt adverse events

All patients eligible for COAST are critically ill and due to the complexity of their condition are at increased risk of experiencing AEs. Many of these events are expected as a result of the patient's medical condition and standard treatment received in hospital, but may not be related to participation in the trial. Consequently, any unexpected AEs occurring as a result of the patient's medical condition or standard hospital treatment will not be reported. Pre-existing conditions do not qualify as AEs unless they worsen, but should be documented in the patient's medical notes.

10.6 Recording and reporting

All AEs that occur between randomisation and 28 days post-randomisation must be recorded in the patient medical notes, on the COAST paper CRFs and on the web-enabled trial database. Information regarding date and time of event onset, severity and relatedness of the AE to trial treatment must be recorded.

At each clinical review the clinician or nurse will check for potential SAEs, grade 3 or 4 AEs and solicited AEs. SAEs will be reported to KCTF using an SAE form; the form should be completed, scanned and sent electronically within 24 hours of becoming aware of the event. The clinician or nurse should record the nature of event, date of onset, severity, corrective therapies given, outcome, relatedness and expectedness on the SAE form, and the PI or designee should sign it off prior to sending. Sites should also report all SAEs as required by their local REC and/or local policies. Any questions concerning AE recording and reporting should be directed to KCTF in the first instance via email or by telephone.

All SAEs must be followed-up until resolution. The PI or designee must provide a follow-up SAE report(s) within five days of the initial report if the SAE was not resolved at the time the initial report was submitted.

10.7 Central processing

SAEs will be reviewed immediately by a designated physician (SAE reviewer) in the KCTF and periodically by the ERC. If the event is evaluated by either the PI or the SAE reviewer as

an unexpected and related SAE, the KCTF will submit a report to the appropriate ethics committees within 15 calendar days.

The KCTF will provide safety information to the ChI, TMG, Trial Steering Committee (TSC) and Data Monitoring Committee (DMC) for review on a regular basis (as deemed necessary).

10.8 Additional monitoring and notification process

The KCTF will also monitor data for documented AEs that are not considered to be related to the trial treatment. In the event that any trial procedure does appear to be resulting in AEs, the ChI and/or TMG will be contacted for their opinion. If it is declared necessary to review the conduct of the trial, the KCTF will inform the appropriate ethics committees as necessary.

11 Trial monitoring and oversight

11.1 Site

This trial will be monitored according to a Monitoring and Quality Management Plan which will set out the frequency of visits, the degree of source document verification against the CRFs and the requirements for triggered on-site monitoring visits. Monitoring will start with 100% source document verification, then will be reviewed for each site once a satisfactory and sustained performance in quality assurance is established. All monitoring will adhere to the ICH-GCP guidelines (E6(R1), 1996).

A detailed site initiation visit (SIV) will be performed at each site by staff from KCTF. The SIV will include training in the trial procedures, such as delivering the trial treatments, reporting guidelines for AEs and data collection and management. All staff at sites involved in the trial will receive formal training in GCP through a dedicated training programme during the SIV and through an on-line course.

Local monitoring teams, responsible to KCTF, will oversee the standards and quality of the trial at each site. All monitors will be appropriately qualified and trained.

At each monitoring visit, monitors will:

- verify completeness of Investigator Site File;
- assess for any non-adherence to protocol;
- review eligibility verification and consent procedures;
- look for missed AE recording/reporting;
- verify completeness, consistency and accuracy of data being entered on CRFs; and
- provide additional training as needed.

The monitors will require access to all patient medical records including, but not limited to, laboratory test results and prescriptions. The PI or delegated local investigator should work with the monitor to ensure that any problems detected are resolved.

11.2 Site Trial Management Team

Each site will have a PI, who will be overall responsible for the day-to-day management of the trial, and will employ a clinical team to coordinate triage and identification of eligible participants and administration of trial interventions. Additional clinical nurses will be responsible for clinical reviews and follow-up. A clinical nurse coordinator will manage the trial team and integrate the trial within routine clinical services and patient care.

Trial site coordinators are responsible for ensuring completeness of trial documents, SAE reporting and interactions with monitors and KCTF. Support will be provided for laboratory staff and ward assistants to facilitate blood tests, sample preparation and storage. At each site, dedicated COAST administrators will manage trial operations and human resource issues.

Overall trial management, training and monitoring and data management will be coordinated from the KCTF, based in Kenya. The ICNARC CTU, based in London, will oversee trial management by teleconferences and face-to-face meetings with the TMG.

11.3 Trial Management Group

The TMG will be comprised of the ChI, site PIs and co-investigators. The TMG will meet approximately once a year in-person and will hold regular monthly teleconferences to discuss general trial matters. In addition, the site PIs will summarise their progress and discuss any challenges and difficulties at their site. All decisions regarding the overall running of the trial will be made in this forum, with the exception of matters of fundamental importance to the viability of the trial or that require major changes to the protocol – these will be referred to the TSC.

11.4 Trial Steering Committee

The progress of the trial will be monitored and supervised by the TSC. It will meet approximately once a year. The TSC will compose of an independent chair and a majority (60%) of independent members. Non-independent members will include the ChI and representatives from the co-investigators and from each participating country. Representatives from the Funder and Sponsor will be invited to the meetings as observers.

11.5 Data Monitoring Committee

An independent DMC will be set up to review data on enrolment, safety, adherence to the trial protocol and efficacy at regular intervals and in strict confidence. It will meet approximately once a year. The DMC will make recommendations to the TSC as to the continuation of the trial. The DMC will be comprised of a chair and three other independent members.

11.6 Endpoint Review Committee

An ERC will be set up to review clinical data and will determine the validity of the endpoints. The ERC will adjudicate endpoints blinded to randomised allocations; relationship to all possible trial treatments (liberal oxygenation, permissive hypoxia and high and low flow oxygen delivery) will be solicited to avoid unblinding. The ERC will be made up of an independent chair and will include the PIs from each site as well as other independent clinicians.

12 Trial Closure

12.1 End of trial

The end of the trial will be when the final participant has completed their 28-day follow-up (or 90-day follow-up if applicable). The Declaration of End of Trial Form will then be submitted to the relevant ethics committee(s), as required.

12.2 Archiving

At the end of the trial, the KCTF will securely archive all central and site essential trial-related documentation and samples taken for a minimum of 10 years, in line with Medical Research Council (MRC) guidance on Good Research Practice [61], and thereafter confidentially destroyed. All archived documents must be available for inspection and monitoring by appropriate authorities upon request.

12.3 Early discontinuation

Should a site choose to close to participant recruitment before the end of the trial, the PI must inform the KCTF in writing. Follow-up, as per the trial protocol, must continue for all participants already recruited into the trial at that site. Sites that contravene the trial protocol and the Site Agreement will be subject to review by the TMG and Sponsor.

The trial may be stopped early by the TSC. In which case, sites will be informed in writing by the KCTF of the reasons for early closure and the actions to be taken as regards the treatment of participants. All randomised participants will continue to be followed up as per the trial protocol.

13 Statistics

A Trial Statistician will be based at ICNARC CTU, under the supervision of Dr David Harrison (Senior Statistician, ICNARC CTU), and will provide statistical support for DMC analyses.

13.1 Sample size calculation

Sample size calculations were informed by identifying patients meeting the proposed COAST inclusion criteria within two datasets: the FEAST trial (n=873/3170; 28%); and the Kilifi Hospital, Kenya admission cohort (n=2609/36,621; 7%). Based on these data, it was estimated that two thirds of eligible children would present with SpO_2 ≥80% (FEAST 63%; Kilifi 69%). Baseline 48-hour mortality for children receiving low flow oxygen was assumed to be 9% for children with SpO_2 ≥80% (FEAST 10%; Kilifi 9%) and 26% with SpO_2<80% (FEAST 30%; Kilifi 26%). Due to the complex nature of the design, power calculations were undertaken by simulating datasets under the assumed alternative hypotheses and calculating the proportion of simulated datasets in which a significant effect (P<0.05) was detected for each of the two comparisons [62]. Based on these simulations, a total sample size of 4,200 children would give 90% power to detect a clinically relevant difference of a 33% RR reduction associated with liberal oxygenation compared with permissive hypoxia, and a clinically relevant difference of a 25% RR reduction for high flow compared with low flow oxygen delivery.

The sample size calculation is based on the primary outcome of mortality at 48 hours with no losses to follow-up expected. Losses to follow-up are anticipated to increase to 2% at 28 days post-randomisation, based on data from the FEAST trial [37].

13.2 Analysis plan

The analyses will be described in detail in a full Statistical Analysis Plan. This section summarises the main issues.

All analyses will be undertaken using the intention-to-treat principle with a two-sided P-value of P<0.05 taken to indicate statistical significance.

The primary outcome (48-hour mortality) will be analysed as a binary outcome using logistic regression including both treatment allocation variables simultaneously and adjusted for the stratifying factors (baseline SpO_2 and trial site).

Secondary outcomes will be analysed using generalised linear models, with the same model structure as for the primary outcome above, as follows:

- Treatment failure at 48 hours, re-admission to hospital by 28 days, neurocognitive sequelae at 28 days and disability-free survival to 28 days will be analysed as binary outcomes using logistic regression, with all deaths included in the failure group.
- Time to hypoxia resolution will be analysed as a time-to-event outcome using a Fine and Gray competing risks model to account for the competing risk of mortality.
- Length of initial hospital stay, days alive and free from receipt of oxygen and anthropometric status will be analysed as continuous outcomes using linear regression.

- Survival to 28 days will be analysed as a time-to-event outcome using a Cox proportional hazards model.
- Resolution of neurocognitive sequelae at 90 days will be analysed as a binary outcome using logistic regression, only among those with neurocognitive sequelae at 28 days.

Pre-specified subgroup analyses will assess whether the effects of the two interventions vary according to categories of baseline SpO_2 (<80%, 80-84.9%, 85-89.9%, 90-91.9%) and by trial site. Additional hypothesis-generating analyses will investigate whether there is any evidence for a different impact of the interventions according to the following categorical variables: fever; malaria; microbiological evidence of sepsis (blood culture or retrospective molecular diagnosis); radiographic evidence of pneumonia; HIV; severe anaemia (haemoglobin <5g/dl); and undiagnosed sickle cell disease. Subgroup analyses will be conducted by testing the significance of interaction terms in the regression models as specified above.

13.3 Interim analysis

The DMC will meet to review unblinded data for at least 525, 1575 and 2625 participants (corresponding to 3 months, 9 months and 15 months at the anticipated recruitment rate). Guidelines to recommend early termination will be based on a Peto-Haybittle stopping rule (P<0.001). A recommendation to discontinue recruitment, in all participants or in selected subgroups, will be made only if the results are likely to convince the general clinical community and participants in COAST.

13.4 Ancillary Studies

a. Economic and cost-effectiveness evaluation

The cost-effectiveness of oxygen delivery strategies will be estimated by comparing clinical outcomes and costs for participants receiving each of the trial treatments. Resource utilisation data and unit cost data (e.g. basic costs, literature and other health-economic data) will be collected as part of the trial dataset and also collated based on datasets collected in other trials of acutely sick children.

b. Molecular diagnostics

Two molecular methods are being used increasingly in research and clinical practice to identify bacteria. 16S ribosomal deoxyribonucleic acid (16S rDNA), common to all species of bacteria, can be detected with a broad-range polymerase chain reaction (PCR); specific quantitative PCR (qPCR) can also be used to quantify the 16S rDNA subunit to measure directly the number of bacteria. However broad range 16S rDNA PCR is subject to artefact from endogenous and exogenous bacterial products [63-65] and therefore without either sequencing the PCR product, or carrying out more sensitive qPCR, there is concern that changes in the qPCR may not be due to circulating organisms. Unfortunately sequencing the 16S rDNA has so far yielded results compatible with environmental contamination rather than recognised gut commensals. The use of specific primers renders qPCR less vulnerable to background contaminants than broad-range 16S rDNA PCR, and therefore more sensitive [66, 67]. The disadvantage of qPCR is the need to predict which bacterial species are likely to be relevant.

The goal of this study would be to identify the role of bacteria in the aetiology of lethal pneumonia in African children. Previous studies have shown comparable results between frozen EDTA plasma and whole blood, and so we would assay standard 16S rDNA PCR, and a panel of 10 qPCR reactions (including Enterobacteriaceae, a panel of anaerobes, Streptococcus pneumoniae, Staphylococcus aureus, group A streptococcus).

White and red cell pellets collected into a 2ml EDTA bottle taken at enrolment, (stored at -80°C) and plasma will be and shipped at the conclusion of the study to Kilifi, Kenya and molecular diagnostics will compare the range of pathogens identified in cases (deaths) and controls (survivors) frequency matched by age group, study site and season.

c. Investigation of respiratory viruses

Respiratory viruses (RVs) are a major cause of acute lower respiratory tract infections worldwide [68]. However, in developing countries the potential role of RVs in life-threatening disease and mortality remains uncertain, e.g. influenza [69]. The present trial, in which samples will be collected from children with a high mortality risk, provides an important opportunity to elucidate this role. Furthermore, data are scarce on the occurrence of RVs in paediatric pneumonia cases in Uganda or Congo, and almost nothing is known for Central and Eastern Africa on the origins and spread of these viruses. Nasal pharyngeal/oro-pharyngeal swabs samples will be stored and batched transferred to Professor Nokes Viral pathogen laboratory in Kilifi, Kenya. Ribonucleic acid (RNA) extracted from NP/OP swabs will be screened for a broad range of RVs, including influenza viruses, Respiratory Syncytial Virus (RSV), coronaviruses and rhinoviruses using a multiplex real time PCR assay system [70]. Partial and/or whole genome sequencing (WGS) of the virus positive samples, for example RSV [71], and influenza [72], amongst other respiratory targets will be undertaken.

The outcomes of this study will be:

(i) the prevalence and seasonal patterns of a range of respiratory viruses in children with life threatening features of respiratory disease;
(ii) estimates of the risk of death in RV positive relative to negative children; and
(iii) information on the sources of viruses and patterns of spread in East and Central Africa.

d. Electrical Impedance Tomography (2h)

In a sub-study at one site, we will study response to both high-flow and low-flow using a non-invasive measure of assessing quality of ventilation by non-invasive electrical impedance tomography, which monitors end-expiratory lung volumes, functional residual capacity and breath volumes. It is simple to set up as it requires, an array of 16 electrodes (integrated into a silicone belt: Respiband) which is placed around the patient's chest. The SensorMedics equipment (Goetting Tomograph) enables sequential measurements to make it possible to ascertain the ventilation distribution in the lungs both chronologically and spatially, enabling immediate conclusions about the pathophysiological processes and arrange the individual tomograms sequentially results in color-coded images (see below). We aim to recruit 20 children in each of the randomisation arms for both strata eg 100 overall. EIT data will be collected at time points corresponding to the clinical assessments at 1 hour (since baseline will not be possible), 2-,4, 8, 12-, 24 and 48-hours.

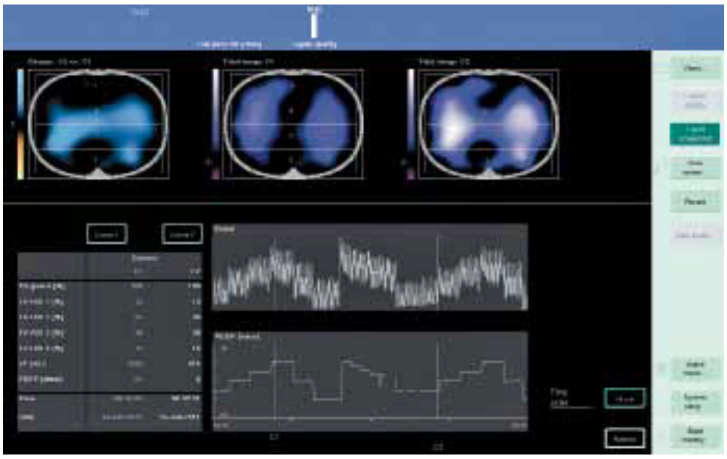

Trend analysis

We will (retrospectively) compare ventilation images overtime in children by randomised arms. White represents the best-ventilated regions of the lungs, black: non-ventilated regions blue: areas represent a transitory phase between black and white or vice-versa. These may provide important mechanistic data supporting the final trial results.

e. Short PeRiod INcidence sTudy of Severe Acute Respiratory Infection (SPRINT-SARI)

SPRINT-SARI is a global multi-network, multicentre, prospective, short period incidence observational study of all severe SARI patients admitted to the in-patient unit of interest (ICU or hospital ward), during a defined 5-or 7-day study period once annually. The Chief investigator is Prof Steven Webb, Clinical Professor, University of Western Australia, Royal Perth Hospital. The primary aim of this study is to establish a research response capability for future epidemics / pandemics through a global SARI observational study. The secondary aim of this study is to describe the clinical epidemiology and microbiology profiles of patients with SARI. Since children with severe pneumonia will be enrolled across 5 sites (2 countries) in COAST this will act as an opportunity to provide data to this global platform. Only basic clinical and some diagnostic data will be provided from each site together will outcome data. There will be no linking of the data to the trial intervention and only fully anonymised and de-identified data will be submitted to the centrally coordinated database for SPRINT-SARI.

f. Sickle cell disease status

Children with sickle cell disease (SCD) admitted to hospital with triage features fulfilling severe and very severe pneumonia the mortality rate is three-fold high compared to children without SCD (Williams, unpublished). The reasons for high mortality in this group remains unclear and whether this may indicate a diagnosis of 'chest syndrome' or a much higher risk of bacterial infection including gram negative organisms. As sickle cell status is likely to influence the outcome in this study, we aim to test all children for this common condition in Africa (with SCD and sickle cell trait being present in 2% and 7% of children with SP and VSP).

White and red cell pellets collected into a 2ml EDTA bottle (see above *Molecular diagnostics*) will be stored at -80°C and shipped at the conclusion of the study to Professor Wiliiams haemoglobinophathy laboratory (Kilifi, Kenya). We will extract a sample of DNA from this sample from study participants to describe the distribution of HbSS using PCR.

Patients found to be positive for HbSS will be recalled for counselling and for confirmatory testing and if confirmed to be suffering from HbSS they will be encouraged to attend the outpatient clinic for regular treatment.

14 Ethical compliance

14.1 Trial registration

This trial has been registered with the ISRCTN Registry (**ISRCTN15622505**).

14.2 Central ethical compliance

Favourable ethics opinion has been obtained from the Imperial College REC. This trial will be submitted for approval by the relevant RECs/Institutional Review Boards and by required regulatory authorities in all participating countries.

The trial will be conducted in accordance with the recommendations for research on human subjects in the Declaration of Helsinki [73], the ICH-GCP guidelines (E6(R1), 1996) and the applicable national regulations.

14.3 Local ethical compliance

It is the responsibility of the PI to obtain the necessary local approvals and provide them to the KCTF prior to site activation.

COAST will only be conducted at sites where all necessary local approvals for the trial have been obtained and a Site Agreement between the Hospital (site) and the Imperial College London (Sponsor) has been signed.

14.4 Confidentiality and data protection

Participants' identification data will be collected as part of the trial follow-up procedures. The KCTF and the ICNARC CTU will preserve the confidentiality of participants taking part, which will comply with requirements for data protection in the countries where the research is being conducted. All data will be anonymised prior to presentation or publication of any results.

14.5 Patient, Carer and Public Involvement and Engagement

Each site would either use their existing Community Advisory Board (CAB) or form a specific patient liaison group to feedback concerns and questions from the community and hear about the latest developments in the trial and the wider scientific community, where possible.

Each of the country PIs have discussed the trial with their local hospital CABs (if in existence) and informed Ministry of Health about the trial. Dialogue with these groups will be maintained through regular briefing meetings during the course of the trial and will be a standing item at each TSC meeting (see Section 12.4), in order to rapidly facilitate dissemination and implementation of results. Policy briefs and presentations at national meetings will be used to disseminate results to policy makers.

14.6 Declaration of interests

All trial investigators have confirmed that they do not have any financial or other conflicts of interest to declare in relation to this trial.

15 Sponsorship and Funding

15.1 Sponsorship

Imperial College London will act as Sponsor for this trial and delegate the responsibility of overseeing the implementation of the trial to KCTF and the ICNARC CTU. Imperial College London holds negligent harm and non-negligent harm insurance policies, which apply to this trial.

15.2 Funding

The trial is supported by grant funding from the UK MRC, the UK Department for International Development (DFID) and the Wellcome Trust through the Joint Global Health Trials scheme. The trial is also supported by Fisher & Paykel Healthcare, who is providing equipment and technical support.

A written agreement with the site PI and/or the PI's institution and Imperial College London will outline the funding arrangements to sites. The TSC will meet and review the financial aspects of the trial at least annually and report to the Sponsor.

16 Dissemination policy

All publications and presentations relating to the trial will be authorised by the TMG. The first publication of the trial results will be in the name of the TMG, if this does not conflict with the journal's policy. If there are named authors, these will include the ChI, Trial Statistician and Trial Manager. Members of the TMG, TSC and DMC and other contributors will be cited by name, if this does not conflict with the journal's policy. Authorship of sub studies initiated outside of the TMG will be according to the individuals involved in the project but must acknowledge the contribution of the TMG and KCTF.

The TSC is the custodian of the data and specimens generated from the trial; trial data are not the property of individual participating investigators or health care facilities where the data were generated.

During the course and following completion of the trial there will be publications, including manuscripts and abstracts for presentations at national and international meetings, as well as the preparation of manuscripts for peer-reviewed publication. In order to avoid disputes regarding authorship, a consensus approach will be established that will provide a framework for all publications derived in full or in part from this trial. Authorship criteria will be determined using the guidelines provided by The International Committee of Medical Journal

Editors. The following approach is derived from the Lancet and from the publication policies used in other MRC clinical trials:

- All publications are to be approved by the TMG and TSC before submission for publication. Any publication arising before the end of the trial will also be approved by the DMC in order to ensure that the primary objective of the trial is not compromised. In particular, no analyses by randomised group of any outcome (primary, secondary or other) in either the main trial or associated sub studies will be conducted or presented before the end of the trial, other than those for interim review by the DMC. The TMG and TSC will resolve problems of authorship and maintain the quality of publications.

- Manuscripts arising from the trial will, wherever possible, be submitted to peer-reviewed journals which enable Open Access via UK PubMed Central within six months of the official date of final publication. All conference presentations will be made available as soon as possible after the event via the COAST website. All publications will acknowledge the trial's funding sources.

- For all publications, the TMG will nominate a chairperson or approve an individual's request to chair a manuscript writing committee. The chair will usually be the primary or senior author. The chairperson is responsible for identifying fellow authors and for determining the order of authorship that will appear on the manuscript. The TSC will resolve any problems of authorship and maintain the quality of publications.

- The TMG will maintain a list of investigators to be presented in the acknowledgements at the end of the paper. This list will include investigators who contributed to the investigation being reported but who are not members of the writing committee. In principle, sub study reports should include all investigators for the main trial, although in some instances, where a smaller number of investigators have made any form of contribution, it may be appropriate to abbreviate the listing.

- All headline authors in any publication arising from the main trial or sub studies must have a made a significant academic or project management contribution to the work that is being presented. “Significant” must be defined by a written declaration of exactly what the contribution of any individual is believed to have been. In addition to fulfilling the criteria based on contribution, additional features that will be considered in selecting an authorship group will include the recruitment of participants who contributed data to any set of analyses contained in the manuscript, the conduct of analyses (laboratory and statistical) and leadership and coordination of the project in the absence of a clear academic contribution.

- The data derived from this trial are considered the property of the TSC. The presentation or publication of any data collected by the participating investigators on participants recruited into this trial is under the direct control of the TMG and TSC (and the DMC before the end of the trial). This is true whether the publication or presentation is concerned directly with the results of the trial or is associated with the trial in some other way. However, although individual participating investigators will not have any inherent right to perform analyses or interpretations, to make public presentations or seek publication of any of the data other than under the auspices of and with the approval of the TMG and TSC (and the DMC before the end of the trial), they will be encouraged to

develop sub studies or propose analyses subject to the approval by the TMG and TSC (and the DMC before the end of the trial). Any requests for access to raw data will be welcomed as long as they are scientifically valid and do not conflict with the integrity of the trial or ongoing analyses by the trial team.

- Outcome data by randomised group will not be revealed to the participating investigators until the data collection phase and primary full analysis of the trial has been completed. This policy safeguards against possible bias affecting the data collection. The DMC will monitor the outcome results and may recommend that the trial be stopped for safety reasons or if a definitive answer is reached earlier than the scheduled end of the trial.

References

1. **Hospital Care for Children: guidelines for the management of common illnesses with limited resources.** In. Geneva, Switzerland: World Health Organization; 2005.
2. Black RE, Cousens S, Johnson HL, Lawn JE, Rudan I, Bassani DG, Jha P, Campbell H, Walker CF, Cibulskis R, Eisele T, Liu L, Mathers C: **Global, regional, and national causes of child mortality in 2008: a systematic analysis**. *Lancet* 2010, **375**:1969-1987.
3. Rudan I: **Epidemiology and etiology of childhood pneumonia**. *Bulletin of the World Health Organization* 2008, **86**:408-416.
4. Rajaratnam JK, Marcus JR, Flaxman AD, Wang H, Levin-Rector A, Dwyer L, Costa M, Lopez AD, Murray CJ: **Neonatal, postneonatal, childhood, and under-5 mortality for 187 countries, 1970-2010: a systematic analysis of progress towards Millennium Development Goal 4**. *Lancet* 2010, **375**:1988-2008.
5. **Hospital Care for Children. Guidelines for the management of common childhood illnesses with limited resources.** . In. Geneva: World Health Organization; 2005.
6. Mwaniki MK, Nokes DJ, Ignas J, Munywoki P, Ngama M, Newton CR, Maitland K, Berkley JA: **Emergency triage assessment for hypoxaemia in neonates and young children in a Kenyan hospital: an observational study**. *Bulletin of the World Health Organization* 2009, **87**:263-270.
7. Banajeh SM: **Outcome for children under 5 years hospitalized with severe acute lower respiratory tract infections in Yemen: a 5 year experience**. *Journal of tropical pediatrics* 1998, **44**:343-346.
8. Subhi R, Adamson M, Campbell H, Weber M, Smith K, Duke T: **The prevalence of hypoxaemia among ill children in developing countries: a systematic review**. *The Lancet infectious diseases* 2009, **9**:219-227.
9. Davis PG, Tan A, O'Donnell CP, Schulze A: **Resuscitation of newborn infants with 100% oxygen or air: a systematic review and meta-analysis**. *Lancet* 2004, **364**:1329-1333.
10. **Recommendations for management of common childhood conditions: evidence for technical update of pocket book recommendations**. In. Geneva: World Health Organization; 2012.
11. Rojas-Reyes M, Granados Rugeles C, Charry-Anzola L: **Oxygen therapy for lower respiratory tract infections in children between 3 months and 15 years of age**. *Cochrane Database Syst Rev* 2009, .
12. Hammitt LL, Kazungu S, Morpeth SC, Gibson DG, Mvera B, Brent AJ, Mwarumba S, Onyango CO, Bett A, Akech DO, Murdoch DR, Nokes DJ, Scott JA: **A preliminary study of pneumonia etiology among hospitalized children in Kenya**. *Clin Infect Dis* 2012, **54 Suppl 2**:S190-199.
13. Graham SM, English M, Hazir T, Enarson P, Duke T: **Challenges to improving case management of childhood pneumonia at health facilities in resource-limited settings**. *Bulletin of the World Health Organization* 2008, **86**:349-355.
14. Duke T, Wandi F, Jonathan M, Matai S, Kaupa M, Saavu M, Subhi R, Peel D: **Improved oxygen systems for childhood pneumonia: a multihospital effectiveness study in Papua New Guinea**. *Lancet* 2008, **372**:1328-1333.
15. Magree HC, Russell FM, Sa'aga R, Greenwood P, Tikoduadua L, Pryor J, Waqatakirewa L, Carapetis JR, Mulholland EK: **Chest X-ray-confirmed pneumonia in children in Fiji**. *Bulletin of the World Health Organization* 2005, **83**:427-433.
16. Djelantik IG, Gessner BD, Sutanto A, Steinhoff M, Linehan M, Moulton LH, Arjoso S: **Case fatality proportions and predictive factors for mortality among children hospitalized with severe pneumonia in a rural developing country setting**. *Journal of tropical pediatrics* 2003, **49**:327-332.

17. **Pocket Book of Hospital Care for Children: Guidelines for the management of common childhood illnesses**. In., Second edn. Geneva: World Health Organization; 2013.
18. Belle J, Cohen H, Shindo N, Lim M, Velazquez-Berumen A, Ndihokubwayo JB, Cherian M: **Influenza preparedness in low-resource settings: a look at oxygen delivery in 12 African countries**. *Journal of infection in developing countries* 2010, **4**:419-424.
19. Hill SE, Njie O, Sanneh M, Jallow M, Peel D, Njie M, Weber M, Hill PC, Adegbola RA, Howie SR: **Oxygen for treatment of severe pneumonia in The Gambia, West Africa: a situational analysis**. *Int J Tuberc Lung Dis* 2009, **13**:587-593.
20. Matai S, Peel D, Wandi F, Jonathan M, Subhi R, Duke T: **Implementing an oxygen programme in hospitals in Papua New Guinea**. *Annals of tropical paediatrics* 2008, **28**:71-78.
21. La Vincente SF, Peel D, Carai S, Weber MW, Enarson P, Maganga E, Soyolgerel G, Duke T: **The functioning of oxygen concentrators in resource-limited settings: a situation assessment in two countries**. *Int J Tuberc Lung Dis* 2011, **15**:693-699.
22. Musumba CO, Pamba AO, Sasi PA, English M, Maitland K: **Salicylate poisoning in children: report of three cases**. *East African medical journal* 2004, **81**:159-163.
23. Catto AG, Zgaga L, Theodoratou E, Huda T, Nair H, El Arifeen S, Rudan I, Duke T, Campbell H: **An evaluation of oxygen systems for treatment of childhood pneumonia**. *BMC Public Health* 2011, **11 Suppl 3**:S28.
24. Rojas-Reyes MX, Granados Rugeles C, Charry-Anzola LP: **Oxygen therapy for lower respiratory tract infections in children between 3 months and 15 years of age**. *Cochrane Database Syst Rev* 2014, **12**:CD005975.
25. Smyth A, Carty H, Hart CA: **Clinical predictors of hypoxaemia in children with pneumonia**. *Annals of tropical paediatrics* 1998, **18**:31-40.
26. Shann F, Dobson M, Peel D: **Oxygen therapy for acute respiratory infections in young children in developing countries**. In. Geneva: World Health Organization; 1993.
27. Jackson RM: **Pulmonary oxygen toxicity**. *Chest* 1985, **88**:900-905.
28. Hayes RA, Shekar K, Fraser JF: **Hyperoxic damage and the need for optimised oxygenation practices**. *Crit Care* 2013, **17**:441.
29. Munkeby BH, Borke WB, Bjornland K, Sikkeland LI, Borge GI, Halvorsen B, Saugstad OD: **Resuscitation with 100% O2 increases cerebral injury in hypoxemic piglets**. *Pediatric research* 2004, **56**:783-790.
30. Tan A, Schulze A, O'Donnell CP, Davis PG: **Air versus oxygen for resuscitation of infants at birth**. *Cochrane Database Syst Rev* 2005:CD002273.
31. Cabello JB, Burls A, Emparanza JI, Bayliss S, Quinn T: **Oxygen therapy for acute myocardial infarction**. *Cochrane Database Syst Rev* 2010:CD007160.
32. Ronning OM, Guldvog B: **Should stroke victims routinely receive supplemental oxygen? A quasi-randomized controlled trial**. *Stroke; a journal of cerebral circulation* 1999, **30**:2033-2037.
33. Perrin K, Wijesinghe M, Healy B, Wadsworth K, Bowditch R, Bibby S, Baker T, Weatherall M, Beasley R: **Randomised controlled trial of high concentration versus titrated oxygen therapy in severe exacerbations of asthma**. *Thorax* 2011, **66**:937-941.
34. Mikkelsen ME, Christie JD, Lanken PN, Biester RC, Thompson BT, Bellamy SL, Localio AR, Demissie E, Hopkins RO, Angus DC: **The adult respiratory distress syndrome cognitive outcomes study: long-term neuropsychological function in survivors of acute lung injury**. *American journal of respiratory and critical care medicine* 2012, **185**:1307-1315.
35. Bass JL, Corwin M, Gozal D, Moore C, Nishida H, Parker S, Schonwald A, Wilker RE, Stehle S, Kinane TB: **The effect of chronic or intermittent hypoxia on cognition in childhood: a review of the evidence**. *Pediatrics* 2004, **114**:805-816.

36. Cunningham S, Rodriguez A, Adams T, Boyd KA, Butcher I, Enderby B, MacLean M, McCormick J, Paton JY, Wee F, Thomas H, Riding K, Turner SW, Williams C, McIntosh E, Lewis SC, Bronchiolitis of Infancy Discharge Study g: **Oxygen saturation targets in infants with bronchiolitis (BIDS): a double-blind, randomised, equivalence trial**. *Lancet* 2015, **386**:1041-1048.
37. Maitland K, Kiguli S, Opoka RO, Engoru C, Olupot-Olupot P, Akech SO, Nyeko R, Mtove G, Reyburn H, Lang T, Brent B, Evans JA, Tibenderana JK, Crawley J, Russell EC, Levin M, Babiker AG, Gibb DM: **Mortality after fluid bolus in African children with severe infection**. *The New England journal of medicine* 2011, **364**:2483-2495.
38. Frey B, McQuillan PJ, Shann F, Freezer N: **Nasopharyngeal oxygen therapy produces positive end-expiratory pressure in infants**. *European journal of pediatrics* 2001, **160**:556-560.
39. Frey B, Shann F: **Oxygen administration in infants**. *Archives of disease in childhood Fetal and neonatal edition* 2003, **88**:F84-88.
40. Benaron DA, Benitz WE: **Maximizing the stability of oxygen delivered via nasal cannula**. *Archives of pediatrics & adolescent medicine* 1994, **148**:294-300.
41. Dysart K, Miller TL, Wolfson MR, Shaffer TH: **Research in high flow therapy: mechanisms of action**. *Respiratory medicine* 2009, **103**:1400-1405.
42. Mayfield S, Jauncey-Cooke J, Hough JL, Schibler A, Gibbons K, Bogossian F: **High-flow nasal cannula therapy for respiratory support in children**. *The Cochrane database of systematic reviews* 2014, **3**:CD009850.
43. Spence KL, Murphy D, Kilian C, McGonigle R, Kilani RA: **High-flow nasal cannula as a device to provide continuous positive airway pressure in infants**. *Journal of perinatology : official journal of the California Perinatal Association* 2007, **27**:772-775.
44. Lampland AL, Plumm B, Meyers PA, Worwa CT, Mammel MC: **Observational study of humidified high-flow nasal cannula compared with nasal continuous positive airway pressure**. *The Journal of pediatrics* 2009, **154**:177-182.
45. Kubicka ZJ, Limauro J, Darnall RA: **Heated, humidified high-flow nasal cannula therapy: yet another way to deliver continuous positive airway pressure?** *Pediatrics* 2008, **121**:82-88.
46. McGinley B, Halbower A, Schwartz AR, Smith PL, Patil SP, Schneider H: **Effect of a high-flow open nasal cannula system on obstructive sleep apnea in children**. *Pediatrics* 2009, **124**:179-188.
47. Roca O, Riera J, Torres F, Masclans JR: **High-flow oxygen therapy in acute respiratory failure**. *Respiratory care* 2010, **55**:408-413.
48. Lee JH, Rehder KJ, Williford L, Cheifetz IM, Turner DA: **Use of high flow nasal cannula in critically ill infants, children, and adults: a critical review of the literature**. *Intensive care medicine* 2013, **39**:247-257.
49. Schibler A, Pham TM, Dunster KR, Foster K, Barlow A, Gibbons K, Hough JL: **Reduced intubation rates for infants after introduction of high-flow nasal prong oxygen delivery**. *Intensive care medicine* 2011, **37**:847-852.
50. Corley A, Caruana LR, Barnett AG, Tronstad O, Fraser JF: **Oxygen delivery through high-flow nasal cannulae increase end-expiratory lung volume and reduce respiratory rate in post-cardiac surgical patients**. *British journal of anaesthesia* 2011, **107**:998-1004.
51. Chisti MJ, Salam MA, Smith JH, Ahmed T, Pietroni MA, Shahunja KM, Shahid AS, Faruque AS, Ashraf H, Bardhan PK, Sharifuzzaman, Graham SM, Duke T: **Bubble continuous positive airway pressure for children with severe pneumonia and hypoxaemia in Bangladesh: an open, randomised controlled trial**. *Lancet* 2015, **386**:1057-1065.
52. Shann F, Lange T: **Bubble CPAP for pneumonia: perils of stopping trials early**. *Lancet* 2015, **386**:1020-1022.
53. Howitt P, Darzi A, Yang GZ, Ashrafian H, Atun R, Barlow J, Blakemore A, Bull AM, Car J, Conteh L, Cooke GS, Ford N, Gregson SA, Kerr K, King D, Kulendran M,

Malkin RA, Majeed A, Matlin S, Merrifield R, Penfold HA, Reid SD, Smith PC, Stevens MM, Templeton MR, Vincent C, Wilson E: **Technologies for global health**. *Lancet* 2012, **380**:507-535.
54. Maitland K, Molyneux S, Boga M, Kiguli S, Lang T: **Use of deferred consent for severely ill children in a multi-centre phase III trial**. *Trials* 2011, **12**:90.
55. **Hospital Care for Children. Guidelines for the management of common childhood illnesses. Second Edition**. In. Geneva: World Health Organization; 2013.
56. Liu LL, Gallaher MM, Davis RL, Rutter CM, Lewis TC, Marcuse EK: **Use of a respiratory clinical score among different providers**. *Pediatric pulmonology* 2004, **37**:243-248.
57. van Dijk M, de Boer JB, Koot HM, Tibboel D, Passchier J, Duivenvoorden HJ: **The reliability and validity of the COMFORT scale as a postoperative pain instrument in 0 to 3-year-old infants**. *Pain* 2000, **84**:367-377.
58. Abubakar A, Holding P, Van de Vijver F, Bomu G, Van Baar A: **Developmental monitoring using caregiver reports in a resource-limited setting: the case of Kilifi, Kenya**. *Acta Paediatr* 2010, **99**:291-297.
59. Abubakar A, Holding P, van Baar A, Newton CR, van de Vijver FJ: **Monitoring psychomotor development in a resource-limited setting: an evaluation of the Kilifi Developmental Inventory**. *Annals of tropical paediatrics* 2008, **28**:217-226.
60. Union EPaCoE: **Directive 2001/20/EC of European Parliament and of the Council of 4 April 2001 on the approximation of the laws, regulations and administrative provisions of the Member States relating to the implementation of good clinical practice in the conduct of clinical trials on medicinal products for human use**. *Official Journal L121* 2001.
61. Ranchord AM, Perrin K, Weatherall M, Beasley R, Simmonds M: **A randomised controlled trial of the effect of high concentration oxygen on myocardial ischaemia during exercise**. *International journal of cardiology* 2012, **160**:201-205.
62. Landau S, Stahl D: **Sample size and power calculations for medical studies by simulation when closed form expressions are not available**. *Stat Methods Med Res* 2013, **in press**.
63. Harris KA, Hartley JC: **Development of broad-range 16S rDNA PCR for use in the routine diagnostic clinical microbiology service**. *J Med Microbiol* 2003, **52**:685-691.
64. Millar BC, Xu J, Moore JE: **Risk assessment models and contamination management: implications for broad-range ribosomal DNA PCR as a diagnostic tool in medical bacteriology**. *Journal of clinical microbiology* 2002, **40**:1575-1580.
65. Ferri E, Novati S, Casiraghi M, Sambri V, Genco F, Gulminetti R, Bandi C: **Plasma levels of bacterial DNA in HIV infection: the limits of quantitative polymerase chain reaction**. *The Journal of infectious diseases* 2010, **202**:176-177; author reply 178.
66. Marchetti G, Bellistri GM, Borghi E, Tincati C, Ferramosca S, La Francesca M, Morace G, Gori A, Monforte AD: **Microbial translocation is associated with sustained failure in CD4+ T-cell reconstitution in HIV-infected patients on long-term highly active antiretroviral therapy**. *AIDS* 2008, **22**:2035-2038.
67. Merlini E, Bai F, Bellistri GM, Tincati C, d'Arminio Monforte A, Marchetti G: **Evidence for Polymicrobic Flora Translocating in Peripheral Blood of HIV-Infected Patients with Poor Immune Response to Antiretroviral Therapy**. *PloS one* 2011, **6**:e18580.
68. Shi T, McLean K, Campbell H, Nair H: **Aetiological role of common respiratory viruses in acute lower respiratory infections in children under five years: A systematic review and meta-analysis**. *Journal of global health* 2015, **5**:010408.
69. Nair H, Brooks WA, Katz M, Roca A, Berkley JA, Madhi SA, Simmerman JM, Gordon A, Sato M, Howie S, Krishnan A, Ope M, Lindblade KA, Carosone-Link P, Lucero M, Ochieng W, Kamimoto L, Dueger E, Bhat N, Vong S, Theodoratou E, Chittaganpitch

M, Chimah O, Balmaseda A, Buchy P, Harris E, Evans V, Katayose M, Gaur B, O'Callaghan-Gordo C *et al*: **Global burden of respiratory infections due to seasonal influenza in young children: a systematic review and meta-analysis**. *Lancet* 2011, **378**:1917-1930.
70. Hammitt LL, Kazungu S, Welch S, Bett A, Onyango CO, Gunson RN, Scott JA, Nokes DJ: **Added value of an oropharyngeal swab in detection of viruses in children hospitalized with lower respiratory tract infection**. *Journal of clinical microbiology* 2011, **49**:2318-2320.
71. Agoti CN, Otieno JR, Munywoki PK, Mwihuri AG, Cane PA, Nokes DJ, Kellam P, Cotten M: **Local evolutionary patterns of human respiratory syncytial virus derived from whole-genome sequencing**. *Journal of virology* 2015, **89**:3444-3454.
72. Baillie GJ, Galiano M, Agapow PM, Myers R, Chiam R, Gall A, Palser AL, Watson SJ, Hedge J, Underwood A, Platt S, McLean E, Pebody RG, Rambaut A, Green J, Daniels R, Pybus OG, Kellam P, Zambon M: **Evolutionary dynamics of local pandemic H1N1/2009 influenza virus lineages revealed by whole-genome analysis**. *Journal of virology* 2012, **86**:11-18.
73. **Declaration of Helsinki - Ethical Principles for Medical Research Involving Human Subjects**. In. Edited by Association WM. Helsinki; 1964.

Appendix

Appendix A Protocol version history

**Protocol:**			
**Version no.**	**Date**	**Amendment no.**	**Protocol Section (no./title)**
v1.0	25th January 2016	N/A	N/A
V2.0	7th July 2016	1st	
V 2.1	11th January 2017	2nd	

Appendix B Expected adverse events

Expected AEs that could be observed in participants up to 28 days following randomisation:

- Nasal trauma
- Facial trauma
- Pneumothorax
- Subcutaneous emphysema
- Aspiration

[This list is not exhaustive. If an AE, as defined in section 10.1, occurs, then it should be recorded and reported as described in section 10.6]

Appendix C Parent/Guardian Information Sheet

A: Introduction

We are inviting your child to take part in a research study called COAST. It is being conducted at five hospitals in Africa and will include 4,200 infants and children aged between 28 days to 12 years. Before you decide if you want your child to take part, it is important for you to understand why the research is being done and what it will involve. Please take time to read this information sheet carefully or ask someone to read it to you. Please discuss this with the doctors or nurses and ask questions if there is anything that is not clear or if you would like more information. Joining the COAST study is entirely voluntary. Take time to decide whether or not you wish your child to take part.

B: Study purpose: **What is the reason for doing the COAST study?**

Your child has been admitted to hospital because they have symptoms of a chest infection which is causing them to breath faster and with extra effort. The small instrument with a band/clip around your child's finger (pulse oximeter: [*show*]) found that the oxygen level in your child's blood is lower than normal– this indicates that the lungs/chest may not be working properly. This would normally be treated, if available, by giving your child oxygen. However, we don't know whether the oxygen treatment we give your child helps them get better and we also don't know how best to give this treatment – either the flow rate should be fast or slow. So, the COAST study is trying to find out the best treatment to help your child breathe more easily and improve the way their lungs are working – this may or may not involve giving your child oxygen.

C: Study Procedures: **What will it involve for my child?**
What treatments will he/she be given?

Your child will be given all the usual treatment for their illness, according to standard <COUNTRY> health guidelines. If you agree for your child to take part in this study, they will receive treatment based on how low the oxygen level is in their blood:

If their oxygen level is very low, your child will receive oxygen that will be given by one of two different flow rates:

- At a lower flow rate from a tube connected to an oxygen cylinder that will deliver oxygen by mask or through some soft prongs into your child's nose
- At a higher flow rate which delivers oxygen/air (which is warmed and humidified) from a cylinder at a higher flow to soft prongs into your child's nose

If your child oxygen level is moderately low, your child will receive oxygen given in two different ways (*as explained above or can be repeated*) or not receive oxygen immediately.

- At a lower flow rate from a tube connected to an oxygen cylinder that will deliver oxygen by mask or through some soft prongs into your child's nose
- At a higher flow rate which delivers oxygen/air (which is warmed and humidified) from a cylinder at a higher flow to soft prongs into your child's nose
- If your children receives no immediate oxygen we will monitor your child's oxygen levels very closely to see if they get better without the need for oxygen. However, if the oxygen level drops to a very low level we will provide oxygen treatment given at a lower flow rate.

Once they start treatment, the doctors/nurses will study the effect on the child's breathing and oxygen levels. The doctors and nurses will take extra and regular measurements at the bedside to help us find out if the child is getting better or not. All the instruments and devices used in this study are very safe, and have been used in lots of other children in many areas of the world. Even though they are unlikely to cause your child any harm or discomfort we will monitor them closely for any adverse effects.

Study Procedures

1. We will do some initial blood tests to check how sick your child is and some additional ones to see if they are recovering or not. We should be able to discover these during these regular checks and treat them promptly. At the time of hospital admission this will involve 5-8mls (one to two teaspoons depending on the size of your child) plus 2mls extra which will be saved for future tests to help us find out why your child became ill. You will get some of the results back during the study – others will be done much later at the end of the study and you may not get the results of some of these tests. After the initial blood tests, we will only use blood tests which are taken from the child's fingertip, which will help us adjust other treatments that your child may need (i.e. a transfusion or some extra sugar).
2. As explained above your child will be carefully monitored and checked during their time in hospital. If they respond well to the treatment, we may stop the oxygen/air and observe over a time period to see if they fully recover – they will continue with their other usual treatments. If your child if they do not respond well, for example do not like the high flow oxygen then we will switch the flow rate to low flow oxygen. If your child is not receiving oxygen and their oxygen levels drop then we will then start oxygen by low flow as is usual standard of care.

3. When your child is ready to go home, we will ask you to come back in one month for a health check. We will check where you live and take your contact details, to help us find you if you are not able to come back. You will have our details so that you can contact us if you have any concerns about your child's condition or if they go back to hospital – and our team will call you back if necessary. If they have any illnesses, we will treat these or refer them to another specialist if necessary. Some of the blood taken at this visit will be stored, approximately 2-3ml (equivalent to half a teaspoon) to check your child's haemoglobin level and do a malaria test and to store some blood for research into why your child became sick.
4. Some of the tests to find out what caused your child's illness are needed as part of this research cannot be done in this country at the moment, so part of the samples will be sent to laboratories overseas. This will involve a small portion of the blood that was taken during the study, which we will store. Some of the tests we will do will look at whether your child has a trait or characteristic that they inherited from their parents that will make them more vulnerable to severe illness. This is called genetic research. Individual names will be removed and will be replaced by codes, so that information cannot be linked to participants. Future research done on these samples will be approved by a national independent expert committee, to ensure that participants' safety and rights are respected.

<u>D: Risks of study participation</u>

There are very few risks to your child being in this study. If for any reason the doctor thinks that it is not in your child's best interest to be in the study then they will not be enrolled in the study but will be given their usual treatment. You do not have to pay anything to join the study.

If your child does not respond to the treatment, then we may increase the rate of flow or amount of oxygen they receive until we see signs on the monitors that they are getting better. If they do not respond (i.e. they are still working very hard to breathe), the doctors/nurses will discuss with you whether they may need longer for the treatment to work and/or whether you would prefer that your child switches to the usual standard of care, which may involve oxygen delivery at a lower rate.

All children will have blood taken as part of this study. Many of the tests that will be done in hospital would also be done if you chose not to join the study. However, we will take a small amount of extra blood at the follow-up visit(s) – see above.

<u>E: Benefits of study participation</u>

Your child will get no direct benefits from this study. However, your child will get close observation during the study, and by taking part your child may help us improve the care of children who have breathing difficulties in the future. Regular assessment of your child by doctors/nurses will enable us to make important changes to your child's treatment in hospital, if these are needed. We will help supply routine medical supplies and treatments for your child to the hospital, so that you will not have to buy any treatments. This will mean that there will be no delay to starting treatment for your child. The medical tests we perform during this illness will also be paid for by the study.

You will be asked to bring your child back for follow up visit(s), and we will pay for your transport from hospital to your home and back to the clinic so you can attend this important visit. During the follow up visit(s), we will treat any illnesses we find, or arrange referral to appropriate clinic or hospital.

F: Alternatives to study participation: **What will happen if I don't agree to participate?**

All participation in research is voluntary. You are free to decide if you want your child to take part or not. Your child will still receive the recommended standard of care treatment if they do not take part. If you do agree to join the study you can change your mind at any time, and can withdraw your child from the research. This will not affect their care now or in the future and not incur any penalties. We hope that if you decide to withdraw later, you would give a reason for your decision.

G: Compensation

You will not incur any costs from participation in this study. All your travel expenses for attending the visits we invite you too will be paid, based on the cost of public transport to and from your home. As well, when you bring your child for follow up, snacks and drinks will be available and for meals, in a situation where you have to wait for a long time before being attended to.

H: Confidentiality: **Who will have access to information about me/my child in this research?**

All our research records are stored securely in locked cabinets and password protected computers. Only a few people who are working closely on the study will be able to view information from your child. When we report on the results of the study will not include any private information that will make it possible to identify your child.

I: Study related injury

This research is supported by Imperial College London who holds insurance policies, which apply to this study. If you experience harm or injury as a result of taking part in this study,

you will be eligible to claim compensation without having to prove that Imperial College is at fault. Some specific treatment and compensation are not included in our insurance policies and if you want more information about this you should discuss it with your doctor.

J: Contacts and questions

Who has allowed this research to take place?

All research conducted in <COUNTRY> is approved by national independent expert committees to make sure the research is conducted properly and that study participants' safety and rights are respected.

What if I have any questions?

You may ask any of our staff questions at any time. You can also contact those who are responsible for the care of your child and this research:

<NAMED SITE PI AND CONTACT DETAILS >

If you have any questions about your rights as a participant, please contact:

<NAMED SITE PI AND CONTACT DETAILS >

<COUNTRY Ethics Research Board>

Appendix D Consent Form

<Insert HOSPITAL HEADER>

Children's Oxygenation Administration Strategies Trial (COAST)

Date	DD	MM	YYYY	Patient IP. Number							

Child's Initials				Trial Number							

Please initial (or mark) box if you agree:

I confirm that I have read/been read the Patient Information Sheet (version 1.0) for the COAST trial and that I understand what will be required if my child participates in the trial. The trial has been explained to me and my questions have been answered.	
I understand that my child's participation is voluntary and that I am free to withdraw him or her at any time, without giving any reason, without my medical care or legal rights or my child's medical care or legal rights being affected.	
I understand that sections of any of my child's medical notes may be looked at by responsible individuals involved in the running of the trial or from regulatory authorities where it is relevant to my child's participation in this research. I give permission for these individuals to have access to my child's records, but understand that strict confidentiality will be maintained.	
I understand that my child will be treated by the COAST doctor/nurse, after discharge from hospital, will be followed up at day 28 (and day 90 if needed). After the trial, my child's healthcare will be provided by the national health system.	
I agree to allow blood samples to be taken from my child and for my child's samples to be stored for later testing. I understand that my child and I may not be given the results of tests performed on stored samples.	
I agree to samples being exported overseas for further studies.	
**I agree for my child to participate in the COAST trial.**	

Parent/carer's signature (or thumbprint)	Print name	Date (day/month/year) and Time

Witness's signature (if thumbprint used above)	Print name	Date (day/month/year) and Time

Doctor's signature	Print name	Date (day/month/year) and Time

IMPORTANT: One signed original to be kept in COAST trial file by the researcher, one signed copy to be given to the parent/guardian/carer and one signed copy to be kept in the clinic notes.

Appendix E Verbal Assent Form

<u>**C**hildren's **O**xygenation **A**dministration **S**trategies **T**rial (COAST)</u>

Verbal Assent Form

Child's Initials					Male ❍ Female ❍		Date/Year of Birth	D	D	M	M	M	Y	Y	Y	Y		Age (years)		

Date of Form	D	D	M	M	M	2	0	Y	Y		Clinic/Hospital Number								

NOTE: For children who are critically ill and in whom informed consent would lead to significant delay in starting treatment a verbal assent will be obtained by the doctor from the *parent or guardian* after brief discussion with admitting COAST doctor or nurse.

We advise that this should include the following phrases:

- We are going to provide the treatment for your child that is recommended by the government.
- We want to find out if we can improve on these current recommendations by trying new treatments that we think will work better and we do this by research.
- All research is checked by independent committees to make sure that the potential benefits to individuals outweigh the risks. All participation in research is voluntary, and so you can refuse.
- We would like your child to participate in this research for us to learn the best way to treat respiratory distress complicated by hypoxia.
- Do you agree for your child to take part in this research? You can say no and your child will still receive the same level of care with the government's recommended treatment.

Parent/Guardian assents to research?	**Please circle:** Yes / No

Parent or guardian's name	Relationship with child	Time (24 hour clock)			
		H	H	M	M

Doctor or nurse's signature	Print name	Date								
		D	D	M	M	M	2	0	Y	Y

IMPORTANT: One signed original copy to be kept in COAST trial file by the researcher, one signed copy to be given to parent/guardian/carer and one signed copy to be kept in the clinic notes.

Appendix F Withdrawal Form

COAST Withdrawal Form

Date	DD	MM	YYYY	Patient IP. Number							

Child's Initials				Trial Number							

Please initial (or mark) box if you agree:

I/my child no longer wish my child to participate in the COAST trial and do not wish to complete the further clinical assessment and follow-up. I/my child agree(s) to being contacted in the future (home visits or telephone) and to my/my child's medical records being consulted in future to obtain clinical information for COAST.	

Need to set up a procedure to follow the child up through visits and medical records and report any trial outcomes on the appropriate form. Inform the child and carer that s/he may still return for follow-up visits only or for further trial drugs and follow-up visits at a later date if they change their mind.

I/my child no longer wish my child to participate in the COAST trial and do not wish to complete the further clinical assessment and follow-up. I/we do not agree to being contacted in the future or to my/my child's medical records being consulted in future to obtain clinical information for the COAST trial.	

Discontinue all follow-up through medical records.

Parent/carer's signature (or thumbprint)	Print name	Date (day/month/year)

Child's signature (or thumbprint), where appropriate	Print name	Date (day/month/year)

Witness's signature (if thumbprint used above)	Print name	Date (day/month/year)

Doctor's signature	Print name	Date (day/month/year)

IMPORTANT: One signed original copy to be kept in COAST trial file by the researcher, one signed copy to be given to parent/guardian/carer and one signed copy to be kept in the clinic notes.

COAST Withdrawal Form v1.0 14 December 2015 1
