# Supplementary material for: Randomised controlled trial of oxygen therapy and high-flow nasal therapy in African children with pneumonia
Source: Intensive Care Med. 2021 May 5;47(5):566–76. doi: 10.1007/s00134-021-06385-3 (PMC8098782; doi:10.1007/s00134-021-06385-3)
Supplement: Supplementary file 3 — Supplementary file1 (PDF 484 KB) [file 134_2021_6385_MOESM3_ESM.pdf]

Children's Oxygen Administration Strategies Trial : COAST

Statistical Analysis Plan

Version 1.0, 17th March 2020

**Protocol version & date**	3.2 30th August 2018
**ICREC number:**	15IC3100
**Trial sponsor:**	Imperial College London
**Trial sponsor reference:**	P46493
**Trial funder(s):**	Joint Global Health Trials scheme:<br>Medical Research Council<br>Department for International Development<br>Wellcome Trust
**Funder reference:**	MR/L004364/1
**ISRCTN number:**	ISRCTN15622505
**Chief investigator:**	Prof Kathryn Maitland
**Author:**	Karen Thomas

Roles, responsibility, signatures

Role	Name	Signature	Date
Chief Investigator	Prof Kathryn Maitland	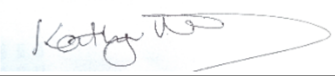	17th March 2020
Senior Statistician	Prof David Harrison	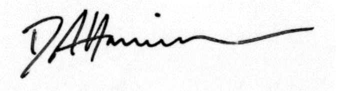	17th March 2020

Version history

Version number	Date	Summary of main changes from previous versions	Timing
1.0	17th March 2020	N/A	Pre-data lock

Contents

Abbreviations 5

Glossary of terms 5

1. Background 6

2. Objective and hypotheses 6

3. Study design 6

3.1 Inclusion/exclusion criteria 6

3.1.1 Inclusion criteria 6

3.1.2 Exclusion criteria 7

3.2 Study Treatments 7

3.2.1 COAST A 7

3.2.1.1 Intervention group 7

3.2.1.2 Control group 8

3.2.2 COAST B 8

3.2.2.1 Intervention groups 8

3.2.2.2 Control group 8

3.3 Randomisation 9

3.4 Outcome measures 9

3.4.1 Primary 9

3.4.2 Secondary 9

3.5 Framework 10

3.6 Timing of final analysis 10

3.7 Timing of outcome assessments 10

4. Sample size calculation 10

5. End of trial 10

6. Analysis principles 11

7. Handling of missing data 11

8. Analysis populations 11

9. Initial descriptive analyses 11

9.1 Recruitment, treatment allocation and follow-up 11

9.2 Baseline characteristics 12

9.3 Admission tests and microbiology results 15

9.4 Clinical management 16

9.5 Protocol adherence 17

9.6 Withdrawals 17
10. Primary analysis 17
11. Secondary analyses 18
11.1 Treatment failure at 48-hours following randomisation 18
11.2 Survival to 28 days following randomisation 19
11.3 Neurocognitive sequelae at 28 days following randomisation 19
11.4 Disability-free survival to 28 days following randomisation 19
11.5 Time to hypoxia resolution during initial hospital stay 20
11.6 Duration of respiratory support 20
11.7 Length of initial hospital stay 20
11.8 Re-admission to hospital by 28 days 20
11.9 Anthropometric status at 28 days following randomisation 20
11.10 Resolution of neurocognitive sequelae at 90 days 21
12. Subgroup analyses 21
13. Interim analyses 22
14. Timing of primary analysis 22
15. Safety 22
16. Statistical software 23
References 24

Abbreviations

COAST	Children's Oxygen Administration Strategies Trial
FiO_2	oxygen concentration
ICNARC	Intensive Care National Audit & Research Centre
IQR	interquartile range
LRTI	lower respiratory tract infection
MUAC	Mid upper arm circumference
SAE	serious adverse event
SD	standard deviation
SpO_2	oxygen saturation
WHO	World Health Organization
URTI	upper respiratory tract infection
VSP	very severe pneumonia

Glossary of terms

High flow oxygen	Flow rates 1 L/kg/min – 2 L/kg/min (up to 60 L/min max)
Low flow oxygen	Standard flow 1-2 L/min[1]
Respiratory distress	Deep breathing or increased work of breathing (indrawing)
Permissive hypoxia	Maintaining oxygen saturation (SpO_2) levels between 80-91%

1. Background

The Children's Oxygen Administration Strategies Trial (COAST) ["the Trial"] is a pragmatic, open, multicentre, fractional factorial randomised controlled trial in infants and children with respiratory distress complicated by hypoxia (defined as SpO_2 <92%).

This document describes the proposed statistical analyses for the Trial. It is important to set these out and agree on them in advance of analysing the outcome data for the Trial, so that data-derived decisions in the analyses are avoided. This statistical analysis plan has been prepared in accordance with recent published guidelines[1]. This document covers only the planned analysis for the main COAST trial as defined in protocol version 3.2, the analysis plans for the amended COAST-Nutrition trial will be defined in a separate document.

2. Objective and hypotheses

The primary objective of the Trial is to determine whether liberal oxygenation for SpO_2 ≥80% will decrease mortality at 48 hours compared with a strategy that includes permissive hypoxia (usual care); and whether use of high flow oxygen delivery will decrease mortality at 48 hours compared with low flow oxygen delivery (usual care). The Trial is formed of two strata, one for infants and children presenting with severe hypoxia (SpO_2 <80% - COAST A) and one for infants and children presenting with hypoxia (SpO_2 ≥80% and <92% - COAST B). The secondary objectives include comparing mortality at 28 days between the treatment groups within both strata.

The hypothesis of the Trial is that liberal oxygenation or high flow oxygen delivery will improve survival for infants and children presenting at hospital with hypoxia or severe hypoxia, respectively, at 48 hours.

3. Study design

3.1 Inclusion/exclusion criteria

The Trial population will consist of infants and children receiving treatment at a participating site who fulfil all of the inclusion criteria and none of the exclusion criteria below.

3.1.1 Inclusion criteria

- Aged between 28 days to 12 years
- History of respiratory illness (cough, upper respiratory tract symptom or any respiratory symptoms, e.g. rapid breathing or increase work of breathing)
- Hypoxia (pulse oximetry reading of SpO_2 <92% recorded in room air over 5 minutes) Plus suspected severe pneumonia informed by WHO guidelines, as below –
  a. Sign of respiratory distress (any one of):
     - severe lower chest wall in-drawing
     - use of auxiliary muscles
     - head nodding
     - inability to feed because of respiratory problems
  b. Suspected pneumonia
     - fast breathing:

        - age 2–11 months: ≥ 50/minute
        - age 1–5 years: ≥ 40/minute
        - age 5-12 years ≥ 30/minute
    - chest auscultation signs:
        - decreased breath sounds
        - bronchial breath sounds
        - crackles
        - abnormal vocal resonance (decreased over a pleural effusion or empyema, increased over lobar consolidation)
        - pleural rub

c. Signs of pneumonia with a general danger sign:
    - inability to breastfeed or drink
    - lethargy or unconscious
    - convulsions

3.1.2 Exclusion criteria

- Known uncorrected cyanotic heart disease
- Assent/consent refusal by parent/carer
- Previously recruited to COAST
- Already received oxygen for this episode of illness
- Known chronic lung disease (not including asthma)

3.2 Study Treatments

The treatments used in the Trial are described below.

Infants and children will be allocated into one of two strata COAST A or COAST B. Within COAST A infants and children will be randomised into oxygen delivery by high flow or low flow (1:1). Within COAST B infants and children will be randomised into oxygen delivery by high flow or low flow (liberal oxygenation) or permissive hypoxia (1:1:2).

3.2.1 COAST A

Infants and children that present with severe hypoxia (SpO_2<80% initially recorded over 5 minutes whilst breathing room air) will be allocated into COAST A.

3.2.1.1 Intervention group

Participants allocated to the COAST A intervention group will be delivered oxygen by high flow (flow rates 1 L/kg/min – 2 L/kg/min (up to 60 L/min max)) until SpO_2 ≥92% in room air.

The trial treatment period will last for a maximum of 48 hours following randomisation. After this time point, if oxygen is still required, the participant will be switched to oxygen delivery by low flow (i.e. standard of care).

During the intervention period, if an infant/child is unable to tolerate high flow oxygen (indicated by a poor modified Comfort B (behaviour) Scale score) and if oxygen is still required (i.e. failure to wean into room air), then the infant/child will be switched to oxygen delivery by low flow (i.e. standard of care).

3.2.1.2 Control group

Participants allocated to the COAST A control group will be delivered oxygen by low flow (standard flow 1-2 L/min[1]) until SpO_2 ≥92% in room air. The trial treatment period will last for a maximum of 48 hours following randomisation. After this time point, the participant will switch to usual care (standard clinical management).

If oxygen is discontinued before 48 hours e.g. hypoxia is resolved (SpO_2 ≥92% measured continuously over 30 minutes[2]), then they will switch to usual care (standard clinical management) following a successful trial of oxygen weaning.

3.2.2 COAST B

Infants and children that present with hypoxia (SpO_2 ≥80% and <92% which remains for 10 minutes, i.e. an additional 5 minutes to initial screening whilst breathing room air) will be allocated into COAST B.

If SpO_2 in the additional 5 minutes:

1. drops below 80%, infants and children will be allocated into COAST A and randomised accordingly
2. is ≥92%, infants and children will remain eligible for up to 24 hours following hospital admission (so long as the infant/child has not received oxygen).

3.2.2.1 Intervention groups

Participants allocated to the COAST B intervention group will be allocated liberal oxygenation. Infants and children will be delivered oxygen by low flow (standard flow 1-2 L/min[1]) or high flow (flow rates 1 L/kg/min – 2 L/kg/min (up to 60 L/min max)) until SpO_2 ≥92% in room air.

The trial treatment period will last for a maximum of 48 hours following randomisation. After this time point, the participant will switch to usual care (standard clinical management).

During the intervention period if an infant/child allocated to high flow oxygen delivery is unable to tolerate high flow oxygen (indicated by a poor modified Comfort B (behaviour) Scale score) and if oxygen is still required (i.e. failure to wean into room air), then the infant/child will be switched to oxygen delivery by low flow.

If oxygen is discontinued before 48 hours e.g. hypoxia is resolved (SpO_2 ≥92% measured continuously over 30 minutes[2]), then they will switch to usual care (standard clinical management) following a successful trial of oxygen weaning.

3.2.2.2 Control group

Participants allocated to the COAST B control group will be allocated to permissive hypoxia, maintaining SpO_2 levels between 80-91%. During the intervention period if an infant/child's SpO_2 ever drops below 80%, then the infant/child will be switched to oxygen delivery by low flow.

If oxygen is discontinued before 48 hours, e.g. hypoxia is resolved (SpO_2 ≥92% measured continuously over 30 minutes), then they will switch to usual care (standard clinical management) following a successful trial of oxygen weaning.

Once weaning has occurred further clinical and bedside monitoring will occur twice daily until hospital discharge.

All trial participants will receive standard of care including antibiotics (intravenous or oral) anti-malarial drugs following national guidelines, based on WHO syndromic patient management[3]. Usual emergency care will be provided as part of standard clinical management. All other care will be determined by the clinical team primarily responsible for the participant's care.

3.3 Randomisation

Participants will be allocated to treatment arms by a computer-generated list using random permuted blocks. The blocks will be stratified by trial site and baseline SaO_2 <80% or ≥80%. The Trial Statistician at the ICNARC Clinical Trials Unit (CTU) will prepare the randomisation lists before the trial commences and kept at the ICNARC CTU, London. Opaque and sealed randomisation envelopes will be prepared and provided to each site, with one set for COAST A and one set for COAST B The envelopes for each site will be numbered consecutively and opened in numerical order. These will contain details of the treatment arms once opened.

3.4 Outcome measures

3.4.1 Primary

The primary outcome measure is mortality within 48 hours following randomisation

3.4.2 Secondary

The secondary outcome measures are:

- Treatment failure, defined as continued SpO_2<92% for COAST A and SpO_2<80% for COAST B in the presence of respiratory distress (indrawing or intercostal retractions) at 48 hours following randomisation.
- Survival to 28 days following randomisation
- Neurocognitive sequelae (NS), defined as the development of a new neurocognitive deficit, assessed using the modified Kilifi Developmental Index, between the day of admission and 28 days following randomisation.
- Composite disability-free survival measured by the modified Kilifi Developmental Index, defined as a change to disability-free status, between the day of admission and 28 days following randomisation.
- Time to hypoxia resolution, defined as the duration in hours from randomisation to hypoxia resolution (SpO_2 ≥92% sustained over a period of 30 minutes during initial hospital stay)
- Duration of respiratory support, defined as the number of days alive and free from receipt of administered oxygen during the first 28 days following randomisation
- Length of initial hospital stay, defined as the duration in days from randomisation to initial hospital discharge
- Re-admission to hospital, defined as hospitalisation (at the trial centre or other health facility) for acute illness (i.e. not due to an existing illness or an elective admission) between discharge and 28 days following randomisation
- Anthropometric status, defined by Z-scores of mid-upper arm circumference and any evidence of visible severe wasting or signs of kwashiorkor at 28 days following randomisation

- Resolution of neurocognitive sequelae, defined as the full resolution by 90 days following randomisation of any neurocognitive deficit reported at 28 days following randomisation (for those with neurocognitive sequelae at 28 days)

3.5 Framework

All statistical tests will be for superiority. The trial hypothesis seeks to show superiority of liberal oxygenation vs. permissive hypoxia, and of high flow vs. low flow oxygen.

3.6 Timing of final analysis

The final analysis will be performed only after all patients recruited to the trial have completed their 28 day follow-up, and all required 90 day follow-up has been completed.

3.7 Timing of outcome assessments

All participants will be reassessed clinically at 1, 2, 4, 8, 12, 24 and 48 hours post-randomisation, and twice daily thereafter until discharged from hospital. Follow-up for secondary outcomes are scheduled at 28 days post randomisation, and at 90 days for patients with evidence of neurocognitive sequelae at 28 days. No restrictions on allowable visit windows are given in the protocol.

4. Sample size calculation

Sample size calculations were informed by identifying infants and children meeting the COAST inclusion criteria within two datasets: the FEAST trial (n=873/3170; 28%); and the Kilifi Hospital, Kenya admission cohort (n=2609/36,621; 7%). Based on these data, it was estimated that two thirds of eligible infants and children would present with SpO_2 ≥80% (FEAST 63%; Kilifi 69%). Baseline 48-hour mortality for infants and children receiving low flow oxygen was assumed to be 9% for SpO_2 ≥80% (FEAST 10%; Kilifi 9%) and 26% for SpO_2<80% (FEAST 30%; Kilifi 26%). Due to the complex nature of the design, power calculations were undertaken by simulating datasets under the assumed alternative hypotheses and calculating the proportion of simulated datasets in which a significant effect ($P<0.05$) was detected for each of the two comparisons[4]. Based on these simulations, a total sample size of 4,200 infants and children would give 90% power to detect a clinically relevant difference of a 33% relative risk reduction associated with liberal oxygenation compared with permissive hypoxia, and a clinically relevant difference of a 25% relative risk reduction for high flow compared with low flow oxygen delivery.

The sample size calculation is based on the primary outcome of mortality at 48 hours with no losses to follow-up expected. Losses to follow-up are anticipated to increase to 2% at 28 days following randomisation, based on data from the FEAST trial[5].

5. End of trial

The end of the trial will be when the final participant has completed their 28-day follow-up (or 90-day follow-up if applicable).

6. Analysis principles

All analyses will be conducted by intention to treat. The infants and children will be analysed according to the treatment group they were randomised to, irrespective of whether the treatment allocated was received, i.e. all infants and children will be included in the analysis, regardless of whether they have, or have not, adhered to the protocol. A two-sided p value of <0.05 will be taken to indicate a statistically significant result. No correction for multiple testing will be made. Effect estimates will be reported with 95% confidence intervals.

7. Handling of missing data

As the amount of missing data is anticipated to be minimal, a sensitivity approach will be taken when the primary outcome variable is missing. In the event that there are any missing outcomes at 48 hours, the primary analysis will be repeated once assuming that all infants and children allocated to the treatment groups with missing outcomes did not die within 48 hours, and all infants and children allocated to the control groups with missing outcomes did die within 48 hours. The analysis will then be repeated again with the opposite assumptions. This will then give the absolute range of how much the results could change if the primary outcome variable were complete.

In adjusted analyses missing baseline data will be handled by multiple imputation using the MICE (multivariate imputation by chained equations) algorithm. Five multiply imputed datasets will be created and results will be combined using Rubin's rules.

8. Analysis populations

All analyses will adhere to the intention to treat principle, unless otherwise specified in this SAP. The patients will be analysed according to the initial treatment assignment, irrespective of whether the allocated treatment was received. All patients for whom the primary outcome is known will be included in the analysis, regardless of protocol adherence.

9. Initial descriptive analyses

9.1 Recruitment, treatment allocation and follow-up

Recruitment to the Trial, treatment allocation and completeness of follow-up will be illustrated by a CONSORT flow diagram which will summarise the number of patients who were:

- Assessed for eligibility at screening
- Eligible at screening
- Ineligible at screening (with reasons where known)
- Eligible and randomised
- Eligible but not randomised (with reasons where known)
- Lost to follow-up before 48 hours (with reasons)

- Included in the primary analysis
- Lost to follow-up after 48 hours and before 28 days (with reasons)
- Assessed at 28 days, without known neurocognitive deficit (no further follow-up required)
- Assessed at 28 days, with known neurocognitive deficit
- Lost to follow-up after 28 days and before 90 days
- Assessed at 90 days

All participating sites have maintained a Screening Log of infants and children who are eligible (fulfil all of the inclusion criteria and none of the exclusion criteria) but not randomised, or who fulfil all of the inclusion criteria but meet one or more of the exclusion criteria. Reasons for non-recruitment will be categorised and summarised.

9.2 Baseline characteristics

The following baseline (pre-randomisation or within 1 hour of randomisation) demographic and clinical factors will be summarised by treatment group and strata but not subjected to statistical testing:

- Age in months – mean (sd) and median (IQR)
- Gender (male, female) – number (%)
- Weight for age Z score (calculated using the Stata macro igrowup_standard.ado[6]) – mean (sd) and median (IQR)
- Height for age Z score (calculated using the Stata macro igrowup_standard.ado) – mean (sd) and median (IQR)
- MUAC – mean (sd) and median (IQR)
- Any signs of malnutrition present (yes, no) – number (%)
  defined as: MUAC <11.5cm and aged >=6 months
  OR very severe wasting/marasmus
  OR signs of kwashiorkor
- Fever (temperature >37.5°C) – number (%)
- Hypothermia (temperature <36°C)– number (%)
- Initial SpO2 – mean (sd) and median (IQR)
- Initial SpO2 <70% , ≥70% - <80% (COAST A) – number (%)
- Initial SpO2 ≥80% - <85% , ≥85% - <92% (COAST B) – number (%)
- Central cyanosis– number (%)
- Respiratory rate – mean (sd) and median (IQR)
- Tachypnoea – number (%)

  defined as:

Age	RR
<2 months	>=60 breaths/min
2 to 11 months	>=50 breaths/min
1 to 5 years	>=40 breaths/min
6 to 12 years	>=30 breaths/min

- Bradycardia

  defined as:

Age	HR

<5 years	<80 bpm
≥5 years	<70 bpm

- Severe Tachycardia

  defined as:

Age	HR
<12 months	>180 bpm
≥1 year and <5 years	>160 bpm
≥5 years	>140 bpm

- Moderate hypotension

  defined as:

Age	SBP*
<12 months	50 to 75 mmHg
≥1 year and <5 years	60 to 75 mmHg
≥5 years	70 to 85 mmHg

* as measured with the use of an automated blood-pressure monitor

- Decompensated shock (yes, no) – number (%)

  Defined as severe hypotension (SBP<50 if age <12 months, or SBP <60 if 12 months ≤ age < 5 years, or SBP <70 if age ≥5 years)
- Compensated shock: Signs of Impaired perfusion: capillary refill time >=2 sec, temperature gradient, weak radial pulse, severe tachycardia.
- Responsiveness (AVPU) – number (%)
- Severity of respiratory distress, assessed using an age-adjusted integrated respiratory distress and wheeze score[7] scored from 0 (no distress) to a worst possible score of 9, calculated as the sum of the following items: Audible wheeze (0-3); Ability to vocalise (0-3); Ability to feed (0-3)

Clinical history of presenting illness

- Any signs of current febrile illness (Temperature>37.5 OR temperature <36) - number (%)
- History of fever
- History of cough- number (%)
- Difficulty breathing - number (%)
- Upper respiratory tract infection (Sore throat OR Earache/ear discharge) - number (%)
- Vomiting OR Diarrhoea - number (%)
- Any history of Fits in this illness OR fitting at physical examination - number (%)

Treatment of presenting illness

- Admitted for over 24 hours in another facility - number (%)
- Oral antimalarial treatment OR any Injections or infusion of anti-malarials in the last week - number (%)
- Oral antibiotics OR injections of antibiotics - number (%)
- Type of antibiotic injections - number (%) by type
- Inhalers OR Oral steroids, all children – number (%)
- Inhalers OR Oral steroids, in children with known asthma – number (%)

Clinical examination – number (%) with each of the following:

- In-drawing
- Deep breathing
- Grunting
- Crackles /crepitations on auscultation
- Audible wheeze
- Signs of dehydration present (Sunken eyes OR Decreased skin turgor)
- Splenomegaly (gross ≥ 5 cm)
- Altered conscious level
    - Prostration (unable to sit upright or breast feed if <6months)
    - Coma (unable to localise a painful stimulus or respond if <9 months)
- Suspected CNS infection (Neck stiffness or bulging fontanelle (infants only))

Past History – number (%) with each of the following:

- Gestation at birth <37 weeks
- Known HIV
- If yes, receiving antiretroviral therapy
- Previous or recent tuberculosis diagnosis
- Asthmatic (Known asthma OR Regular inhalers)
- Two or more hospital admissions in the last year
- Known epilepsy
- Previous developmental delay, defined as any one or more of the following:
  inability to walk unsupported in child aged > 18 months OR
  inability to sit unsupported in child aged > 6 months OR
  inability to suck (ever)
- Parental concerns about child's vision
- Parental concerns about child's hearing

Acute diagnosis

- Number of initial diagnoses (one vs two or more) – number (%)
- Diagnosis of: LRTI – number (%)
  URTI - number (%)
  Asthma - number (%)
  Other chest syndrome - number (%)
  Severe malaria – number (%)
  Sepsis/septicaemia – number (%)
  Sickle cell disease – number (%)
  Tuberculosis – number (%)
  Hepatitis – number (%)
  Meningitis - number (%)
  HIV/AIDS – number (%)
  Developmental delay/cerebral palsy – number (%)
  Encephalopathy – number (%)

Osteomyelitis/Pyogenic arthritis – number (%)
Urinary Tract Infection - number (%)
Blackwater fever (history of haemoglobinuria in this illness, or diagnosis of recurrent haemoglobinuria or dark urine syndrome) - number (%)
Severe anaemia – number (%)
Malnutrition – number (%)
Pyrexia of unknown origin – number (%)
Gastroenteritis – number (%)
Other – number (%)

Numbers and percentages within each treatment group will be reported for categorical factors; means (with SD) and medians (with IQR) within each treatment group will be reported for continuous factors.

9.3 Admission tests and microbiology results

The following admission and microbiology results will be summarised by treatment group and strata but not subjected to statistical testing: Not all microbiology results will be available in time for the primary analysis, these will be reported later on once available.

Haematology

- Haemoglobin - mean (sd) and median (IQR)
- Severe anaemia (Hb < 6g/dl) - number (%)
- WBC- mean (sd) and median (IQR)
- Leukocytosis (WBC > 11) - number (%)

Points of care

- Lactate > or = 5mmol/L - number (%)
- Glucose <3.0 - number (%)

Biochemistry

- Sodium- mean (sd) and median (IQR)
- Potassium- mean (sd) and median (IQR)
- Creatinine- mean (sd) and median (IQR)
- Urea- mean (sd) and median (IQR)

Malaria

- Current Malaria film positive - number (%)
- (Recent) Malaria RDT positive - number (%)

Microbiology

- Pathogen isolated - number (%)
- HIV positive - number (%)

Numbers and percentages within each treatment group will be reported for categorical factors; means (with SD) and medians (with IQR) within each treatment group will be reported for continuous factors.

9.4 Clinical management

Clinical management will be summarised using descriptive statistics by treatment group and stratum but not subjected to statistical testing.

For children randomised to high flow oxygen, summary measures of clinical management may include (but are not limited to)-:

- Number and percentage by initial model of oxygen delivery (low flow, high flow, none)
- Starting flow rate in those initiated to high flow – mean (sd) and median (IQR)
- Number and percentage initiated to high flow using room air only (O_2% of 21%)
- Number and percentage initiated to high flow with starting O_2% above 21%
- Starting O_2% in those initiated to high flow (for all, and for those with O_2 above 21%) – mean (sd) and median (IQR)
- Number and percentage of children with any dose escalation within the first 48 hours post randomisation (defined as high flow treatment with flow rate <u>or</u> O_2% above levels at initiation)
- maximum administered flow rate while on high flow – mean (sd) and median (IQR)
- maximum administered O_2% while on high flow – mean (sd) and median (IQR)
- Number and percentage with any problem giving high flow therapy, and number and percentage with each individual problem
- Total hours (within the first 48 hours) on high flow therapy – mean (sd) and median (IQR)
- Action taken at end of high flow therapy (started on low flow, off O_2 therapy, died while on high flow) – number (%)

For children randomised to low flow oxygen, summary measures of clinical management may include (but are not limited to)-:

- Number and percentage by initial model of oxygen delivery (low flow, high flow, none)
- Starting flow rate in those initiated to low flow – mean (sd) and median (IQR)
- Starting O_2% in those initiated to low flow – mean (sd) and median (IQR)
- Number and percentage of children with any dose escalation within the first 48 hours post randomisation (defined as low flow treatment with flow rate <u>or</u> O_2% above levels at initiation)
- maximum administered flow rate while on low flow – mean (sd) and median (IQR)
- maximum administered O_2% while on low flow – mean (sd) and median (IQR)
- Number and percentage with any problem giving low flow therapy, and number and percentage with each individual problem
- Total hours (within the first 48 hours) on low flow therapy – mean (sd) and median (IQR)
- Action taken at end of low flow therapy (started on high flow, off O_2 therapy, died while on low flow) – number (%)

For children randomised to no oxygen therapy, the following will be reported:

- Number and percentage by initial model of oxygen delivery (low flow, high flow, none)
- Oxygen therapy commenced within first 48 hours (low flow, high flow) – number (%)
- Time to commencing oxygen therapy (within first 48 hours) – mean (sd) and median (IQR)

The treatment groups will be compared for the following:

Mean (SD) oxygen saturation will be plotted graphically by treatment group and stratum at baseline and at the following time points following randomisation: 1 hour, 2 hours, 4 hours, 8 hours, 12 hours, 24 hours, 36 hours, 48 hours.

9.5 Protocol adherence

The following measured of protocol adherence within the first 48 hours of randomisation will be reported by treatment group and stratum:

Number and percentage of patients commencing on the randomised treatment

In patients commencing the randomised treatment, number and percentage changing treatment mode before 48 hours, overall and by reason

Flow rates and pressure will be summarised by time using graphical methods.

9.6 Withdrawals

The number and percentage of patients with written consent at or after randomisation will be reported, by stratum. Following randomisation, the number and percentage of patients, by arm, in the following groups will be summarised:

Consent never obtained, died without consent

Consent never obtained, absconded without consent

Consent declined (with reasons if known)

Withdrew following consent (with reasons if known)

10.Primary analysis

The number and percentage of deaths at 48 hours following randomisation will be reported by treatment group and stratum. The primary outcome will be analysed as a binary outcome using logistic regression including both treatment allocation variables (liberal oxygenation vs permissive hypoxia and high flow vs low flow) simultaneously and adjusted for the stratifying factors (baseline SpO_2 - categorised as <80%, 80-84%, 85-89%, 90-91% - and trial site). The primary effect estimate will be the odds ratio with 95% confidence interval adjusted for baseline SpO_2 and site, using only patients with known survival status by 48 hours. Each treatment allocation variable will be tested for significance by removing it in turn from the adjusted model

(adjusted for baseline SpO2 and site) and the resulting change in fit will be tested for significance using a likelihood ratio test.

An additional adjusted model will be fitted to adjust for baseline disease severity score (PETaL[8]), with baseline SpO2 and site. PETaL score is calculated by adding the coefficients in the table below for each factor present at baseline and summing the result.

Factor	Coefficient
Temperature<=37C	1.08
Heart rate <80bpm	2.46
Heart rate >=80 and <105	1.12
Heart rate >=220	1.44
Capillary refill time >=2 seconds	0.93
Prostrate (unable to sit unsupported or breastfeed if age <=6 month)	1.01
Coma (unable to localise (or respond to if age <=9 months) painful stimulus)	2.24
Respiratory distress (assumed present for all children)	0.93
Lung crepitations (crackles/crepitations)	0.77
Severe pallor	0.90
Weak pulse	1.45
Weight < 6kg	0.52
Weight >=6kg and <=8kg	0.30
Deep breathing	1.17

No further selection of covariates will be performed based on imbalance at baseline or significance in univariable analyses. The results of the logistic regression model will be reported as an adjusted odds ratio with a 95% confidence interval.

11.Secondary analyses

The secondary outcomes will be analysed using general linear models (logistic regression for binary outcomes, linear regression for continuous outcomes, Cox proportional hazards models for time-to-event outcomes) adjusted for the same baseline covariates as the primary analysis. Results of the general linear models will be reported as effect estimates (odds ratio, difference in means, hazard ratio) with 95% confidence intervals.

11.1 Treatment failure at 48-hours following randomisation

Treatment failure is defined as continued SpO_2<92% in the presence of respiratory distress at 48 hours following randomisation, or SpO_2<80% at any time during the first 48 hours for infants and children allocated to permissive hypoxia. Deaths at 48 hours will be treated as treatment failures. The number and percentage of treatment failures at 48 hours following randomisation will be reported by treatment group and stratum and analysed by logistic regression.

11.2 Survival to 28 days following randomisation

The number and percentage of infants and children surviving to their 28 day follow-up visit following randomisation will be reported by treatment group and stratum. Only 28 day follow-up visits which occur within 21 to 60 days of randomisation (inclusive) will be counted. Where a patient has no recorded 28 day visit, but does have a 90 day or other additional visit recorded within a range of 21-60 days of randomisation, the survival status at this visit will be used for this endpoint.

In addition, Kaplan Meier curves by treatment group and stratum will be plotted using all available follow-up data, and the estimated proportion (with 95% confidence interval) of survival at 28 days will be reported. Treatment effects will be analysed by Cox proportional hazards models. The proportional hazards assumption will be examined using log-log plots and tested using Schoenfeld residuals. In the event that the proportional hazards assumption is violated, alternative model structures will be considered, such stratifying on covariates which violate the proportional hazards model, partitioning follow-up time and fitting separate models, or introducing time-varying covariates or interactions.

11.3 Neurocognitive sequelae at 28 days following randomisation

Neurocognitive sequelae is defined as the development of a **new neurocognitive deficit**, assessed using the modified Kilifi Developmental Index, between the day of admission and 28 days following randomisation. The number and percentage of infants and children with Neurocognitive sequelae at 28 days following randomisation, among those alive at that time point, will be reported by treatment group and stratum and analysed using logistic regression. Status at 28 days is defined using allowable visit windows as described above for survival at 28 days. Patients are defined as having a new neurocognitive deficit if any of the following conditions are met:

CRFs v1.1 and before: fail on any of gross motor, fine motor or communication skills, or any one of abnormal speech production, comprehension, behaviour, movement/motor function, feeding difficulty, AND acute diagnosis of developmental delay/cerebral palsy is NOT recorded at baseline

CRFs v2.0: fail on any of gross motor, fine motor or communication skills, or any one of abnormal speech production, comprehension, behaviour, movement/motor function, feeding difficulty AND these are not recorded as pre-existing at baseline.

11.4 Disability-free survival to 28 days following randomisation

Composite disability-free survival is defined as either death or a change to disability-free status, between the day of admission and 28 day follow-up visit (defined using allowable visits windows as previously described) following randomisation. The number and percentage of infants and children with a change to disability-free status (or death) at 28 days following randomisation will be reported by treatment group and stratum and analysed by logistic regression. Development of a new disability is defined as either a new neurocognitive sequela as defined previously, or meeting any one or more of the following conditions:

Abnormal vision at 28 days, either recorded as new condition (on CRFs v2.0 and above) or with no parental concerns regarding vision at baseline (on CRFs v1.0)

Abnormal hearing at 28 days, recorded as new condition (CRFs v2.0 and above) or with no parental concerns regarding vision at baseline (CRFs v1.0)

11.5 Time to hypoxia resolution during initial hospital stay

Time to hypoxia resolution is defined as the duration in hours from randomisation to hypoxia resolution, defined as SpO_2 ≥92% measured continuously over a period of 30 minutes during initial hospital stay. The median (IQR) time to hypoxia resolution, among those for whom hypoxia resolved, will be reported by treatment group and stratum. Differences between the treatment groups will be compared using a Fine and Gray competing risks model to account for the competing risk of mortality - adjusted for the same baseline covariates as the primary analysis.

11.6 Duration of respiratory support

Duration of respiratory support is defined as the number of days alive and free from receipt of administered oxygen up to 28 days following randomisation. The mean (SD) number of days receiving administered oxygen will be reported by treatment group and stratum for survivors and non-survivors. The mean (SD) number of days alive and free from receipt of administered oxygen will be reported by treatment group and stratum, with infants and children that die within 28 days following randomisation considered to have zero days free from receipt of administered oxygen, and analysed using linear regression.

11.7 Length of initial hospital stay

Length of initial hospital stay is defined as the duration in days from randomisation to initial hospital discharge. The mean (SD) and median (IQR) number of days in hospital will be reported by treatment group and stratum, both overall and by survivors and non-survivors, and analysed using linear regression.

11.8 Re-admission to hospital by 28 days

Re-admission to hospital is defined as hospitalisation (at the trial centre or other health facility) for acute illness (i.e. not due to an existing illness or an elective admission) between discharge and 28 days following randomisation. The number and percentage of infants and children re-admitted to hospital by 28 days following randomisation, among those discharged alive from hospital, will be reported by treatment group and stratum and analysed using logistic regression.

11.9 Anthropometric status at 28 days following randomisation

Anthropometric status is defined by Z-scores of mid-upper arm circumference (MUAC) and any evidence of visible severe wasting or signs of kwashiorkor.

The mean Z scores for weight-for-height, weight-for-age, and MUAC at 28 days following randomisation, among those alive at 28 days, will be reported by treatment group and stratum and analysed using linear regression, adjusted for baseline variables as listed for the primary outcome, and also for baseline z scores. The number and percentage of infants and children with evidence of severe acute malnutrition (visible severe

wasting or signs of kwashiorkor) at 28 days following randomisation, among those alive at 28 days, will be reported by treatment group and stratum and analysed using logistic regression.

11.10 Resolution of neurocognitive sequelae at 90 days

Resolution of neurocognitive sequelae is defined as the full resolution by 90 days following randomisation of any neurocognitive deficit reported at 28 days following randomisation (for those with neurocognitive sequelae at 28 days, as defined in section 11.3 above). The number and percentage of infants and children with neurocognitive sequelae at 90 days following randomisation (or death by 90 days), among those with neurocognitive sequelae at 28 days, will be reported by treatment group and stratum and analysed using logistic regression. No restrictions will be placed on the allowed timing of the 90 day visit, but it must be later than the visit used as the 28 day visit. if no 90 day visit has been recorded but an additional visit at 60 or more days following visit has been recorded, this will be treated as the 90 day visit.

12.Subgroup analyses

Pre-specified subgroup analyses will assess whether the effects of the two interventions on the primary outcome vary according to categories of baseline SpO_2 (<80%, 80-84.9%, 85-89.9%, 90-91.9%) and by trial site. Additional hypothesis-generating analyses will investigate whether there is any evidence for a different impact of the interventions according to the following categorical variables:

fever (temperature>37.5 or < 36 °C at screening);

*malaria (defined as positive RDT or positive blood film on admission)

*Suspected sepsis: Leucocytosis: WBC > 11

*microbiological evidence of sepsis (blood culture or retrospective molecular diagnosis) ;

*Presence of any viral pathogens identified by NPA or molecular diagnostics,

radiographic evidence of pneumonia (signs recorded in right and/or left lung on chest X-ray consistent with pneumonia);

HIV;

Severe Malnutrition (MUAC <11.5cm; visible severe wasting or kwashiorkor)

*severe anaemia (haemoglobin <5g/dl);

*Sickle cell disease (known or diagnosed by PCR on admission sample)

Known or newly diagnosed congenital cardiac condition

Shock (compensated or decompensated, as previously defined in section 9.2)

Suspected gastroenteritis (vomiting and/or diarrhoea)

*these variables are obtained from blood & urine laboratory analysis, and are not expected to be available for the initial analysis.

In each case, the relevant subgroup variable will be added to an adjusted model (adjusting for site, treatment allocation, disease severity) and an interaction term between the subgroup variable and treatment allocation will be tested for significance. An estimated odds ratio with 95% confidence interval will be calculated for each subgroup.

13. Interim analyses

Three interim analyses were planned following recruitment and follow-up (to 48 hours) of 525, 1575 and 2625 infants and children. Guidelines to recommend early termination will be based on a Peto-Haybittle stopping rule ($P<0.001$). A recommendation to discontinue recruitment, in all participants or in selected subgroups, will be made only if the results are likely to convince the general clinical community and participants in COAST. Due to the stringent stopping criteria recommended, no adjustment will be made to the P value for the final analysis.

At the time of writing, two interim analyses had already been performed and reported to the DMC on 21st July 2017, after a total of 526 patients had been recruited and on 27th November 2019 after a total of 1645 children had been recruited to 25 October 2019. Recruitment continued until February 2020, when the trial steering group recommended that the trial should be stopped on the grounds of feasibility.

14.Timing of primary analysis

The primary clinical endpoint and all secondary clinical endpoints will be analysed after all patients have completed their 28 day follow-up visit. At this point all planned analysis not involving laboratory results will be performed (see sections9.3 and 12 for detail or data which is not anticipated to be available at this point) A further analysis of extra baseline characteristics and subgroups to include all biochemistry data will be performed once this data is available, but any analysis which had been run previously will not be repeated at this stage.

15.Safety

The number and percentage of Serious Adverse Events (SAEs) within 28 days following randomisation will be reported by treatment group and stratum. The types of SAEs, whether they were fatal or not and the perceived relatedness to the trial interventions will be summarised by treatment group and stratum. Cause of death will be adjudicated by an Endpoint Review Committee (ERC) blinded to randomised allocations. Relationship to all possible interventions will be solicited to avoid unblinding.

16.Statistical software

All analyses will be conducted in Stata/SE Version 14.2 64-bit x86-64 (StatCorp LLC, College Station, TX).

References

[1] Gamble C, Krishan A, Stocken D, et al. Guidelines for the Content of Statistical Analysis Plans in Clinical Trials. JAMA 2017;318:2337-43.

[2] Pocket Book of Hospital Care for Children: Guidelines for the management of common childhood illnesses. In., Second edn. Geneva: World Health Organization; 2013.

[3] Hospital Care for Children. Guidelines for the management of common childhood illnesses. Second Edition. In. Geneva: World Health Organization; 2013.

[4] Landau S, Stahl D: Sample size and power calculations for medical studies by simulation when closed form expressions are not available. Stat Methods Med Res 2013, in press.

[5] Maitland K, Kiguli S, Opoka RO, Engoru C, Olupot-Olupot P, Akech SO, Nyeko R, Mtove G, Reyburn H, Lang T, Brent B, Evans JA, Tibenderana JK, Crawley J, Russell EC, Levin M, Babiker AG, Gibb DM: Mortality after fluid bolus in African children with severe infection. The New England journal of medicine 2011, 364:2483-2495.

[6] http://www.who.int/childgrowth/software/en/

[7] Liu LL, Gallaher MM, Davis RL, Rutter CM, Lewis TC, Marcuse EK. Use of a respiratory clinical score among different providers. Pediatric pulmonology. 2004; 37(3): 243-8.

[8] George EC, Walker AS, Kiguli S et. Al. Predicting Mortality in Sick African Children: The FEAST Paediatric Emergency Triage (PET) Score. BMC Med, 13, 174 2015 Jul 31
